# Cancer LncRNA Census reveals evidence for deep functional conservation of long noncoding RNAs in tumorigenesis

**DOI:** 10.1038/s42003-019-0741-7

**Published:** 2020-02-05

**Authors:** Joana Carlevaro-Fita, Andrés Lanzós, Lars Feuerbach, Chen Hong, David Mas-Ponte, Jakob Skou Pedersen, PCAWG Drivers and Functional Interpretation Group, Federico Abascal, Samirkumar B. Amin, Gary D. Bader, Jonathan Barenboim, Rameen Beroukhim, Johanna Bertl, Keith A. Boroevich, Søren Brunak, Peter J. Campbell, Joana Carlevaro-Fita, Dimple Chakravarty, Calvin Wing Yiu Chan, Ken Chen, Jung Kyoon Choi, Jordi Deu-Pons, Priyanka Dhingra, Klev Diamanti, Lars Feuerbach, J. Lynn Fink, Nuno A. Fonseca, Joan Frigola, Carlo Gambacorti-Passerini, Dale W. Garsed, Mark Gerstein, Gad Getz, Abel Gonzalez-Perez, Qianyun Guo, Ivo G. Gut, David Haan, Mark P. Hamilton, Nicholas J. Haradhvala, Arif O. Harmanci, Mohamed Helmy, Carl Herrmann, Julian M. Hess, Asger Hobolth, Ermin Hodzic, Chen Hong, Henrik Hornshøj, Keren Isaev, Jose M. G. Izarzugaza, Rory Johnson, Todd A. Johnson, Malene Juul, Randi Istrup Juul, Andre Kahles, Abdullah Kahraman, Manolis Kellis, Ekta Khurana, Jaegil Kim, Jong K. Kim, Youngwook Kim, Jan Komorowski, Jan O. Korbel, Sushant Kumar, Andrés Lanzós, Erik Larsson, Michael S. Lawrence, Donghoon Lee, Kjong-Van Lehmann, Shantao Li, Xiaotong Li, Ziao Lin, Eric Minwei Liu, Lucas Lochovsky, Shaoke Lou, Tobias Madsen, Kathleen Marchal, Iñigo Martincorena, Alexander Martinez-Fundichely, Yosef E. Maruvka, Patrick D. McGillivray, William Meyerson, Ferran Muiños, Loris Mularoni, Hidewaki Nakagawa, Morten Muhlig Nielsen, Marta Paczkowska, Keunchil Park, Kiejung Park, Jakob Skou Pedersen, Oriol Pich, Tirso Pons, Sergio Pulido-Tamayo, Benjamin J Raphael, Jüri Reimand, Iker Reyes-Salazar, Matthew A. Reyna, Esther Rheinbay, Mark A. Rubin, Carlota Rubio-Perez, Radhakrishnan Sabarinathan, S. Cenk Sahinalp, Gordon Saksena, Leonidas Salichos, Chris Sander, Steven E. Schumacher, Mark Shackleton, Ofer Shapira, Ciyue Shen, Raunak Shrestha, Shimin Shuai, Nikos Sidiropoulos, Lina Sieverling, Nasa Sinnott-Armstrong, Lincoln D. Stein, Joshua M. Stuart, David Tamborero, Grace Tiao, Tatsuhiko Tsunoda, Husen M. Umer, Liis Uusküla-Reimand, Alfonso Valencia, Miguel Vazquez, Lieven P. C. Verbeke, Claes Wadelius, Lina Wadi, Jiayin Wang, Jonathan Warrell, Sebastian M. Waszak, Joachim Weischenfeldt, David A. Wheeler, Guanming Wu, Jun Yu, Jing Zhang, Xuanping Zhang, Yan Zhang, Zhongming Zhao, Lihua Zou, Christian von Mering, Rory Johnson, PCAWG Consortium, Lauri A. Aaltonen, Federico Abascal, Adam Abeshouse, Hiroyuki Aburatani, David J. Adams, Nishant Agrawal, Keun Soo Ahn, Sung-Min Ahn, Hiroshi Aikata, Rehan Akbani, Kadir C. Akdemir, Hikmat Al-Ahmadie, Sultan T. Al-Sedairy, Fatima Al-Shahrour, Malik Alawi, Monique Albert, Kenneth Aldape, Ludmil B. Alexandrov, Adrian Ally, Kathryn Alsop, Eva G. Alvarez, Fernanda Amary, Samirkumar B. Amin, Brice Aminou, Ole Ammerpohl, Matthew J. Anderson, Yeng Ang, Davide Antonello, Pavana Anur, Samuel Aparicio, Elizabeth L. Appelbaum, Yasuhito Arai, Axel Aretz, Koji Arihiro, Shun-ichi Ariizumi, Joshua Armenia, Laurent Arnould, Sylvia Asa, Yassen Assenov, Gurnit Atwal, Sietse Aukema, J. Todd Auman, Miriam R. R. Aure, Philip Awadalla, Marta Aymerich, Gary D. Bader, Adrian Baez-Ortega, Matthew H. Bailey, Peter J. Bailey, Miruna Balasundaram, Saianand Balu, Pratiti Bandopadhayay, Rosamonde E. Banks, Stefano Barbi, Andrew P. Barbour, Jonathan Barenboim, Jill Barnholtz-Sloan, Hugh Barr, Elisabet Barrera, John Bartlett, Javier Bartolome, Claudio Bassi, Oliver F. Bathe, Daniel Baumhoer, Prashant Bavi, Stephen B. Baylin, Wojciech Bazant, Duncan Beardsmore, Timothy A. Beck, Sam Behjati, Andreas Behren, Beifang Niu, Cindy Bell, Sergi Beltran, Christopher Benz, Andrew Berchuck, Anke K. Bergmann, Erik N. Bergstrom, Benjamin P. Berman, Daniel M. Berney, Stephan H. Bernhart, Rameen Beroukhim, Mario Berrios, Samantha Bersani, Johanna Bertl, Miguel Betancourt, Vinayak Bhandari, Shriram G. Bhosle, Andrew V. Biankin, Matthias Bieg, Darell Bigner, Hans Binder, Ewan Birney, Michael Birrer, Nidhan K. Biswas, Bodil Bjerkehagen, Tom Bodenheimer, Lori Boice, Giada Bonizzato, Johann S. De Bono, Arnoud Boot, Moiz S. Bootwalla, Ake Borg, Arndt Borkhardt, Keith A. Boroevich, Ivan Borozan, Christoph Borst, Marcus Bosenberg, Mattia Bosio, Jacqueline Boultwood, Guillaume Bourque, Paul C. Boutros, G. Steven Bova, David T. Bowen, Reanne Bowlby, David D. L. Bowtell, Sandrine Boyault, Rich Boyce, Jeffrey Boyd, Alvis Brazma, Paul Brennan, Daniel S. Brewer, Arie B. Brinkman, Robert G. Bristow, Russell R. Broaddus, Jane E. Brock, Malcolm Brock, Annegien Broeks, Angela N. Brooks, Denise Brooks, Benedikt Brors, Søren Brunak, Timothy J. C. Bruxner, Alicia L. Bruzos, Alex Buchanan, Ivo Buchhalter, Christiane Buchholz, Susan Bullman, Hazel Burke, Birgit Burkhardt, Kathleen H. Burns, John Busanovich, Carlos D. Bustamante, Adam P. Butler, Atul J. Butte, Niall J. Byrne, Anne-Lise Børresen-Dale, Samantha J. Caesar-Johnson, Andy Cafferkey, Declan Cahill, Claudia Calabrese, Carlos Caldas, Fabien Calvo, Niedzica Camacho, Peter J. Campbell, Elias Campo, Cinzia Cantù, Shaolong Cao, Thomas E. Carey, Joana Carlevaro-Fita, Rebecca Carlsen, Ivana Cataldo, Mario Cazzola, Jonathan Cebon, Robert Cerfolio, Dianne E. Chadwick, Dimple Chakravarty, Don Chalmers, Calvin Wing Yiu Chan, Kin Chan, Michelle Chan-Seng-Yue, Vishal S. Chandan, David K. Chang, Stephen J. Chanock, Lorraine A. Chantrill, Aurélien Chateigner, Nilanjan Chatterjee, Kazuaki Chayama, Hsiao-Wei Chen, Jieming Chen, Ken Chen, Yiwen Chen, Zhaohong Chen, Andrew D. Cherniack, Jeremy Chien, Yoke-Eng Chiew, Suet-Feung Chin, Juok Cho, Sunghoon Cho, Jung Kyoon Choi, Wan Choi, Christine Chomienne, Zechen Chong, Su Pin Choo, Angela Chou, Angelika N. Christ, Elizabeth L. Christie, Eric Chuah, Carrie Cibulskis, Kristian Cibulskis, Sara Cingarlini, Peter Clapham, Alexander Claviez, Sean Cleary, Nicole Cloonan, Marek Cmero, Colin C. Collins, Ashton A. Connor, Susanna L. Cooke, Colin S. Cooper, Leslie Cope, Vincenzo Corbo, Matthew G. Cordes, Stephen M. Cordner, Isidro Cortés-Ciriano, Kyle Covington, Prue A. Cowin, Brian Craft, David Craft, Chad J. Creighton, Yupeng Cun, Erin Curley, Ioana Cutcutache, Karolina Czajka, Bogdan Czerniak, Rebecca A. Dagg, Ludmila Danilova, Maria Vittoria Davi, Natalie R. Davidson, Helen Davies, Ian J. Davis, Brandi N. Davis-Dusenbery, Kevin J. Dawson, Francisco M. De La Vega, Ricardo De Paoli-Iseppi, Timothy Defreitas, Angelo P. Dei Tos, Olivier Delaneau, John A. Demchok, Jonas Demeulemeester, German M. Demidov, Deniz Demircioğlu, Nening M. Dennis, Robert E. Denroche, Stefan C. Dentro, Nikita Desai, Vikram Deshpande, Amit G. Deshwar, Christine Desmedt, Jordi Deu-Pons, Noreen Dhalla, Neesha C. Dhani, Priyanka Dhingra, Rajiv Dhir, Anthony DiBiase, Klev Diamanti, Li Ding, Shuai Ding, Huy Q. Dinh, Luc Dirix, HarshaVardhan Doddapaneni, Nilgun Donmez, Michelle T. Dow, Ronny Drapkin, Oliver Drechsel, Ruben M. Drews, Serge Serge, Tim Dudderidge, Ana Dueso-Barroso, Andrew J. Dunford, Michael Dunn, Lewis Jonathan Dursi, Fraser R. Duthie, Ken Dutton-Regester, Jenna Eagles, Douglas F. Easton, Stuart Edmonds, Paul A. Edwards, Sandra E. Edwards, Rosalind A. Eeles, Anna Ehinger, Juergen Eils, Roland Eils, Adel El-Naggar, Matthew Eldridge, Kyle Ellrott, Serap Erkek, Georgia Escaramis, Shadrielle M. G. Espiritu, Xavier Estivill, Dariush Etemadmoghadam, Jorunn E. Eyfjord, Bishoy M. Faltas, Daiming Fan, Yu Fan, William C. Faquin, Claudiu Farcas, Matteo Fassan, Aquila Fatima, Francesco Favero, Nodirjon Fayzullaev, Ina Felau, Sian Fereday, Martin L. Ferguson, Vincent Ferretti, Lars Feuerbach, Matthew A. Field, J. Lynn Fink, Gaetano Finocchiaro, Cyril Fisher, Matthew W. Fittall, Anna Fitzgerald, Rebecca C. Fitzgerald, Adrienne M. Flanagan, Neil E. Fleshner, Paul Flicek, John A. Foekens, Kwun M. Fong, Nuno A. Fonseca, Christopher S. Foster, Natalie S. Fox, Michael Fraser, Scott Frazer, Milana Frenkel-Morgenstern, William Friedman, Joan Frigola, Catrina C. Fronick, Akihiro Fujimoto, Masashi Fujita, Masashi Fukayama, Lucinda A. Fulton, Robert S. Fulton, Mayuko Furuta, P. Andrew Futreal, Anja Füllgrabe, Stacey B. Gabriel, Steven Gallinger, Carlo Gambacorti-Passerini, Jianjiong Gao, Shengjie Gao, Levi Garraway, Øystein Garred, Erik Garrison, Dale W. Garsed, Nils Gehlenborg, Josep L. L. Gelpi, Joshy George, Daniela S. Gerhard, Clarissa Gerhauser, Jeffrey E. Gershenwald, Mark Gerstein, Moritz Gerstung, Gad Getz, Mohammed Ghori, Ronald Ghossein, Nasra H. Giama, Richard A. Gibbs, Bob Gibson, Anthony J. Gill, Pelvender Gill, Dilip D. Giri, Dominik Glodzik, Vincent J. Gnanapragasam, Maria Elisabeth Goebler, Mary J. Goldman, Carmen Gomez, Santiago Gonzalez, Abel Gonzalez-Perez, Dmitry A. Gordenin, James Gossage, Kunihito Gotoh, Ramaswamy Govindan, Dorthe Grabau, Janet S. Graham, Robert C. Grant, Anthony R. Green, Eric Green, Liliana Greger, Nicola Grehan, Sonia Grimaldi, Sean M. Grimmond, Robert L. Grossman, Adam Grundhoff, Gunes Gundem, Qianyun Guo, Manaswi Gupta, Shailja Gupta, Ivo G. Gut, Marta Gut, Jonathan Göke, Gavin Ha, Andrea Haake, David Haan, Siegfried Haas, Kerstin Haase, James E. Haber, Nina Habermann, Faraz Hach, Syed Haider, Natsuko Hama, Freddie C. Hamdy, Anne Hamilton, Mark P. Hamilton, Leng Han, George B. Hanna, Martin Hansmann, Nicholas J. Haradhvala, Olivier Harismendy, Ivon Harliwong, Arif O. Harmanci, Eoghan Harrington, Takanori Hasegawa, David Haussler, Steve Hawkins, Shinya Hayami, Shuto Hayashi, D. Neil Hayes, Stephen J. Hayes, Nicholas K. Hayward, Steven Hazell, Yao He, Allison P. Heath, Simon C. Heath, David Hedley, Apurva M. Hegde, David I. Heiman, Michael C. Heinold, Zachary Heins, Lawrence E. Heisler, Eva Hellstrom-Lindberg, Mohamed Helmy, Seong Gu Heo, Austin J. Hepperla, José María Heredia-Genestar, Carl Herrmann, Peter Hersey, Julian M. Hess, Holmfridur Hilmarsdottir, Jonathan Hinton, Satoshi Hirano, Nobuyoshi Hiraoka, Katherine A. Hoadley, Asger Hobolth, Ermin Hodzic, Jessica I. Hoell, Steve Hoffmann, Oliver Hofmann, Andrea Holbrook, Aliaksei Z. Holik, Michael A. Hollingsworth, Oliver Holmes, Robert A. Holt, Chen Hong, Eun Pyo Hong, Jongwhi H. Hong, Gerrit K. Hooijer, Henrik Hornshøj, Fumie Hosoda, Yong Hou, Volker Hovestadt, William Howat, Alan P. Hoyle, Ralph H. Hruban, Jianhong Hu, Taobo Hu, Xing Hua, Kuan-lin Huang, Mei Huang, Mi Ni Huang, Vincent Huang, Yi Huang, Wolfgang Huber, Thomas J. Hudson, Michael Hummel, Jillian A. Hung, David Huntsman, Ted R. Hupp, Jason Huse, Matthew R. Huska, Barbara Hutter, Carolyn M. Hutter, Daniel Hübschmann, Christine A. Iacobuzio-Donahue, Charles David Imbusch, Marcin Imielinski, Seiya Imoto, William B. Isaacs, Keren Isaev, Shumpei Ishikawa, Murat Iskar, S. M. Ashiqul Islam, Michael Ittmann, Sinisa Ivkovic, Jose M. G. Izarzugaza, Jocelyne Jacquemier, Valerie Jakrot, Nigel B. Jamieson, Gun Ho Jang, Se Jin Jang, Joy C. Jayaseelan, Reyka Jayasinghe, Stuart R. Jefferys, Karine Jegalian, Jennifer L. Jennings, Seung-Hyup Jeon, Lara Jerman, Yuan Ji, Wei Jiao, Peter A. Johansson, Amber L. Johns, Jeremy Johns, Rory Johnson, Todd A. Johnson, Clemency Jolly, Yann Joly, Jon G. Jonasson, Corbin D. Jones, David R. Jones, David T. W. Jones, Nic Jones, Steven J. M. Jones, Jos Jonkers, Young Seok Ju, Hartmut Juhl, Jongsun Jung, Malene Juul, Randi Istrup Juul, Sissel Juul, Natalie Jäger, Rolf Kabbe, Andre Kahles, Abdullah Kahraman, Vera B. Kaiser, Hojabr Kakavand, Sangeetha Kalimuthu, Christof von Kalle, Koo Jeong Kang, Katalin Karaszi, Beth Karlan, Rosa Karlić, Dennis Karsch, Katayoon Kasaian, Karin S. Kassahn, Hitoshi Katai, Mamoru Kato, Hiroto Katoh, Yoshiiku Kawakami, Jonathan D. Kay, Stephen H. Kazakoff, Marat D. Kazanov, Maria Keays, Electron Kebebew, Richard F. Kefford, Manolis Kellis, James G. Kench, Catherine J. Kennedy, Jules N. A. Kerssemakers, David Khoo, Vincent Khoo, Narong Khuntikeo, Ekta Khurana, Helena Kilpinen, Hark Kyun Kim, Hyung-Lae Kim, Hyung-Yong Kim, Hyunghwan Kim, Jaegil Kim, Jihoon Kim, Jong K. Kim, Youngwook Kim, Tari A. King, Wolfram Klapper, Kortine Kleinheinz, Leszek J. Klimczak, Stian Knappskog, Michael Kneba, Bartha M. Knoppers, Youngil Koh, Jan Komorowski, Daisuke Komura, Mitsuhiro Komura, Gu Kong, Marcel Kool, Jan O. Korbel, Viktoriya Korchina, Andrey Korshunov, Michael Koscher, Roelof Koster, Zsofia Kote-Jarai, Antonios Koures, Milena Kovacevic, Barbara Kremeyer, Helene Kretzmer, Markus Kreuz, Savitri Krishnamurthy, Dieter Kube, Kiran Kumar, Pardeep Kumar, Sushant Kumar, Yogesh Kumar, Ritika Kundra, Kirsten Kübler, Ralf Küppers, Jesper Lagergren, Phillip H. Lai, Peter W. Laird, Sunil R. Lakhani, Christopher M. Lalansingh, Emilie Lalonde, Fabien C. Lamaze, Adam Lambert, Eric Lander, Pablo Landgraf, Luca Landoni, Anita Langerød, Andrés Lanzós, Denis Larsimont, Erik Larsson, Mark Lathrop, Loretta M. S. Lau, Chris Lawerenz, Rita T. Lawlor, Michael S. Lawrence, Alexander J. Lazar, Ana Mijalkovic Lazic, Xuan Le, Darlene Lee, Donghoon Lee, Eunjung Alice Lee, Hee Jin Lee, Jake June-Koo Lee, Jeong-Yeon Lee, Juhee Lee, Ming Ta Michael Lee, Henry Lee-Six, Kjong-Van Lehmann, Hans Lehrach, Dido Lenze, Conrad R. Leonard, Daniel A. Leongamornlert, Ignaty Leshchiner, Louis Letourneau, Ivica Letunic, Douglas A. Levine, Lora Lewis, Tim Ley, Chang Li, Constance H. Li, Haiyan Irene Li, Jun Li, Lin Li, Shantao Li, Siliang Li, Xiaobo Li, Xiaotong Li, Xinyue Li, Yilong Li, Han Liang, Sheng-Ben Liang, Peter Lichter, Pei Lin, Ziao Lin, W. M. Linehan, Ole Christian Lingjærde, Dongbing Liu, Eric Minwei Liu, Fei-Fei Fei Liu, Fenglin Liu, Jia Liu, Xingmin Liu, Julie Livingstone, Dimitri Livitz, Naomi Livni, Lucas Lochovsky, Markus Loeffler, Georgina V. Long, Armando Lopez-Guillermo, Shaoke Lou, David N. Louis, Laurence B. Lovat, Yiling Lu, Yong-Jie Lu, Youyong Lu, Claudio Luchini, Ilinca Lungu, Xuemei Luo, Hayley J. Luxton, Andy G. Lynch, Lisa Lype, Cristina López, Carlos López-Otín, Eric Z. Ma, Yussanne Ma, Gaetan MacGrogan, Shona MacRae, Geoff Macintyre, Tobias Madsen, Kazuhiro Maejima, Andrea Mafficini, Dennis T. Maglinte, Arindam Maitra, Partha P. Majumder, Luca Malcovati, Salem Malikic, Giuseppe Malleo, Graham J. Mann, Luisa Mantovani-Löffler, Kathleen Marchal, Giovanni Marchegiani, Elaine R. Mardis, Adam A. Margolin, Maximillian G. Marin, Florian Markowetz, Julia Markowski, Jeffrey Marks, Tomas Marques-Bonet, Marco A. Marra, Luke Marsden, John W. M. Martens, Sancha Martin, Jose I. Martin-Subero, Iñigo Martincorena, Alexander Martinez-Fundichely, Yosef E. Maruvka, R. Jay Mashl, Charlie E. Massie, Thomas J. Matthew, Lucy Matthews, Erik Mayer, Simon Mayes, Michael Mayo, Faridah Mbabaali, Karen McCune, Ultan McDermott, Patrick D. McGillivray, Michael D. McLellan, John D. McPherson, John R. McPherson, Treasa A. McPherson, Samuel R. Meier, Alice Meng, Shaowu Meng, Andrew Menzies, Neil D. Merrett, Sue Merson, Matthew Meyerson, William Meyerson, Piotr A. Mieczkowski, George L. Mihaiescu, Sanja Mijalkovic, Tom Mikkelsen, Michele Milella, Linda Mileshkin, Christopher A. Miller, David K. Miller, Jessica K. Miller, Gordon B. Mills, Ana Milovanovic, Sarah Minner, Marco Miotto, Gisela Mir Arnau, Lisa Mirabello, Chris Mitchell, Thomas J. Mitchell, Satoru Miyano, Naoki Miyoshi, Shinichi Mizuno, Fruzsina Molnár-Gábor, Malcolm J. Moore, Richard A. Moore, Sandro Morganella, Quaid D. Morris, Carl Morrison, Lisle E. Mose, Catherine D. Moser, Ferran Muiños, Loris Mularoni, Andrew J. Mungall, Karen Mungall, Elizabeth A. Musgrove, Ville Mustonen, David Mutch, Francesc Muyas, Donna M. Muzny, Alfonso Muñoz, Jerome Myers, Ola Myklebost, Peter Möller, Genta Nagae, Adnan M. Nagrial, Hardeep K. Nahal-Bose, Hitoshi Nakagama, Hidewaki Nakagawa, Hiromi Nakamura, Toru Nakamura, Kaoru Nakano, Tannistha Nandi, Jyoti Nangalia, Mia Nastic, Arcadi Navarro, Fabio C. P. Navarro, David E. Neal, Gerd Nettekoven, Felicity Newell, Steven J. Newhouse, Yulia Newton, Alvin Wei Tian Ng, Anthony Ng, Jonathan Nicholson, David Nicol, Yongzhan Nie, G. Petur Nielsen, Morten Muhlig Nielsen, Serena Nik-Zainal, Michael S. Noble, Katia Nones, Paul A. Northcott, Faiyaz Notta, Brian D. O’Connor, Peter O’Donnell, Maria O’Donovan, Sarah O’Meara, Brian Patrick O’Neill, J. Robert O’Neill, David Ocana, Angelica Ochoa, Layla Oesper, Christopher Ogden, Hideki Ohdan, Kazuhiro Ohi, Lucila Ohno-Machado, Karin A. Oien, Akinyemi I. Ojesina, Hidenori Ojima, Takuji Okusaka, Larsson Omberg, Choon Kiat Ong, Stephan Ossowski, German Ott, B. F. Francis Ouellette, Christine P’ng, Marta Paczkowska, Salvatore Paiella, Chawalit Pairojkul, Marina Pajic, Qiang Pan-Hammarström, Elli Papaemmanuil, Irene Papatheodorou, Nagarajan Paramasivam, Ji Wan Park, Joong-Won Park, Keunchil Park, Kiejung Park, Peter J. Park, Joel S. Parker, Simon L. Parsons, Harvey Pass, Danielle Pasternack, Alessandro Pastore, Ann-Marie Patch, Iris Pauporté, Antonio Pea, John V. Pearson, Chandra Sekhar Pedamallu, Jakob Skou Pedersen, Paolo Pederzoli, Martin Peifer, Nathan A. Pennell, Charles M. Perou, Marc D. Perry, Gloria M. Petersen, Myron Peto, Nicholas Petrelli, Robert Petryszak, Stefan M. Pfister, Mark Phillips, Oriol Pich, Hilda A. Pickett, Todd D. Pihl, Nischalan Pillay, Sarah Pinder, Mark Pinese, Andreia V. Pinho, Esa Pitkänen, Xavier Pivot, Elena Piñeiro-Yáñez, Laura Planko, Christoph Plass, Paz Polak, Tirso Pons, Irinel Popescu, Olga Potapova, Aparna Prasad, Shaun R. Preston, Manuel Prinz, Antonia L. Pritchard, Stephenie D. Prokopec, Elena Provenzano, Xose S. Puente, Sonia Puig, Montserrat Puiggròs, Sergio Pulido-Tamayo, Gulietta M. Pupo, Colin A. Purdie, Michael C. Quinn, Raquel Rabionet, Janet S. Rader, Bernhard Radlwimmer, Petar Radovic, Benjamin Raeder, Keiran M. Raine, Manasa Ramakrishna, Kamna Ramakrishnan, Suresh Ramalingam, Benjamin J. Raphael, W. Kimryn Rathmell, Tobias Rausch, Guido Reifenberger, Jüri Reimand, Jorge Reis-Filho, Victor Reuter, Iker Reyes-Salazar, Matthew A. Reyna, Sheila M. Reynolds, Esther Rheinbay, Yasser Riazalhosseini, Andrea L. Richardson, Julia Richter, Matthew Ringel, Markus Ringnér, Yasushi Rino, Karsten Rippe, Jeffrey Roach, Lewis R. Roberts, Nicola D. Roberts, Steven A. Roberts, A. Gordon Robertson, Alan J. Robertson, Javier Bartolomé Rodriguez, Bernardo Rodriguez-Martin, F. Germán Rodríguez-González, Michael H. A. Roehrl, Marius Rohde, Hirofumi Rokutan, Gilles Romieu, Ilse Rooman, Tom Roques, Daniel Rosebrock, Mara Rosenberg, Philip C. Rosenstiel, Andreas Rosenwald, Edward W. Rowe, Romina Royo, Steven G. Rozen, Yulia Rubanova, Mark A. Rubin, Carlota Rubio-Perez, Vasilisa A. Rudneva, Borislav C. Rusev, Andrea Ruzzenente, Gunnar Rätsch, Radhakrishnan Sabarinathan, Veronica Y. Sabelnykova, Sara Sadeghi, S. Cenk Sahinalp, Natalie Saini, Mihoko Saito-Adachi, Gordon Saksena, Adriana Salcedo, Roberto Salgado, Leonidas Salichos, Richard Sallari, Charles Saller, Roberto Salvia, Michelle Sam, Jaswinder S. Samra, Francisco Sanchez-Vega, Chris Sander, Grant Sanders, Rajiv Sarin, Iman Sarrafi, Aya Sasaki-Oku, Torill Sauer, Guido Sauter, Robyn P. M. Saw, Maria Scardoni, Christopher J. Scarlett, Aldo Scarpa, Ghislaine Scelo, Dirk Schadendorf, Jacqueline E. Schein, Markus B. Schilhabel, Matthias Schlesner, Thorsten Schlomm, Heather K. Schmidt, Sarah-Jane Schramm, Stefan Schreiber, Nikolaus Schultz, Steven E. Schumacher, Roland F. Schwarz, Richard A. Scolyer, David Scott, Ralph Scully, Raja Seethala, Ayellet V. Segre, Iris Selander, Colin A. Semple, Yasin Senbabaoglu, Subhajit Sengupta, Elisabetta Sereni, Stefano Serra, Dennis C. Sgroi, Mark Shackleton, Nimish C. Shah, Sagedeh Shahabi, Catherine A. Shang, Ping Shang, Ofer Shapira, Troy Shelton, Ciyue Shen, Hui Shen, Rebecca Shepherd, Ruian Shi, Yan Shi, Yu-Jia Shiah, Tatsuhiro Shibata, Juliann Shih, Eigo Shimizu, Kiyo Shimizu, Seung Jun Shin, Yuichi Shiraishi, Tal Shmaya, Ilya Shmulevich, Solomon I. Shorser, Charles Short, Raunak Shrestha, Suyash S. Shringarpure, Craig Shriver, Shimin Shuai, Nikos Sidiropoulos, Reiner Siebert, Anieta M. Sieuwerts, Lina Sieverling, Sabina Signoretti, Katarzyna O. Sikora, Michele Simbolo, Ronald Simon, Janae V. Simons, Jared T. Simpson, Peter T. Simpson, Samuel Singer, Nasa Sinnott-Armstrong, Payal Sipahimalani, Tara J. Skelly, Marcel Smid, Jaclyn Smith, Karen Smith-McCune, Nicholas D. Socci, Heidi J. Sofia, Matthew G. Soloway, Lei Song, Anil K. Sood, Sharmila Sothi, Christos Sotiriou, Cameron M. Soulette, Paul N. Span, Paul T. Spellman, Nicola Sperandio, Andrew J. Spillane, Oliver Spiro, Jonathan Spring, Johan Staaf, Peter F. Stadler, Peter Staib, Stefan G. Stark, Lucy Stebbings, Ólafur Andri Stefánsson, Oliver Stegle, Lincoln D. Stein, Alasdair Stenhouse, Chip Stewart, Stephan Stilgenbauer, Miranda D. Stobbe, Michael R. Stratton, Jonathan R. Stretch, Adam J. Struck, Joshua M. Stuart, Henk G. Stunnenberg, Hong Su, Xiaoping Su, Ren X. Sun, Stephanie Sungalee, Hana Susak, Akihiro Suzuki, Fred Sweep, Monika Szczepanowski, Holger Sültmann, Takashi Yugawa, Angela Tam, David Tamborero, Benita Kiat Tee Tan, Donghui Tan, Patrick Tan, Hiroko Tanaka, Hirokazu Taniguchi, Tomas J. Tanskanen, Maxime Tarabichi, Roy Tarnuzzer, Patrick Tarpey, Morgan L. Taschuk, Kenji Tatsuno, Simon Tavaré, Darrin F. Taylor, Amaro Taylor-Weiner, Jon W. Teague, Bin Tean Teh, Varsha Tembe, Javier Temes, Kevin Thai, Sarah P. Thayer, Nina Thiessen, Gilles Thomas, Sarah Thomas, Alan Thompson, Alastair M. Thompson, John F. F. Thompson, R. Houston Thompson, Heather Thorne, Leigh B. Thorne, Adrian Thorogood, Grace Tiao, Nebojsa Tijanic, Lee E. Timms, Roberto Tirabosco, Marta Tojo, Stefania Tommasi, Christopher W. Toon, Umut H. Toprak, David Torrents, Giampaolo Tortora, Jörg Tost, Yasushi Totoki, David Townend, Nadia Traficante, Isabelle Treilleux, Jean-Rémi Trotta, Lorenz H. P. Trümper, Ming Tsao, Tatsuhiko Tsunoda, Jose M. C. Tubio, Olga Tucker, Richard Turkington, Daniel J. Turner, Andrew Tutt, Masaki Ueno, Naoto T. Ueno, Christopher Umbricht, Husen M. Umer, Timothy J. Underwood, Lara Urban, Tomoko Urushidate, Tetsuo Ushiku, Liis Uusküla-Reimand, Alfonso Valencia, David J. Van Den Berg, Steven Van Laere, Peter Van Loo, Erwin G. Van Meir, Gert G. Van den Eynden, Theodorus Van der Kwast, Naveen Vasudev, Miguel Vazquez, Ravikiran Vedururu, Umadevi Veluvolu, Shankar Vembu, Lieven P. C. Verbeke, Peter Vermeulen, Clare Verrill, Alain Viari, David Vicente, Caterina Vicentini, K. VijayRaghavan, Juris Viksna, Ricardo E. Vilain, Izar Villasante, Anne Vincent-Salomon, Tapio Visakorpi, Douglas Voet, Paresh Vyas, Ignacio Vázquez-García, Nick M. Waddell, Nicola Waddell, Claes Wadelius, Lina Wadi, Rabea Wagener, Jeremiah A. Wala, Jian Wang, Jiayin Wang, Linghua Wang, Qi Wang, Wenyi Wang, Yumeng Wang, Zhining Wang, Paul M. Waring, Hans-Jörg Warnatz, Jonathan Warrell, Anne Y. Warren, Sebastian M. Waszak, David C. Wedge, Dieter Weichenhan, Paul Weinberger, John N. Weinstein, Joachim Weischenfeldt, Daniel J. Weisenberger, Ian Welch, Michael C. Wendl, Johannes Werner, Justin P. Whalley, David A. Wheeler, Hayley C. Whitaker, Dennis Wigle, Matthew D. Wilkerson, Ashley Williams, James S. Wilmott, Gavin W. Wilson, Julie M. Wilson, Richard K. Wilson, Boris Winterhoff, Jeffrey A. Wintersinger, Maciej Wiznerowicz, Stephan Wolf, Bernice H. Wong, Tina Wong, Winghing Wong, Youngchoon Woo, Scott Wood, Bradly G. Wouters, Adam J. Wright, Derek W. Wright, Mark H. Wright, Chin-Lee Wu, Dai-Ying Wu, Guanming Wu, Jianmin Wu, Kui Wu, Yang Wu, Zhenggang Wu, Liu Xi, Tian Xia, Qian Xiang, Xiao Xiao, Rui Xing, Heng Xiong, Qinying Xu, Yanxun Xu, Hong Xue, Shinichi Yachida, Sergei Yakneen, Rui Yamaguchi, Takafumi N. Yamaguchi, Masakazu Yamamoto, Shogo Yamamoto, Hiroki Yamaue, Fan Yang, Huanming Yang, Jean Y. Yang, Liming Yang, Lixing Yang, Shanlin Yang, Tsun-Po Yang, Yang Yang, Xiaotong Yao, Marie-Laure Yaspo, Lucy Yates, Christina Yau, Chen Ye, Kai Ye, Venkata D. Yellapantula, Christopher J. Yoon, Sung-Soo Yoon, Fouad Yousif, Jun Yu, Kaixian Yu, Willie Yu, Yingyan Yu, Ke Yuan, Yuan Yuan, Denis Yuen, Christina K. Yung, Olga Zaikova, Jorge Zamora, Marc Zapatka, Jean C. Zenklusen, Thorsten Zenz, Nikolajs Zeps, Cheng-Zhong Zhang, Fan Zhang, Hailei Zhang, Hongwei Zhang, Hongxin Zhang, Jiashan Zhang, Jing Zhang, Junjun Zhang, Xiuqing Zhang, Xuanping Zhang, Yan Zhang, Zemin Zhang, Zhongming Zhao, Liangtao Zheng, Xiuqing Zheng, Wanding Zhou, Yong Zhou, Bin Zhu, Hongtu Zhu, Jingchun Zhu, Shida Zhu, Lihua Zou, Xueqing Zou, Anna deFazio, Nicholas van As, Carolien H. M. van Deurzen, Marc J. van de Vijver, L. van’t Veer, Christian von Mering

**Affiliations:** 1 Department of Medical Oncology, Inselspital, University Hospital and University of Bern, 3010 Bern, Switzerland; 2 Department of Biomedical Research, University of Bern, 3008 Bern, Switzerland; 3 Graduate School for Cellular and Biomedical Sciences, University of Bern, 3012 Bern, Switzerland; 4 Applied Bioinformatics, Deutsches Krebsforschungszentrum, 69120 Heidelberg, Germany; 5 Centre for Genomic Regulation (CRG), The Barcelona Institute of Science and Technology, Dr. Aiguader 88, Barcelona, 08003 Spain; 6 Universitat Pompeu Fabra (UPF), Barcelona, Spain; 7 Institut Hospital del Mar d’Investigacions Mèdiques (IMIM), Dr. Aiguader 88, 08003 Barcelona, Spain; 8 Department for Molecular Medicine, Aarhus University Hospital, Palle Juul-Jensens Boulevard 99, 8200 Aarhus N, Denmark; 9 Wellcome Sanger Institute, Wellcome Genome Campus, Hinxton, Cambridge, CB10 1SA UK; 10 Department of Genomic Medicine, The University of Texas MD Anderson Cancer Center, Houston, TX 77030 USA; 11 The Jackson Laboratory for Genomic Medicine, Farmington, CT 06032 USA; 12 Quantitative & Computational Biosciences Graduate Program, Baylor College of Medicine, Houston, TX 77030 USA; 13 Department of Molecular Genetics, University of Toronto, Toronto, ON M5S 1A8 Canada; 14 Computational Biology Program, Ontario Institute for Cancer Research, Toronto, ON M5G 0A3 Canada; 15 Broad Institute of MIT and Harvard, Cambridge, MA 02142 USA; 16 Department of Medical Oncology, Dana-Farber Cancer Institute, Boston, MA 02115 USA; 17 Harvard Medical School, Boston, MA 02115 USA; 18 Department of Mathematics, Aarhus University, Aarhus, 8000 Denmark; 19 Laboratory for Medical Science Mathematics, RIKEN Center for Integrative Medical Sciences, Yokohama, Kanagawa 230-0045 Japan; 20 RIKEN Center for Integrative Medical Sciences, Yokohama, Kanagawa 230-0045 Japan; 21 Technical University of Denmark, Lyngby, 2800 Denmark; 22 University of Copenhagen, Copenhagen, 2200 Denmark; 23 Department of Haematology, University of Cambridge, Cambridge, CB2 2XY UK; 24 Department of Genitourinary Medical Oncology - Research, Division of Cancer Medicine, The University of Texas MD Anderson Cancer Center, Houston, TX 77030 USA; 25 Division of Theoretical Bioinformatics, German Cancer Research Center (DKFZ), Heidelberg, 69120 Germany; 26 Faculty of Biosciences, Heidelberg University, Heidelberg, 69120 Germany; 27 University of Texas MD Anderson Cancer Center, Houston, TX 77030 USA; 28 Korea Advanced Institute of Science and Technology, Daejeon, 34141 South Korea; 29 Institute for Research in Biomedicine (IRB Barcelona), The Barcelona Institute of Science and Technology, Barcelona, 8003 Spain; 30 Research Program on Biomedical Informatics, Universitat Pompeu Fabra, Barcelona, 08002 Spain; 31 Department of Physiology and Biophysics, Weill Cornell Medicine, New York, NY 10065 USA; 32 Institute for Computational Biomedicine, Weill Cornell Medicine, New York, NY 10021 USA; 33 Science for Life Laboratory, Department of Cell and Molecular Biology, Uppsala University, Uppsala, SE-75124 Sweden; 34 Barcelona Supercomputing Center, Barcelona, 08034 Spain; 35 Queensland Centre for Medical Genomics, Institute for Molecular Bioscience, The University of Queensland, St Lucia, QLD 4072 Australia; 36 CIBIO/InBIO - Research Center in Biodiversity and Genetic Resources, Universidade do Porto, Vairão, 4485-601 Portugal; 37 European Molecular Biology Laboratory, European Bioinformatics Institute (EMBL-EBI), Wellcome Genome Campus, Hinxton, Cambridge, CB10 1SD UK; 38 University of Milano Bicocca, Monza, 20052 Italy; 39 Peter MacCallum Cancer Centre, Melbourne, VIC 3000 Australia; 40 Sir Peter MacCallum Department of Oncology, The University of Melbourne, Melbourne, VIC 3052 Australia; 41 Department of Computer Science, Princeton University, Princeton, NJ 08540 USA; 42 Department of Computer Science, Yale University, New Haven, CT 06520 USA; 43 Department of Molecular Biophysics and Biochemistry, Yale University, New Haven, CT 06520 USA; 44 Program in Computational Biology and Bioinformatics, Yale University, New Haven, CT 06520 USA; 45 Center for Cancer Research, Massachusetts General Hospital, Boston, MA 02129 USA; 46 Department of Pathology, Massachusetts General Hospital, Boston, MA 02115 USA; 47 Bioinformatics Research Centre (BiRC), Aarhus University, Aarhus, 8000 Denmark; 48 CNAG-CRG, Centre for Genomic Regulation (CRG), Barcelona Institute of Science and Technology (BIST), Barcelona, 08028 Spain; 49 Biomolecular Engineering Department, University of California, Santa Cruz, Santa Cruz, CA 95064 USA; 50 Department of Internal Medicine, Stanford University, Stanford, CA 94305 USA; 51 Massachusetts General Hospital, Boston, MA 02114 USA; 52 Center for Precision Health, School of Biomedical Informatics, University of Texas Health Science Center, Houston, TX 77030 USA; 53 The Donnelly Centre, University of Toronto, Toronto, ON M5S 3E1 Canada; 54 Health Data Science Unit, University Clinics, Heidelberg, 69120 Germany; 55 Institute of Pharmacy and Molecular Biotechnology and BioQuant, Heidelberg University, Heidelberg, 69120 Germany; 56 Massachusetts General Hospital Center for Cancer Research, Charlestown, MA 02129 USA; 57 Simon Fraser University, Burnaby, BC V5A 1S6 Canada; 58 Department of Medical Biophysics, University of Toronto, Toronto, ON M5S 1A8 Canada; 59 Computational Biology Center, Memorial Sloan Kettering Cancer Center, New York, NY 10065 USA; 60 ETH Zurich, Department of Biology, Zürich, 8093 Switzerland; 61 ETH Zurich, Department of Computer Science, Zurich, 8092 Switzerland; 62 SIB Swiss Institute of Bioinformatics, Lausanne, 1015 Switzerland; 63 University Hospital Zurich, Zurich, 8091 Switzerland; 64 Clinical Bioinformatics, Swiss Institute of Bioinformatics, Geneva, 1202 Switzerland; 65 Institute for Pathology and Molecular Pathology, University Hospital Zurich, Zurich, 8091 Switzerland; 66 Institute of Molecular Life Sciences, University of Zurich, Zurich, 8057 Switzerland; 67 MIT Computer Science and Artificial Intelligence Laboratory, Massachusetts Institute of Technology, Cambridge, MA 02139 USA; 68 Englander Institute for Precision Medicine, Weill Cornell Medicine, New York, NY 10065 USA; 69 Meyer Cancer Center, Weill Cornell Medicine, New York, NY 10065 USA; 70 Research Core Center, National Cancer Centre Korea, Goyang-si, 410-769 South Korea; 71 Department of Health Sciences and Technology, Sungkyunkwan University School of Medicine, Seoul, 06351 South Korea; 72 Samsung Genome Institute, Samsung Medical Center, Seoul, South Korea; 73 Institute of Computer Science, Polish Academy of Sciences, Warsawa, 01-248 Poland; 74 Genome Biology Unit, European Molecular Biology Laboratory (EMBL), Heidelberg, 69117 Germany; 75 Institute of Biomedicine, Sahlgrenska Academy at University of Gothenburg, Gothenburg, Sweden; 76 ETH Zurich, Department of Biology, Wolfgang-Pauli-Strasse 27, 8093 Zürich, Switzerland; 77 Harvard University, Cambridge, MA 02138 USA; 78 Memorial Sloan Kettering Cancer Center, New York, NY 10065 USA; 79 Department of Molecular Biophysics and Biochemistry, New Haven, CT 06520 USA; 80 Yale University, New Haven, CT 06520 USA; 81 Department of Information Technology, Ghent University, Ghent, B-9000 Belgium; 82 Department of Plant Biotechnology and Bioinformatics, Ghent University, Ghent, B-9000 Belgium; 83 Yale School of Medicine, Yale University, New Haven, CT 06520 USA; 84 Division of Hematology-Oncology, Samsung Medical Center, Sungkyunkwan University School of Medicine, Seoul, 06351 South Korea; 85 Samsung Advanced Institute for Health Sciences and Technology, Sungkyunkwan University School of Medicine, Seoul, 06351 South Korea; 86 Cheonan Industry-Academic Collaboration Foundation, Sangmyung University, Cheonan, 31066 South Korea; 87 Spanish National Cancer Research Centre, Madrid, 28029 Spain; 88 Bern Center for Precision Medicine, University Hospital of Bern, University of Bern, Bern, 3008 Switzerland; 89 Englander Institute for Precision Medicine, Weill Cornell Medicine and NewYork Presbyterian Hospital, New York, NY 10021 USA; 90 Pathology and Laboratory, Weill Cornell Medical College, New York, NY 10021 USA; 91 Vall d’Hebron Institute of Oncology: VHIO, Barcelona, 08035 Spain; 92 National Centre for Biological Sciences, Tata Institute of Fundamental Research, Bangalore, 560065 India; 93 Indiana University, Bloomington, IN 47405 USA; 94 Vancouver Prostate Centre, Vancouver, BC V6H 3Z6 Canada; 95 cBio Center, Dana-Farber Cancer Institute, Harvard Medical School, Boston, MA 02115 USA; 96 Department of Cell Biology, Harvard Medical School, Boston, MA 02115 USA; 97 Department of Cancer Biology, Dana-Farber Cancer Institute, Boston, MA 02215 USA; 98 Peter MacCallum Cancer Centre and University of Melbourne, Melbourne, VIC 3000 Australia; 99 Finsen Laboratory and Biotech Research & Innovation Centre (BRIC), University of Copenhagen, Copenhagen, 2200 Denmark; 100 Department of Genetics, Stanford University School of Medicine, Stanford, CA 94305 USA; 101 CREST, Japan Science and Technology Agency, Tokyo, 113-0033 Japan; 102 Department of Medical Science Mathematics, Medical Research Institute, Tokyo Medical and Dental University, Bunkyo-ku, Tokyo, 113-8510 Japan; 103 Laboratory for Medical Science Mathematics, Department of Biological Sciences, Graduate School of Science, The University of Tokyo, Bunkyo-ku, Tokyo, 113-0033 Japan; 104 Department of Oncology-Pathology, Science for Life Laboratory, Karolinska Institute, Stockholm, Sweden; 105 Department of Gene Technology, Tallinn University of Technology, Tallinn, 12616 Estonia; 106 Genetics & Genome Biology Program, SickKids Research Institute, The Hospital for Sick Children, Toronto, ON M5G 1X8 Canada; 107 Institució Catalana de Recerca i Estudis Avançats (ICREA), Barcelona, 08010 Spain; 108 Department of Clinical and Molecular Medicine, Faculty of Medicine and Health Sciences, Norwegian University of Science and Technology, Trondheim, 7030 Norway; 109 Department of Information Technology, Ghent University, Interuniversitair Micro-Electronica Centrum (IMEC), Ghent, B-9000 Belgium; 110 Science for Life Laboratory, Department of Immunology, Genetics and Pathology, Uppsala University, Uppsala, SE-75108 Sweden; 111 School of Computer Science and Technology, Xi’an Jiaotong University, Xi’an, 710048 China; 112 School of Electronic and Information Engineering, Xi’an Jiaotong University, Xi’an, 710048 China; 113 The McDonnell Genome Institute at Washington University, St Louis, MO 63108 USA; 114 Department of Urology, Charité Universitätsmedizin Berlin, Berlin, 10117 Germany; 115 Department of Molecular and Human Genetics, Baylor College of Medicine, Houston, TX 77030 USA; 116 Human Genome Sequencing Center, Baylor College of Medicine, Houston, TX 77030 USA; 117 Oregon Health & Sciences University, Portland, OR 97239 USA; 118 Department of Medicine and Therapeutics, The Chinese University of Hong Kong, Shatin, NT, Hong Kong, China; 119 Second Military Medical University, Shanghai, 200433 China; 120 The University of Texas Health Science Center at Houston, Houston, TX 77030 USA; 121 Department of Biomedical Informatics, College of Medicine, The Ohio State University, Columbus, OH 43210 USA; 122 The Ohio State University Comprehensive Cancer Center (OSUCCC – James), Columbus, OH 43210 USA; 123 School of Biomedical Informatics, The University of Texas Health Science Center at Houston, Houston, TX 77030 USA; 124 Department of Biochemistry and Molecular Genetics, Feinberg School of Medicine, Northwestern University, Chicago, IL 60637 USA; 125 Institute of Molecular Life Sciences and Swiss Institute of Bioinformatics, University of Zurich, Zurich, 8057 Switzerland; 200 Applied Tumor Genomics Research Program, Research Programs Unit, University of Helsinki, Helsinki, Finland; 201 Wellcome Sanger Institute, Wellcome Genome Campus, Hinxton, UK; 202 Memorial Sloan Kettering Cancer Center, New York, NY USA; 203 Genome Science Division, Research Center for Advanced Science and Technology, University of Tokyo, Tokyo, Japan; 204 Department of Surgery, University of Chicago, Chicago, IL USA; 205 Department of Surgery, Division of Hepatobiliary and Pancreatic Surgery, School of Medicine, Keimyung University Dongsan Medical Center, Daegu, South Korea; 206 Department of Oncology, Gil Medical Center, Gachon University, Incheon, South Korea; 207 Hiroshima University, Hiroshima, Japan; 208 Department of Bioinformatics and Computational Biology, The University of Texas MD Anderson Cancer Center, Houston, TX USA; 209 University of Texas MD Anderson Cancer Center, Houston, TX USA; 210 King Faisal Specialist Hospital and Research Centre, Al Maather, Riyadh, Saudi Arabia; 211 Bioinformatics Unit, Spanish National Cancer Research Centre (CNIO), Madrid, Spain; 212 Bioinformatics Core Facility, University Medical Center Hamburg, Hamburg, Germany; 213 Heinrich Pette Institute, Leibniz Institute for Experimental Virology, Hamburg, Germany; 214 Ontario Tumour Bank, Ontario Institute for Cancer Research, Toronto, ON Canada; 215 Department of Pathology, The University of Texas MD Anderson Cancer Center, Houston, TX USA; 216 Laboratory of Pathology, Center for Cancer Research, National Cancer Institute, Bethesda, MD USA; 217 Department of Cellular and Molecular Medicine and Department of Bioengineering, University of California San Diego, La Jolla, CA USA; 218 UC San Diego Moores Cancer Center, San Diego, CA USA; 219 Canada’s Michael Smith Genome Sciences Centre, BC Cancer, Vancouver, BC Canada; 220 Sir Peter MacCallum Department of Oncology, Peter MacCallum Cancer Centre, University of Melbourne, Melbourne, VIC Australia; 221 Centre for Research in Molecular Medicine and Chronic Diseases (CiMUS), Universidade de Santiago de Compostela, Santiago de Compostela, Spain; 222 Department of Zoology, Genetics and Physical Anthropology, (CiMUS), Universidade de Santiago de Compostela, Santiago de Compostela, Spain; 223 The Biomedical Research Centre (CINBIO), Universidade de Vigo, Vigo, Spain; 224 Royal National Orthopaedic Hospital - Bolsover, London, UK; 225 Department of Genomic Medicine, The University of Texas MD Anderson Cancer Center, Houston, TX USA; 226 Quantitative and Computational Biosciences Graduate Program, Baylor College of Medicine, Houston, TX USA; 227 The Jackson Laboratory for Genomic Medicine, Farmington, CT USA; 228 Genome Informatics Program, Ontario Institute for Cancer Research, Toronto, ON Canada; 229 Institute of Human Genetics, Christian-Albrechts-University, Kiel, Germany; 230 Institute of Human Genetics, Ulm University and Ulm University Medical Center, Ulm, Germany; 231 Queensland Centre for Medical Genomics, Institute for Molecular Bioscience, University of Queensland, St. Lucia, Brisbane, QLD Australia; 232 Salford Royal NHS Foundation Trust, Salford, UK; 233 Department of Surgery, Pancreas Institute, University and Hospital Trust of Verona, Verona, Italy; 234 Molecular and Medical Genetics, OHSU Knight Cancer Institute, Oregon Health and Science University, Portland, OR USA; 235 Department of Molecular Oncology, BC Cancer Research Centre, Vancouver, BC Canada; 236 The McDonnell Genome Institute at Washington University, St. Louis, MO USA; 237 University College London, London, UK; 238 Division of Cancer Genomics, National Cancer Center Research Institute, National Cancer Center, Tokyo, Japan; 239 DLR Project Management Agency, Bonn, Germany; 240 Tokyo Women’s Medical University, Tokyo, Japan; 241 Center for Molecular Oncology, Memorial Sloan Kettering Cancer Center, New York, NY USA; 242 Los Alamos National Laboratory, Los Alamos, NM USA; 243 Department of Pathology, University Health Network, Toronto General Hospital, Toronto, ON Canada; 244 Nottingham University Hospitals NHS Trust, Nottingham, UK; 245 Epigenomics and Cancer Risk Factors, German Cancer Research Center (DKFZ), Heidelberg, Germany; 246 Computational Biology Program, Ontario Institute for Cancer Research, Toronto, ON Canada; 247 Department of Molecular Genetics, University of Toronto, Toronto, ON Canada; 248 Vector Institute, Toronto, ON Canada; 249 Hematopathology Section, Institute of Pathology, Christian-Albrechts-University, Kiel, Germany; 250 Department of Pathology and Laboratory Medicine, School of Medicine, University of North Carolina at Chapel Hill, Chapel Hill, NC USA; 251 Department of Cancer Genetics, Institute for Cancer Research, Oslo University Hospital, The Norwegian Radium Hospital, Oslo, Norway; 252 Pathology, Hospital Clinic, Institut d’Investigacions Biomèdiques August Pi i Sunyer (IDIBAPS), University of Barcelona, Barcelona, Spain; 253 Department of Veterinary Medicine, Transmissible Cancer Group, University of Cambridge, Cambridge, UK; 254 Alvin J. Siteman Cancer Center, Washington University School of Medicine, St. Louis, MO USA; 255 Wolfson Wohl Cancer Research Centre, Institute of Cancer Sciences, University of Glasgow, Glasgow, UK; 256 Lineberger Comprehensive Cancer Center, University of North Carolina at Chapel Hill, Chapel Hill, NC USA; 257 Broad Institute of MIT and Harvard, Cambridge, MA USA; 258 Dana-Farber/Boston Children’s Cancer and Blood Disorders Center, Boston, MA USA; 259 Department of Pediatrics, Harvard Medical School, Boston, MA USA; 260 Leeds Institute of Medical Research @ St. James’s, University of Leeds, St. James’s University Hospital, Leeds, UK; 261 Department of Pathology and Diagnostics, University and Hospital Trust of Verona, Verona, Italy; 262 Department of Surgery, Princess Alexandra Hospital, Brisbane, QLD Australia; 263 Surgical Oncology Group, Diamantina Institute, University of Queensland, Brisbane, QLD Australia; 264 Department of Population and Quantitative Health Sciences, Case Western Reserve University School of Medicine, Cleveland, OH USA; 265 Research Health Analytics and Informatics, University Hospitals Cleveland Medical Center, Cleveland, OH USA; 266 Gloucester Royal Hospital, Gloucester, UK; 267 European Molecular Biology Laboratory, European Bioinformatics Institute (EMBL-EBI), Cambridge, UK; 268 Diagnostic Development, Ontario Institute for Cancer Research, Toronto, ON Canada; 269 Barcelona Supercomputing Center (BSC), Barcelona, Spain; 270 Arnie Charbonneau Cancer Institute, University of Calgary, Calgary, AB Canada; 271 Departments of Surgery and Oncology, University of Calgary, Calgary, AB Canada; 272 Department of Pathology, Oslo University Hospital, The Norwegian Radium Hospital, Oslo, Norway; 273 PanCuRx Translational Research Initiative, Ontario Institute for Cancer Research, Toronto, ON Canada; 274 Department of Oncology, Sidney Kimmel Comprehensive Cancer Center at Johns Hopkins University School of Medicine, Baltimore, MD USA; 275 University Hospital Southampton NHS Foundation Trust, Southampton, UK; 276 Royal Stoke University Hospital, Stoke-on-Trent, UK; 277 Genome Sequence Informatics, Ontario Institute for Cancer Research, Toronto, ON Canada; 278 Human Longevity Inc, San Diego, CA USA; 279 Olivia Newton-John Cancer Research Institute, La Trobe University, Heidelberg, VIC Australia; 280 Computer Network Information Center, Chinese Academy of Sciences, Beijing, China; 281 Genome Canada, Ottawa, ON Canada; 282 CNAG-CRG, Centre for Genomic Regulation (CRG), Barcelona Institute of Science and Technology (BIST), Barcelona, Spain; 283 Universitat Pompeu Fabra (UPF), Barcelona, Spain; 284 Buck Institute for Research on Aging, Novato, CA USA; 285 Duke University Medical Center, Durham, NC USA; 286 Department of Human Genetics, Hannover Medical School, Hannover, Germany; 287 Center for Bioinformatics and Functional Genomics, Cedars-Sinai Medical Center, Los Angeles, CA USA; 288 Department of Biomedical Sciences, Cedars-Sinai Medical Center, Los Angeles, CA USA; 289 The Hebrew University Faculty of Medicine, Jerusalem, Israel; 290 Barts Cancer Institute, Barts and the London School of Medicine and Dentistry, Queen Mary University of London, London, UK; 291 Department of Computer Science, Bioinformatics Group, University of Leipzig, Leipzig, Germany; 292 Interdisciplinary Center for Bioinformatics, University of Leipzig, Leipzig, Germany; 293 Transcriptome Bioinformatics, LIFE Research Center for Civilization Diseases, University of Leipzig, Leipzig, Germany; 294 Department of Medical Oncology, Dana-Farber Cancer Institute, Boston, MA USA; 295 Department of Cancer Biology, Dana-Farber Cancer Institute, Boston, MA USA; 296 Harvard Medical School, Boston, MA USA; 297 USC Norris Comprehensive Cancer Center, University of Southern California, Los Angeles, CA USA; 298 Department of Diagnostics and Public Health, University and Hospital Trust of Verona, Verona, Italy; 299 Department of Mathematics, Aarhus University, Aarhus, Denmark; 300 Department of Molecular Medicine (MOMA), Aarhus University Hospital, Aarhus N, Denmark; 301 Instituto Carlos Slim de la Salud, Mexico City, Mexico; 302 Department of Medical Biophysics, University of Toronto, Toronto, ON Canada; 303 Cancer Division, Garvan Institute of Medical Research, Kinghorn Cancer Centre, University of New South Wales (UNSW Sydney), Sydney, NSW Australia; 304 South Western Sydney Clinical School, Faculty of Medicine, University of New South Wales (UNSW Sydney), Liverpool, NSW Australia; 305 West of Scotland Pancreatic Unit, Glasgow Royal Infirmary, Glasgow, UK; 306 Center for Digital Health, Berlin Institute of Health and Charitè - Universitätsmedizin Berlin, Berlin, Germany; 307 Heidelberg Center for Personalized Oncology (DKFZ-HIPO), German Cancer Research Center (DKFZ), Heidelberg, Germany; 308 The Preston Robert Tisch Brain Tumor Center, Duke University Medical Center, Durham, NC USA; 309 Massachusetts General Hospital, Boston, MA USA; 310 National Institute of Biomedical Genomics, Kalyani, West Bengal India; 311 Institute of Clinical Medicine and Institute of Oral Biology, University of Oslo, Oslo, Norway; 312 University of North Carolina at Chapel Hill, Chapel Hill, NC USA; 313 ARC-Net Centre for Applied Research on Cancer, University and Hospital Trust of Verona, Verona, Italy; 314 The Institute of Cancer Research, London, UK; 315 Centre for Computational Biology, Duke-NUS Medical School, Singapore, Singapore; 316 Programme in Cancer and Stem Cell Biology, Duke-NUS Medical School, Singapore, Singapore; 317 Division of Oncology and Pathology, Department of Clinical Sciences Lund, Lund University, Lund, Sweden; 318 Department of Pediatric Oncology, Hematology and Clinical Immunology, Heinrich-Heine-University, Düsseldorf, Germany; 319 Laboratory for Medical Science Mathematics, RIKEN Center for Integrative Medical Sciences, Yokohama, Japan; 320 RIKEN Center for Integrative Medical Sciences, Yokohama, Japan; 321 Department of Internal Medicine/Hematology, Friedrich-Ebert-Hospital, Neumünster, Germany; 322 Departments of Dermatology and Pathology, Yale University, New Haven, CT USA; 323 Centre for Genomic Regulation (CRG), The Barcelona Institute of Science and Technology, Barcelona, Spain; 324 Radcliffe Department of Medicine, University of Oxford, Oxford, UK; 325 Canadian Center for Computational Genomics, McGill University, Montreal, QC Canada; 326 Department of Human Genetics, McGill University, Montreal, QC Canada; 327 Department of Human Genetics, University of California Los Angeles, Los Angeles, CA USA; 328 Department of Pharmacology, University of Toronto, Toronto, ON Canada; 329 Faculty of Medicine and Health Technology, Tampere University and Tays Cancer Center, Tampere University Hospital, Tampere, Finland; 330 Haematology, Leeds Teaching Hospitals NHS Trust, Leeds, UK; 331 Translational Research and Innovation, Centre Léon Bérard, Lyon, France; 332 Fox Chase Cancer Center, Philadelphia, PA USA; 333 International Agency for Research on Cancer, World Health Organization, Lyon, France; 334 Earlham Institute, Norwich, UK; 335 Norwich Medical School, University of East Anglia, Norwich, UK; 336 Department of Molecular Biology, Faculty of Science, Radboud Institute for Molecular Life Sciences, Radboud University, Nijmegen, HB The Netherlands; 337 CRUK Manchester Institute and Centre, Manchester, UK; 338 Department of Radiation Oncology, University of Toronto, Toronto, ON Canada; 339 Division of Cancer Sciences, Manchester Cancer Research Centre, University of Manchester, Manchester, UK; 340 Radiation Medicine Program, Princess Margaret Cancer Centre, Toronto, ON Canada; 341 Department of Pathology, Brigham and Women’s Hospital, Harvard Medical School, Boston, MA USA; 342 Department of Surgery, Division of Thoracic Surgery, The Johns Hopkins University School of Medicine, Baltimore, MD USA; 343 Division of Molecular Pathology, The Netherlands Cancer Institute, Oncode Institute, Amsterdam, CX The Netherlands; 344 Department of Biomolecular Engineering, University of California Santa Cruz, Santa Cruz, CA USA; 345 UC Santa Cruz Genomics Institute, University of California Santa Cruz, Santa Cruz, CA USA; 346 Division of Applied Bioinformatics, German Cancer Research Center (DKFZ), Heidelberg, Germany; 347 German Cancer Consortium (DKTK), German Cancer Research Center (DKFZ), Heidelberg, Germany; 348 National Center for Tumor Diseases (NCT) Heidelberg, Heidelberg, Germany; 349 Center for Biological Sequence Analysis, Department of Bio and Health Informatics, Technical University of Denmark, Lyngby, Denmark; 350 Novo Nordisk Foundation Center for Protein Research, University of Copenhagen, Copenhagen, Denmark; 351 Institute for Molecular Bioscience, University of Queensland, St. Lucia, Brisbane, QLD Australia; 352 Biomedical Engineering, Oregon Health and Science University, Portland, OR USA; 353 Division of Theoretical Bioinformatics, German Cancer Research Center (DKFZ), Heidelberg, Germany; 354 Institute of Pharmacy and Molecular Biotechnology and BioQuant, Heidelberg University, Heidelberg, Germany; 355 Federal Ministry of Education and Research, Berlin, Germany; 356 Melanoma Institute Australia, University of Sydney, Sydney, NSW Australia; 357 Pediatric Hematology and Oncology, University Hospital Muenster, Muenster, Germany; 358 Department of Pathology, Johns Hopkins University School of Medicine, Baltimore, MD USA; 359 McKusick-Nathans Institute of Genetic Medicine, Sidney Kimmel Comprehensive Cancer Center at Johns Hopkins University School of Medicine, Baltimore, MD USA; 360 Foundation Medicine, Inc, Cambridge, MA USA; 361 Department of Biomedical Data Science, Stanford University School of Medicine, Stanford, CA USA; 362 Department of Genetics, Stanford University School of Medicine, Stanford, CA USA; 363 Bakar Computational Health Sciences Institute and Department of Pediatrics, University of California, San Francisco, CA USA; 364 Institute of Clinical Medicine, Faculty of Medicine, University of Oslo, Oslo, Norway; 365 National Cancer Institute, National Institutes of Health, Bethesda, MD USA; 366 Royal Marsden NHS Foundation Trust, London and Sutton, UK; 367 Genome Biology Unit, European Molecular Biology Laboratory (EMBL), Heidelberg, Germany; 368 Department of Oncology, University of Cambridge, Cambridge, UK; 369 Li Ka Shing Centre, Cancer Research UK Cambridge Institute, University of Cambridge, Cambridge, UK; 370 Institut Gustave Roussy, Villejuif, France; 371 Cambridge University Hospitals NHS Foundation Trust, Cambridge, UK; 372 Department of Haematology, University of Cambridge, Cambridge, UK; 373 Anatomia Patológica, Hospital Clinic, Institut d’Investigacions Biomèdiques August Pi i Sunyer (IDIBAPS), University of Barcelona, Barcelona, Spain; 374 Spanish Ministry of Science and Innovation, Madrid, Spain; 375 University of Michigan Comprehensive Cancer Center, Ann Arbor, MI USA; 376 Department for BioMedical Research, University of Bern, Bern, Switzerland; 377 Department of Medical Oncology, Inselspital, University Hospital and University of Bern, Bern, Switzerland; 378 Graduate School for Cellular and Biomedical Sciences, University of Bern, Bern, Switzerland; 379 University of Pavia, Pavia, Italy; 380 University of Alabama at Birmingham, Birmingham, AL USA; 381 UHN Program in BioSpecimen Sciences, Toronto General Hospital, Toronto, ON Canada; 382 Department of Urology, Icahn School of Medicine at Mount Sinai, New York, NY USA; 383 Centre for Law and Genetics, University of Tasmania, Sandy Bay Campus, Hobart, TAS Australia; 384 Faculty of Biosciences, Heidelberg University, Heidelberg, Germany; 385 Department of Biochemistry, Microbiology and Immunology, Faculty of Medicine, University of Ottawa, Ottawa, ON Canada; 386 Division of Anatomic Pathology, Mayo Clinic, Rochester, MN USA; 387 Division of Cancer Epidemiology and Genetics, National Cancer Institute, National Institutes of Health, Bethesda, MD USA; 388 Illawarra Shoalhaven Local Health District L3 Illawarra Cancer Care Centre, Wollongong Hospital, Wollongong, NSW Australia; 389 BioForA, French National Institute for Agriculture, Food, and Environment (INRAE), ONF, Orléans, France; 390 Department of Biostatistics, Bloomberg School of Public Health, Johns Hopkins University, Baltimore, MD USA; 391 University of California San Diego, San Diego, CA USA; 392 Division of Experimental Pathology, Mayo Clinic, Rochester, MN USA; 393 Centre for Cancer Research, The Westmead Institute for Medical Research, University of Sydney, Sydney, NSW Australia; 394 Department of Gynaecological Oncology, Westmead Hospital, Sydney, NSW Australia; 395 PDXen Biosystems Inc, Seoul, South Korea; 396 Korea Advanced Institute of Science and Technology, Daejeon, South Korea; 397 Electronics and Telecommunications Research Institute, Daejeon, South Korea; 398 Institut National du Cancer (INCA), Boulogne-Billancourt, France; 399 Department of Genetics, Informatics Institute, University of Alabama at Birmingham, Birmingham, AL USA; 400 Division of Medical Oncology, National Cancer Centre, Singapore, Singapore; 401 Medical Oncology, University and Hospital Trust of Verona, Verona, Italy; 402 Department of Pediatrics, University Hospital Schleswig-Holstein, Kiel, Germany; 403 Hepatobiliary/Pancreatic Surgical Oncology Program, University Health Network, Toronto, ON Canada; 404 School of Biological Sciences, University of Auckland, Auckland, New Zealand; 405 Department of Surgery, University of Melbourne, Parkville, VIC Australia; 406 The Murdoch Children’s Research Institute, Royal Children’s Hospital, Parkville, VIC Australia; 407 Walter and Eliza Hall Institute, Parkville, VIC Australia; 408 Vancouver Prostate Centre, Vancouver, Canada; 409 Lunenfeld-Tanenbaum Research Institute, Mount Sinai Hospital, Toronto, ON Canada; 410 University of East Anglia, Norwich, UK; 411 Norfolk and Norwich University Hospital NHS Trust, Norwich, UK; 412 Victorian Institute of Forensic Medicine, Southbank, VIC Australia; 413 Department of Biomedical Informatics, Harvard Medical School, Boston, MA USA; 414 Department of Chemistry, Centre for Molecular Science Informatics, University of Cambridge, Cambridge, UK; 415 Ludwig Center at Harvard Medical School, Boston, MA USA; 416 Human Genome Sequencing Center, Baylor College of Medicine, Houston, TX USA; 417 Peter MacCallum Cancer Centre, University of Melbourne, Melbourne, VIC Australia; 418 Physics Division, Optimization and Systems Biology Lab, Massachusetts General Hospital, Boston, MA USA; 419 Department of Medicine, Baylor College of Medicine, Houston, TX USA; 420 University of Cologne, Cologne, Germany; 421 International Genomics Consortium, Phoenix, AZ USA; 422 Genomics Research Program, Ontario Institute for Cancer Research, Toronto, ON Canada; 423 Barking Havering and Redbridge University Hospitals NHS Trust, Romford, UK; 424 Children’s Hospital at Westmead, University of Sydney, Sydney, NSW Australia; 425 Department of Medicine, Section of Endocrinology, University and Hospital Trust of Verona, Verona, Italy; 426 Computational Biology Center, Memorial Sloan Kettering Cancer Center, New York, NY USA; 427 Department of Biology, ETH Zurich, Zürich, Switzerland; 428 Department of Computer Science, ETH Zurich, Zurich, Switzerland; 429 SIB Swiss Institute of Bioinformatics, Lausanne, Switzerland; 430 Weill Cornell Medical College, New York, NY USA; 431 Academic Department of Medical Genetics, University of Cambridge, Addenbrooke’s Hospital, Cambridge, UK; 432 MRC Cancer Unit, University of Cambridge, Cambridge, UK; 433 Departments of Pediatrics and Genetics, University of North Carolina at Chapel Hill, Chapel Hill, NC USA; 434 Seven Bridges Genomics, Charlestown, MA USA; 435 Annai Systems, Inc, Carlsbad, CA USA; 436 Department of Pathology, General Hospital of Treviso, Department of Medicine, University of Padua, Treviso, Italy; 437 Department of Computational Biology, University of Lausanne, Lausanne, Switzerland; 438 Department of Genetic Medicine and Development, University of Geneva Medical School, Geneva, CH Switzerland; 439 Swiss Institute of Bioinformatics, University of Geneva, Geneva, CH Switzerland; 440 The Francis Crick Institute, London, UK; 441 University of Leuven, Leuven, Belgium; 442 Institute of Medical Genetics and Applied Genomics, University of Tübingen, Tübingen, Germany; 443 Computational and Systems Biology, Genome Institute of Singapore, Singapore, Singapore; 444 School of Computing, National University of Singapore, Singapore, Singapore; 445 Big Data Institute, Li Ka Shing Centre, University of Oxford, Oxford, UK; 446 Biomedical Data Science Laboratory, Francis Crick Institute, London, UK; 447 Bioinformatics Group, Department of Computer Science, University College London, London, UK; 448 The Edward S. Rogers Sr. Department of Electrical and Computer Engineering, University of Toronto, Toronto, ON Canada; 449 Breast Cancer Translational Research Laboratory JC Heuson, Institut Jules Bordet, Brussels, Belgium; 450 Department of Oncology, Laboratory for Translational Breast Cancer Research, KU Leuven, Leuven, Belgium; 451 Institute for Research in Biomedicine (IRB Barcelona), The Barcelona Institute of Science and Technology, Barcelona, Spain; 452 Research Program on Biomedical Informatics, Universitat Pompeu Fabra, Barcelona, Spain; 453 Division of Medical Oncology, Princess Margaret Cancer Centre, Toronto, ON Canada; 454 Department of Physiology and Biophysics, Weill Cornell Medicine, New York, NY USA; 455 Institute for Computational Biomedicine, Weill Cornell Medicine, New York, NY USA; 456 Department of Pathology, UPMC Shadyside, Pittsburgh, PA USA; 457 Independent Consultant, Wellesley, USA; 458 Department of Cell and Molecular Biology, Science for Life Laboratory, Uppsala University, Uppsala, Sweden; 459 Department of Medicine and Department of Genetics, Washington University School of Medicine, St. Louis, St. Louis, MO USA; 460 Hefei University of Technology, Anhui, China; 461 Translational Cancer Research Unit, GZA Hospitals St.-Augustinus, Center for Oncological Research, Faculty of Medicine and Health Sciences, University of Antwerp, Antwerp, Belgium; 462 Simon Fraser University, Burnaby, BC Canada; 463 University of Pennsylvania, Philadelphia, PA USA; 464 Faculty of Science and Technology, University of Vic—Central University of Catalonia (UVic-UCC), Vic, Spain; 465 The Wellcome Trust, London, UK; 466 The Hospital for Sick Children, Toronto, ON Canada; 467 Department of Pathology, Queen Elizabeth University Hospital, Glasgow, UK; 468 Department of Genetics and Computational Biology, QIMR Berghofer Medical Research Institute, Brisbane, QLD Australia; 469 Department of Oncology, Centre for Cancer Genetic Epidemiology, University of Cambridge, Cambridge, UK; 470 Department of Public Health and Primary Care, Centre for Cancer Genetic Epidemiology, University of Cambridge, Cambridge, UK; 471 Prostate Cancer Canada, Toronto, ON Canada; 472 University of Cambridge, Cambridge, UK; 473 Department of Laboratory Medicine, Translational Cancer Research, Lund University Cancer Center at Medicon Village, Lund University, Lund, Sweden; 474 Heidelberg University, Heidelberg, Germany; 475 New BIH Digital Health Center, Berlin Institute of Health (BIH) and Charité - Universitätsmedizin Berlin, Berlin, Germany; 476 CIBER Epidemiología y Salud Pública (CIBERESP), Madrid, Spain; 477 Research Group on Statistics, Econometrics and Health (GRECS), UdG, Barcelona, Spain; 478 Quantitative Genomics Laboratories (qGenomics), Barcelona, Spain; 479 Icelandic Cancer Registry, Icelandic Cancer Society, Reykjavik, Iceland; 480 State Key Laboratory of Cancer Biology, and Xijing Hospital of Digestive Diseases, Fourth Military Medical University, Shaanxi, China; 481 Department of Medicine (DIMED), Surgical Pathology Unit, University of Padua, Padua, Italy; 482 Rigshospitalet, Copenhagen, Denmark; 483 Center for Cancer Genomics, National Cancer Institute, National Institutes of Health, Bethesda, MD USA; 484 Department of Biochemistry and Molecular Medicine, University of Montreal, Montreal, QC Canada; 485 Australian Institute of Tropical Health and Medicine, James Cook University, Douglas, QLD Australia; 486 Department of Neuro-Oncology, Istituto Neurologico Besta, Milano, Italy; 487 Bioplatforms Australia, North Ryde, NSW Australia; 488 Department of Pathology (Research), University College London Cancer Institute, London, UK; 489 Department of Surgical Oncology, Princess Margaret Cancer Centre, Toronto, ON Canada; 490 Department of Medical Oncology, Josephine Nefkens Institute and Cancer Genomics Centre, Erasmus Medical Center, Rotterdam, CN The Netherlands; 491 The University of Queensland Thoracic Research Centre, The Prince Charles Hospital, Brisbane, QLD Australia; 492 CIBIO/InBIO - Research Center in Biodiversity and Genetic Resources, Universidade do Porto, Vairão, Portugal; 493 HCA Laboratories, London, UK; 494 University of Liverpool, Liverpool, UK; 495 The Azrieli Faculty of Medicine, Bar-Ilan University, Safed, Israel; 496 Department of Neurosurgery, University of Florida, Gainesville, FL USA; 497 Department of Pathology, Graduate School of Medicine, University of Tokyo, Tokyo, Japan; 498 University of Milano Bicocca, Monza, Italy; 499 BGI-Shenzhen, Shenzhen, China; 500 Department of Pathology, Oslo University Hospital Ulleval, Oslo, Norway; 501 Center for Biomedical Informatics, Harvard Medical School, Boston, MA USA; 502 Department Biochemistry and Molecular Biomedicine, University of Barcelona, Barcelona, Spain; 503 Office of Cancer Genomics, National Cancer Institute, National Institutes of Health, Bethesda, MD USA; 504 Cancer Epigenomics, German Cancer Research Center (DKFZ), Heidelberg, Germany; 505 Department of Cancer Biology, The University of Texas MD Anderson Cancer Center, Houston, TX USA; 506 Department of Surgical Oncology, The University of Texas MD Anderson Cancer Center, Houston, TX USA; 507 Department of Computer Science, Yale University, New Haven, CT USA; 508 Department of Molecular Biophysics and Biochemistry, Yale University, New Haven, CT USA; 509 Program in Computational Biology and Bioinformatics, Yale University, New Haven, CT USA; 510 Center for Cancer Research, Massachusetts General Hospital, Boston, MA USA; 511 Department of Pathology, Massachusetts General Hospital, Boston, MA USA; 512 Department of Pathology, Memorial Sloan Kettering Cancer Center, New York, NY USA; 513 Division of Gastroenterology and Hepatology, Mayo Clinic, Rochester, MN USA; 514 University of Sydney, Sydney, NSW Australia; 515 University of Oxford, Oxford, UK; 516 Department of Surgery, Academic Urology Group, University of Cambridge, Cambridge, UK; 517 Department of Medicine II, University of Würzburg, Wuerzburg, Germany; 518 Sylvester Comprehensive Cancer Center, University of Miami, Miami, FL USA; 519 Institut Hospital del Mar d’Investigacions Mèdiques (IMIM), Barcelona, Spain; 520 Genome Integrity and Structural Biology Laboratory, National Institute of Environmental Health Sciences (NIEHS), Durham, NC USA; 521 St. Thomas’s Hospital, London, UK; 522 Osaka International Cancer Center, Osaka, Japan; 523 Department of Pathology, Skåne University Hospital, Lund University, Lund, Sweden; 524 Department of Medical Oncology, Beatson West of Scotland Cancer Centre, Glasgow, UK; 525 National Human Genome Research Institute, National Institutes of Health, Bethesda, MD USA; 526 Centre for Cancer Research, Victorian Comprehensive Cancer Centre, University of Melbourne, Melbourne, VIC Australia; 527 Department of Medicine, Section of Hematology/Oncology, University of Chicago, Chicago, IL USA; 528 German Center for Infection Research (DZIF), Partner Site Hamburg-Borstel-Lübeck-Riems, Hamburg, Germany; 529 Bioinformatics Research Centre (BiRC), Aarhus University, Aarhus, Denmark; 530 Department of Biotechnology, Ministry of Science and Technology, Government of India, New Delhi, Delhi India; 531 National Cancer Centre Singapore, Singapore, Singapore; 532 Brandeis University, Waltham, MA USA; 533 Department of Urologic Sciences, University of British Columbia, Vancouver, BC Canada; 534 Department of Internal Medicine, Stanford University, Stanford, CA USA; 535 The University of Texas Health Science Center at Houston, Houston, TX USA; 536 Imperial College NHS Trust, Imperial College, London, INY UK; 537 Senckenberg Institute of Pathology, University of Frankfurt Medical School, Frankfurt, Germany; 538 Department of Medicine, Division of Biomedical Informatics, UC San Diego School of Medicine, San Diego, CA USA; 539 Center for Precision Health, School of Biomedical Informatics, The University of Texas Health Science Center, Houston, TX USA; 540 Oxford Nanopore Technologies, New York, NY USA; 541 Institute of Medical Science, University of Tokyo, Tokyo, Japan; 542 Howard Hughes Medical Institute, University of California Santa Cruz, Santa Cruz, CA USA; 543 Wakayama Medical University, Wakayama, Japan; 544 Department of Internal Medicine, Division of Medical Oncology, Lineberger Comprehensive Cancer Center, University of North Carolina at Chapel Hill, Chapel Hill, NC USA; 545 University of Tennessee Health Science Center for Cancer Research, Memphis, TN USA; 546 Department of Histopathology, Salford Royal NHS Foundation Trust, Salford, UK; 547 Faculty of Biology, Medicine and Health, University of Manchester, Manchester, UK; 548 BIOPIC, ICG and College of Life Sciences, Peking University, Beijing, China; 549 Peking-Tsinghua Center for Life Sciences, Peking University, Beijing, China; 550 Children’s Hospital of Philadelphia, Philadelphia, PA USA; 551 Department of Bioinformatics and Computational Biology and Department of Systems Biology, The University of Texas MD Anderson Cancer Center, Houston, TX USA; 552 Karolinska Institute, Stockholm, Sweden; 553 The Donnelly Centre, University of Toronto, Toronto, ON Canada; 554 Department of Medical Genetics, College of Medicine, Hallym University, Chuncheon, South Korea; 555 Department of Experimental and Health Sciences, Institute of Evolutionary Biology (UPF-CSIC), Universitat Pompeu Fabra, Barcelona, Spain; 556 Health Data Science Unit, University Clinics, Heidelberg, Germany; 557 Massachusetts General Hospital Center for Cancer Research, Charlestown, MA USA; 558 Hokkaido University, Sapporo, Japan; 559 Department of Pathology and Clinical Laboratory, National Cancer Center Hospital, Tokyo, Japan; 560 Department of Genetics, University of North Carolina at Chapel Hill, Chapel Hill, NC USA; 561 Computational Biology, Leibniz Institute on Aging - Fritz Lipmann Institute (FLI), Jena, Germany; 562 University of Melbourne Centre for Cancer Research, Melbourne, VIC Australia; 563 University of Nebraska Medical Center, Omaha, NE USA; 564 Syntekabio Inc, Daejeon, South Korea; 565 Department of Pathology, Academic Medical Center, Amsterdam, AZ The Netherlands; 566 China National GeneBank-Shenzhen, Shenzhen, China; 567 Division of Molecular Genetics, German Cancer Research Center (DKFZ), Heidelberg, Germany; 568 Division of Life Science and Applied Genomics Center, Hong Kong University of Science and Technology, Clear Water Bay, Hong Kong, China; 569 Icahn School of Medicine at Mount Sinai, New York, NY USA; 570 Geneplus-Shenzhen, Shenzhen, China; 571 School of Computer Science and Technology, Xi’an Jiaotong University, Xi’an, China; 572 AbbVie, North Chicago, IL USA; 573 Institute of Pathology, Charité – University Medicine Berlin, Berlin, Germany; 574 Centre for Translational and Applied Genomics, British Columbia Cancer Agency, Vancouver, BC Canada; 575 Edinburgh Royal Infirmary, Edinburgh, UK; 576 Berlin Institute for Medical Systems Biology, Max Delbrück Center for Molecular Medicine, Berlin, Germany; 577 Department of Pediatric Immunology, Hematology and Oncology, University Hospital, Heidelberg, Germany; 578 German Cancer Research Center (DKFZ), Heidelberg, Germany; 579 Heidelberg Institute for Stem Cell Technology and Experimental Medicine (HI-STEM), Heidelberg, Germany; 580 Institute for Computational Biomedicine, Weill Cornell Medical College, New York, NY USA; 581 New York Genome Center, New York, NY USA; 582 Department of Urology, James Buchanan Brady Urological Institute, Johns Hopkins University School of Medicine, Baltimore, MD USA; 583 Department of Preventive Medicine, Graduate School of Medicine, The University of Tokyo, Tokyo, Japan; 584 Department of Molecular and Cellular Biology, Baylor College of Medicine, Houston, TX USA; 585 Department of Pathology and Immunology, Baylor College of Medicine, Houston, TX USA; 586 Michael E. DeBakey Veterans Affairs Medical Center, Houston, TX USA; 587 Technical University of Denmark, Lyngby, Denmark; 588 Department of Pathology, College of Medicine, Hanyang University, Seoul, South Korea; 589 Academic Unit of Surgery, School of Medicine, College of Medical, Veterinary and Life Sciences, University of Glasgow, Glasgow Royal Infirmary, Glasgow, UK; 590 Department of Pathology, Asan Medical Center, College of Medicine, Ulsan University, Songpa-gu, Seoul South Korea; 591 Science Writer, Garrett Park, MD USA; 592 International Cancer Genome Consortium (ICGC)/ICGC Accelerating Research in Genomic Oncology (ARGO) Secretariat, Ontario Institute for Cancer Research, Toronto, ON Canada; 593 University of Ljubljana, Ljubljana, Slovenia; 594 Department of Public Health Sciences, University of Chicago, Chicago, IL USA; 595 Research Institute, NorthShore University HealthSystem, Evanston, IL USA; 596 Department for Biomedical Research, University of Bern, Bern, Switzerland; 597 Centre of Genomics and Policy, McGill University and Génome Québec Innovation Centre, Montreal, QC Canada; 598 Carolina Center for Genome Sciences, University of North Carolina at Chapel Hill, Chapel Hill, NC USA; 599 Hopp Children’s Cancer Center (KiTZ), Heidelberg, Germany; 600 Pediatric Glioma Research Group, German Cancer Research Center (DKFZ), Heidelberg, Germany; 601 Cancer Research UK, London, UK; 602 Indivumed GmbH, Hamburg, Germany; 603 Genome Integration Data Center, Syntekabio, Inc, Daejeon, South Korea; 604 University Hospital Zurich, Zurich, Switzerland; 605 Clinical Bioinformatics, Swiss Institute of Bioinformatics, Geneva, Switzerland; 606 Institute for Pathology and Molecular Pathology, University Hospital Zurich, Zurich, Switzerland; 607 Institute of Molecular Life Sciences, University of Zurich, Zurich, Switzerland; 608 MRC Human Genetics Unit, MRC IGMM, University of Edinburgh, Edinburgh, UK; 609 Women’s Cancer Program at the Samuel Oschin Comprehensive Cancer Institute, Cedars-Sinai Medical Center, Los Angeles, CA USA; 610 Department of Biology, Bioinformatics Group, Division of Molecular Biology, Faculty of Science, University of Zagreb, Zagreb, Croatia; 611 Department for Internal Medicine II, University Hospital Schleswig-Holstein, Kiel, Germany; 612 Genetics and Molecular Pathology, SA Pathology, Adelaide, SA Australia; 613 Department of Gastric Surgery, National Cancer Center Hospital, Tokyo, Japan; 614 Department of Bioinformatics, Division of Cancer Genomics, National Cancer Center Research Institute, Tokyo, Japan; 615 A.A. Kharkevich Institute of Information Transmission Problems, Moscow, Russia; 616 Oncology and Immunology, Dmitry Rogachev National Research Center of Pediatric Hematology, Moscow, Russia; 617 Skolkovo Institute of Science and Technology, Moscow, Russia; 618 Department of Surgery, The George Washington University, School of Medicine and Health Science, Washington, DC USA; 619 Endocrine Oncology Branch, Center for Cancer Research, National Cancer Institute, National Institutes of Health, Bethesda, MD USA; 620 Melanoma Institute Australia, Macquarie University, Sydney, NSW Australia; 621 MIT Computer Science and Artificial Intelligence Laboratory, Massachusetts Institute of Technology, Cambridge, MA USA; 622 Tissue Pathology and Diagnostic Oncology, Royal Prince Alfred Hospital, Sydney, NSW Australia; 623 Cholangiocarcinoma Screening and Care Program and Liver Fluke and Cholangiocarcinoma Research Centre, Faculty of Medicine, Khon Kaen University, Khon Kaen, Thailand; 624 Controlled Department and Institution, New York, NY USA; 625 Englander Institute for Precision Medicine, Weill Cornell Medicine, New York, NY USA; 626 National Cancer Center, Gyeonggi, South Korea; 627 Department of Biochemistry, College of Medicine, Ewha Womans University, Seoul, South Korea; 628 Health Sciences Department of Biomedical Informatics, University of California San Diego, La Jolla, CA USA; 629 Research Core Center, National Cancer Centre Korea, Goyang-si, South Korea; 630 Department of Health Sciences and Technology, Sungkyunkwan University School of Medicine, Seoul, South Korea; 631 Samsung Genome Institute, Seoul, South Korea; 632 Breast Oncology Program, Dana-Farber/Brigham and Women’s Cancer Center, Boston, MA USA; 633 Department of Surgery, Memorial Sloan Kettering Cancer Center, New York, NY USA; 634 Division of Breast Surgery, Brigham and Women’s Hospital, Boston, MA USA; 635 Integrative Bioinformatics Support Group, National Institute of Environmental Health Sciences (NIEHS), Durham, NC USA; 636 Department of Clinical Science, University of Bergen, Bergen, Norway; 637 Center For Medical Innovation, Seoul National University Hospital, Seoul, South Korea; 638 Department of Internal Medicine, Seoul National University Hospital, Seoul, South Korea; 639 Institute of Computer Science, Polish Academy of Sciences, Warsawa, Poland; 640 Functional and Structural Genomics, German Cancer Research Center (DKFZ), Heidelberg, Germany; 641 Laboratory of Translational Genomics, Division of Cancer Epidemiology and Genetics, National Cancer Institute, , National Institutes of Health, Bethesda, MD USA; 642 Institute for Medical Informatics Statistics and Epidemiology, University of Leipzig, Leipzig, Germany; 643 Morgan Welch Inflammatory Breast Cancer Research Program and Clinic, The University of Texas MD Anderson Cancer Center, Houston, TX USA; 644 Department of Hematology and Oncology, Georg-Augusts-University of Göttingen, Göttingen, Germany; 645 Institute of Cell Biology (Cancer Research), University of Duisburg-Essen, Essen, Germany; 646 King’s College London and Guy’s and St. Thomas’ NHS Foundation Trust, London, UK; 647 Center for Epigenetics, Van Andel Research Institute, Grand Rapids, MI USA; 648 The University of Queensland Centre for Clinical Research, Royal Brisbane and Women’s Hospital, Herston, QLD Australia; 649 Department of Pediatric Oncology and Hematology, University of Cologne, Cologne, Germany; 650 University of Düsseldorf, Düsseldorf, Germany; 651 Department of Pathology, Institut Jules Bordet, Brussels, Belgium; 652 Institute of Biomedicine, Sahlgrenska Academy at University of Gothenburg, Gothenburg, Sweden; 653 Children’s Medical Research Institute, Sydney, NSW Australia; 654 ILSbio, LLC Biobank, Chestertown, MD USA; 655 Division of Genetics and Genomics, Boston Children’s Hospital, Harvard Medical School, Boston, MA USA; 656 Institute for Bioengineering and Biopharmaceutical Research (IBBR), Hanyang University, Seoul, South Korea; 657 Department of Statistics, University of California Santa Cruz, Santa Cruz, CA USA; 658 National Genotyping Center, Institute of Biomedical Sciences, Academia Sinica, Taipei, Taiwan; 659 Department of Vertebrate Genomics/Otto Warburg Laboratory Gene Regulation and Systems Biology of Cancer, Max Planck Institute for Molecular Genetics, Berlin, Germany; 660 McGill University and Genome Quebec Innovation Centre, Montreal, QC Canada; 661 biobyte solutions GmbH, Heidelberg, Germany; 662 Gynecologic Oncology, NYU Laura and Isaac Perlmutter Cancer Center, New York University, New York, NY USA; 663 Division of Oncology, Stem Cell Biology Section, Washington University School of Medicine, St. Louis, MO USA; 664 Department of Systems Biology, The University of Texas MD Anderson Cancer Center, Houston, TX USA; 665 Harvard University, Cambridge, MA USA; 666 Urologic Oncology Branch, Center for Cancer Research, National Cancer Institute, National Institutes of Health, Bethesda, MD USA; 667 University of Oslo, Oslo, Norway; 668 University of Toronto, Toronto, ON Canada; 669 Peking University, Beijing, China; 670 School of Life Sciences, Peking University, Beijing, China; 671 Leidos Biomedical Research, Inc, McLean, VA USA; 672 Hematology, Hospital Clinic, Institut d’Investigacions Biomèdiques August Pi i Sunyer (IDIBAPS), University of Barcelona, Barcelona, Spain; 673 Second Military Medical University, Shanghai, China; 674 Chinese Cancer Genome Consortium, Shenzhen, China; 675 Department of Medical Oncology, Beijing Hospital, Beijing, China; 676 Laboratory of Molecular Oncology, Key Laboratory of Carcinogenesis and Translational Research (Ministry of Education), Peking University Cancer Hospital and Institute, Beijing, China; 677 School of Medicine/School of Mathematics and Statistics, University of St. Andrews, St, Andrews, Fife UK; 678 Institute for Systems Biology, Seattle, WA USA; 679 Department of Biochemistry and Molecular Biology, Faculty of Medicine, University Institute of Oncology-IUOPA, Oviedo, Spain; 680 Institut Bergonié, Bordeaux, France; 681 Cancer Unit, MRC University of Cambridge, Cambridge, UK; 682 Department of Pathology and Laboratory Medicine, Center for Personalized Medicine, Children’s Hospital Los Angeles, Los Angeles, CA USA; 683 John Curtin School of Medical Research, Canberra, ACT Australia; 684 MVZ Department of Oncology, PraxisClinic am Johannisplatz, Leipzig, Germany; 685 Department of Information Technology, Ghent University, Ghent, Belgium; 686 Department of Plant Biotechnology and Bioinformatics, Ghent University, Ghent, Belgium; 687 Institute for Genomic Medicine, Nationwide Children’s Hospital, Columbus, OH USA; 688 Computational Biology Program, School of Medicine, Oregon Health and Science University, Portland, OR USA; 689 Department of Surgery, Duke University, Durham, NC USA; 690 Institució Catalana de Recerca i Estudis Avançats (ICREA), Barcelona, Spain; 691 Institut Català de Paleontologia Miquel Crusafont, Universitat Autònoma de Barcelona, Barcelona, Spain; 692 University of Glasgow, Glasgow, UK; 693 Institut d’Investigacions Biomèdiques August Pi i Sunyer (IDIBAPS), Barcelona, Spain; 694 Division of Oncology, Washington University School of Medicine, St. Louis, MO USA; 695 Department of Surgery and Cancer, Imperial College, London, INY UK; 696 Applications Department, Oxford Nanopore Technologies, Oxford, UK; 697 Department of Obstetrics, Gynecology and Reproductive Services, University of California San Francisco, San Francisco, CA USA; 698 Department of Biochemistry and Molecular Medicine, University California at Davis, Sacramento, CA USA; 699 STTARR Innovation Facility, Princess Margaret Cancer Centre, Toronto, ON Canada; 700 Discipline of Surgery, Western Sydney University, Penrith, NSW Australia; 701 Yale School of Medicine, Yale University, New Haven, CT USA; 702 Department of Genetics, Lineberger Comprehensive Cancer Center, University of North Carolina at Chapel Hill, Chapel Hill, NC USA; 703 Departments of Neurology and Neurosurgery, Henry Ford Hospital, Detroit, MI USA; 704 Precision Oncology, OHSU Knight Cancer Institute, Oregon Health and Science University, Portland, OR USA; 705 Institute of Pathology, University Medical Center Hamburg-Eppendorf, Hamburg, Germany; 706 Department of Health Sciences, Faculty of Medical Sciences, Kyushu University, Fukuoka, Japan; 707 Heidelberg Academy of Sciences and Humanities, Heidelberg, Germany; 708 Department of Clinical Pathology, University of Melbourne, Melbourne, VIC, Australia; 709 Department of Pathology, Roswell Park Cancer Institute, Buffalo, NY USA; 710 Department of Computer Science, University of Helsinki, Helsinki, Finland; 711 Institute of Biotechnology, University of Helsinki, Helsinki, Finland; 712 Organismal and Evolutionary Biology Research Programme, University of Helsinki, Helsinki, Finland; 713 Department of Obstetrics and Gynecology, Division of Gynecologic Oncology, Washington University School of Medicine, St. Louis, MO USA; 714 Penrose St. Francis Health Services, Colorado Springs, CO USA; 715 Institute of Pathology, Ulm University and University Hospital of Ulm, Ulm, Germany; 716 National Cancer Center, Tokyo, Japan; 717 Genome Institute of Singapore, Singapore, Singapore; 718 32Program in Computational Biology and Bioinformatics, Yale University, New Haven, CT USA; 719 German Cancer Aid, Bonn, Germany; 720 Programme in Cancer and Stem Cell Biology, Centre for Computational Biology, Duke-NUS Medical School, Singapore, Singapore; 721 The Chinese University of Hong Kong, Shatin, NT, Hong Kong China; 722 Fourth Military Medical University, Shaanxi, China; 723 The University of Cambridge School of Clinical Medicine, Cambridge, UK; 724 St. Jude Children’s Research Hospital, Memphis, TN USA; 725 University Health Network, Princess Margaret Cancer Centre, Toronto, ON Canada; 726 Center for Biomolecular Science and Engineering, University of California Santa Cruz, Santa Cruz, CA USA; 727 Department of Medicine, University of Chicago, Chicago, IL USA; 728 Department of Neurology, Mayo Clinic, Rochester, MN USA; 729 Cambridge Oesophagogastric Centre, Cambridge University Hospitals NHS Foundation Trust, Cambridge, UK; 730 Department of Computer Science, Carleton College, Northfield, MN USA; 731 Institute of Cancer Sciences, College of Medical Veterinary and Life Sciences, University of Glasgow, Glasgow, UK; 732 Department of Epidemiology, University of Alabama at Birmingham, Birmingham, AL USA; 733 HudsonAlpha Institute for Biotechnology, Huntsville, AL USA; 734 O’Neal Comprehensive Cancer Center, University of Alabama at Birmingham, Birmingham, AL USA; 735 Department of Pathology, Keio University School of Medicine, Tokyo, Japan; 736 Department of Hepatobiliary and Pancreatic Oncology, National Cancer Center Hospital, Tokyo, Japan; 737 Sage Bionetworks, Seattle, WA USA; 738 Lymphoma Genomic Translational Research Laboratory, National Cancer Centre, Singapore, Singapore; 739 Department of Clinical Pathology, Robert-Bosch-Hospital, Stuttgart, Germany; 740 Department of Cell and Systems Biology, University of Toronto, Toronto, ON Canada; 741 Department of Biosciences and Nutrition, Karolinska Institutet, Stockholm, Sweden; 742 Center for Liver Cancer, Research Institute and Hospital, National Cancer Center, Gyeonggi, South Korea; 743 Division of Hematology-Oncology, Samsung Medical Center, Sungkyunkwan University School of Medicine, Seoul, South Korea; 744 Samsung Advanced Institute for Health Sciences and Technology, Sungkyunkwan University School of Medicine, Seoul, South Korea; 745 Cheonan Industry-Academic Collaboration Foundation, Sangmyung University, Cheonan, South Korea; 746 NYU Langone Medical Center, New York, NY USA; 747 Department of Hematology and Medical Oncology, Cleveland Clinic, Cleveland, OH USA; 748 Department of Radiation Oncology, University of California San Francisco, San Francisco, CA USA; 749 Department of Health Sciences Research, Mayo Clinic, Rochester, MN USA; 750 Helen F. Graham Cancer Center at Christiana Care Health Systems, Newark, DE USA; 751 Heidelberg University Hospital, Heidelberg, Germany; 752 CSRA Incorporated, Fairfax, VA USA; 753 Research Department of Pathology, University College London Cancer Institute, London, UK; 754 Department of Research Oncology, Guy’s Hospital, King’s Health Partners AHSC, King’s College London School of Medicine, London, UK; 755 Faculty of Medicine and Health Sciences, Macquarie University, Sydney, NSW Australia; 756 University Hospital of Minjoz, INSERM UMR 1098, Besançon, France; 757 Spanish National Cancer Research Centre, Madrid, Spain; 758 Center of Digestive Diseases and Liver Transplantation, Fundeni Clinical Institute, Bucharest, Romania; 759 Cureline, Inc, South San Francisco, CA USA; 760 St. Luke’s Cancer Centre, Royal Surrey County Hospital NHS Foundation Trust, Guildford, UK; 761 Cambridge Breast Unit, Addenbrooke’s Hospital, Cambridge University Hospital NHS Foundation Trust and NIHR Cambridge Biomedical Research Centre, Cambridge, UK; 762 East of Scotland Breast Service, Ninewells Hospital, Aberdeen, UK; 763 Department of Genetics, Microbiology and Statistics, University of Barcelona, IRSJD, IBUB, Barcelona, Spain; 764 Department of Obstetrics and Gynecology, Medical College of Wisconsin, Milwaukee, WI USA; 765 Hematology and Medical Oncology, Winship Cancer Institute of Emory University, Atlanta, GA USA; 766 Department of Computer Science, Princeton University, Princeton, NJ USA; 767 Vanderbilt Ingram Cancer Center, Vanderbilt University, Nashville, TN USA; 768 Ohio State University College of Medicine and Arthur G. James Comprehensive Cancer Center, Columbus, OH USA; 769 Department of Surgery, Yokohama City University Graduate School of Medicine, Kanagawa, Japan; 770 Division of Chromatin Networks, German Cancer Research Center (DKFZ) and BioQuant, Heidelberg, Germany; 771 Research Computing Center, University of North Carolina at Chapel Hill, Chapel Hill, NC USA; 772 School of Molecular Biosciences and Center for Reproductive Biology, Washington State University, Pullman, WA USA; 773 Finsen Laboratory and Biotech Research and Innovation Centre (BRIC), University of Copenhagen, Copenhagen, Denmark; 774 Department of Laboratory Medicine and Pathobiology, University of Toronto, Toronto, ON Canada; 775 Department of Pathology, Human Oncology and Pathogenesis Program, Memorial Sloan Kettering Cancer Center, New York, NY USA; 776 University Hospital Giessen, Pediatric Hematology and Oncology, Giessen, Germany; 777 Oncologie Sénologie, ICM Institut Régional du Cancer, Montpellier, France; 778 Institute of Clinical Molecular Biology, Christian-Albrechts-University, Kiel, Germany; 779 Institute of Pathology, University of Wuerzburg, Wuerzburg, Germany; 780 Department of Urology, North Bristol NHS Trust, Bristol, UK; 781 SingHealth, Duke-NUS Institute of Precision Medicine, National Heart Centre Singapore, Singapore, Singapore; 782 Department of Computer Science, University of Toronto, Toronto, ON Canada; 783 Bern Center for Precision Medicine, University Hospital of Bern, University of Bern, Bern, Switzerland; 784 Englander Institute for Precision Medicine, Weill Cornell Medicine and New York Presbyterian Hospital, New York, NY USA; 785 Meyer Cancer Center, Weill Cornell Medicine, New York, NY USA; 786 Pathology and Laboratory, Weill Cornell Medical College, New York, NY USA; 787 Vall d’Hebron Institute of Oncology: VHIO, Barcelona, Spain; 788 General and Hepatobiliary-Biliary Surgery, Pancreas Institute, University and Hospital Trust of Verona, Verona, Italy; 789 National Centre for Biological Sciences, Tata Institute of Fundamental Research, Bangalore, India; 790 Indiana University, Bloomington, IN USA; 791 Department of Pathology, GZA-ZNA Hospitals, Antwerp, Belgium; 792 Analytical Biological Services, Inc, Wilmington, DE USA; 793 Sydney Medical School, University of Sydney, Sydney, NSW Australia; 794 cBio Center, Dana-Farber Cancer Institute, Harvard Medical School, Boston, MA USA; 795 Department of Cell Biology, Harvard Medical School, Boston, MA USA; 796 Advanced Centre for Treatment Research and Education in Cancer, Tata Memorial Centre, Navi Mumbai, Maharashtra India; 797 School of Environmental and Life Sciences, Faculty of Science, The University of Newcastle, Ourimbah, NSW Australia; 798 Department of Dermatology, University Hospital of Essen, Essen, Germany; 799 Bioinformatics and Omics Data Analytics, German Cancer Research Center (DKFZ), Heidelberg, Germany; 800 Department of Urology, Charité Universitätsmedizin Berlin, Berlin, Germany; 801 Martini-Clinic, Prostate Cancer Center, University Medical Center Hamburg-Eppendorf, Hamburg, Germany; 802 Department of General Internal Medicine, University of Kiel, Kiel, Germany; 803 German Cancer Consortium (DKTK), Partner site Berlin, Berlin, Germany; 804 Cancer Research Institute, Beth Israel Deaconess Medical Center, Boston, MA USA; 805 University of Pittsburgh, Pittsburgh, PA USA; 806 Department of Ophthalmology and Ocular Genomics Institute, Massachusetts Eye and Ear, Harvard Medical School, Boston, MA USA; 807 Center for Psychiatric Genetics, NorthShore University HealthSystem, Evanston, IL USA; 808 Van Andel Research Institute, Grand Rapids, MI USA; 809 Laboratory of Molecular Medicine, Human Genome Center, Institute of Medical Science, University of Tokyo, Tokyo, Japan; 810 Japan Agency for Medical Research and Development, Tokyo, Japan; 811 Korea University, Seoul, South Korea; 812 Murtha Cancer Center, Walter Reed National Military Medical Center, Bethesda, MD USA; 813 Human Genetics, University of Kiel, Kiel, Germany; 814 Department of Oncologic Pathology, Dana-Farber Cancer Institute, Harvard Medical School, Boston, MA USA; 815 Oregon Health and Science University, Portland, OR USA; 816 Center for RNA Interference and Noncoding RNA, The University of Texas MD Anderson Cancer Center, Houston, TX USA; 817 Department of Experimental Therapeutics, The University of Texas MD Anderson Cancer Center, Houston, TX USA; 818 Department of Gynecologic Oncology and Reproductive Medicine, The University of Texas MD Anderson Cancer Center, Houston, TX USA; 819 University Hospitals Coventry and Warwickshire NHS Trust, Coventry, UK; 820 Department of Radiation Oncology, Radboud University Nijmegen Medical Centre, Nijmegen, GA The Netherlands; 821 Institute for Genomics and Systems Biology, University of Chicago, Chicago, IL USA; 822 Clinic for Hematology and Oncology, St.-Antonius-Hospital, Eschweiler, Germany; 823 Computational and Systems Biology Program, Memorial Sloan Kettering Cancer Center, New York, NY USA; 824 University of Iceland, Reykjavik, Iceland; 825 Division of Computational Genomics and Systems Genetics, German Cancer Research Center (DKFZ), Heidelberg, Germany; 826 Dundee Cancer Centre, Ninewells Hospital, Dundee, UK; 827 Department for Internal Medicine III, University of Ulm and University Hospital of Ulm, Ulm, Germany; 828 Institut Curie, INSERM Unit 830, Paris, France; 829 Department of Gastroenterology and Hepatology, Yokohama City University Graduate School of Medicine, Kanagawa, Japan; 830 Department of Laboratory Medicine, Radboud University Nijmegen Medical Centre, Nijmegen, GA The Netherlands; 831 Division of Cancer Genome Research, German Cancer Research Center (DKFZ), Heidelberg, Germany; 832 Department of General Surgery, Singapore General Hospital, Singapore, Singapore; 833 Cancer Science Institute of Singapore, National University of Singapore, Singapore, Singapore; 834 Department of Medical and Clinical Genetics, Genome-Scale Biology Research Program, University of Helsinki, Helsinki, Finland; 835 East Anglian Medical Genetics Service, Cambridge University Hospitals NHS Foundation Trust, Cambridge, UK; 836 Irving Institute for Cancer Dynamics, Columbia University, New York, NY USA; 837 Institute of Molecular and Cell Biology, Singapore, Singapore; 838 Laboratory of Cancer Epigenome, Division of Medical Science, National Cancer Centre Singapore, Singapore, Singapore; 839 Universite Lyon, INCa-Synergie, Centre Léon Bérard, Lyon, France; 840 Department of Urology, Mayo Clinic, Rochester, MN USA; 841 Royal National Orthopaedic Hospital - Stanmore, Stanmore, Middlesex UK; 842 Department of Biochemistry, Genetics and Immunology, University of Vigo, Vigo, Spain; 843 Giovanni Paolo II / I.R.C.C.S. Cancer Institute, Bari, BA Italy; 844 Neuroblastoma Genomics, German Cancer Research Center (DKFZ), Heidelberg, Germany; 845 Fondazione Policlinico Universitario Gemelli IRCCS, Rome, Italy, Rome, Italy; 846 University of Verona, Verona, Italy; 847 Centre National de Génotypage, CEA - Institute de Génomique, Evry, France; 848 CAPHRI Research School, Maastricht University, Maastricht, ER The Netherlands; 849 Department of Biopathology, Centre Léon Bérard, Lyon, France; 850 Université Claude Bernard Lyon 1, Villeurbanne, France; 851 Core Research for Evolutional Science and Technology (CREST), JST, Tokyo, Japan; 852 Department of Biological Sciences, Laboratory for Medical Science Mathematics, Graduate School of Science, University of Tokyo, Yokohama, Japan; 853 Department of Medical Science Mathematics, Medical Research Institute, Tokyo Medical and Dental University (TMDU), Tokyo, Japan; 854 Cancer Ageing and Somatic Mutation Programme, Wellcome Sanger Institute, Hinxton, UK; 855 University Hospitals Birmingham NHS Foundation Trust, Birmingham, UK; 856 Centre for Cancer Research and Cell Biology, Queen’s University, Belfast, UK; 857 Breast Medical Oncology, The University of Texas MD Anderson Cancer Center, Houston, TX USA; 858 Department of Surgery, Johns Hopkins University School of Medicine, Baltimore, MD USA; 859 Department of Oncology-Pathology, Science for Life Laboratory, Karolinska Institute, Stockholm, Sweden; 860 School of Cancer Sciences, Faculty of Medicine, University of Southampton, Southampton, UK; 861 Department of Gene Technology, Tallinn University of Technology, Tallinn, Estonia; 862 Genetics and Genome Biology Program, SickKids Research Institute, The Hospital for Sick Children, Toronto, ON Canada; 863 Departments of Neurosurgery and Hematology and Medical Oncology, Winship Cancer Institute and School of Medicine, Emory University, Atlanta, GA USA; 864 Department of Clinical and Molecular Medicine, Faculty of Medicine and Health Sciences, Norwegian University of Science and Technology, Trondheim, Norway; 865 Argmix Consulting, North Vancouver, BC Canada; 866 Department of Information Technology, Ghent University, Interuniversitair Micro-Electronica Centrum (IMEC), Ghent, Belgium; 867 Nuffield Department of Surgical Sciences, John Radcliffe Hospital, University of Oxford, Oxford, UK; 868 Institute of Mathematics and Computer Science, University of Latvia, Riga, LV Latvia; 869 Discipline of Pathology, Sydney Medical School, University of Sydney, Sydney, NSW Australia; 870 Department of Applied Mathematics and Theoretical Physics, Centre for Mathematical Sciences, University of Cambridge, Cambridge, UK; 871 Department of Epidemiology and Biostatistics, Memorial Sloan Kettering Cancer Center, New York, NY USA; 872 Department of Statistics, Columbia University, New York, NY USA; 873 Department of Immunology, Genetics and Pathology, Science for Life Laboratory, Uppsala University, Uppsala, Sweden; 874 School of Electronic and Information Engineering, Xi’an Jiaotong University, Xi’an, China; 875 Department of Histopathology, Cambridge University Hospitals NHS Foundation Trust, Cambridge, UK; 876 Oxford NIHR Biomedical Research Centre, University of Oxford, Oxford, UK; 877 Georgia Regents University Cancer Center, Augusta, GA USA; 878 Wythenshawe Hospital, Manchester, UK; 879 Department of Genetics, Washington University School of Medicine, St.Louis, MO USA; 880 Department of Biological Oceanography, Leibniz Institute of Baltic Sea Research, Rostock, Germany; 881 Wellcome Centre for Human Genetics, University of Oxford, Oxford, UK; 882 Department of Molecular and Human Genetics, Baylor College of Medicine, Houston, TX USA; 883 Thoracic Oncology Laboratory, Mayo Clinic, Rochester, MN USA; 884 Institute for Genomic Medicine, Nationwide Children’s Hospital, Columbus, OH USA; 885 Department of Obstetrics and Gynecology, Division of Gynecologic Oncology, Mayo Clinic, Rochester, MN USA; 886 International Institute for Molecular Oncology, Poznań, Poland; 887 Poznan University of Medical Sciences, Poznań, Poland; 888 Genomics and Proteomics Core Facility High Throughput Sequencing Unit, German Cancer Research Center (DKFZ), Heidelberg, Germany; 889 NCCS-VARI Translational Research Laboratory, National Cancer Centre Singapore, Singapore, Singapore; 890 Edison Family Center for Genome Sciences and Systems Biology, Washington University, St. Louis, MO USA; 891 MRC-University of Glasgow Centre for Virus Research, Glasgow, UK; 892 Department of Medical Informatics and Clinical Epidemiology, Division of Bioinformatics and Computational Biology, OHSU Knight Cancer Institute, Oregon Health and Science University, Portland, OR USA; 893 School of Electronic Information and Communications, Huazhong University of Science and Technology, Wuhan, China; 894 Department of Applied Mathematics and Statistics, Johns Hopkins University, Baltimore, MD USA; 895 Department of Cancer Genome Informatics, Graduate School of Medicine, Osaka University, Osaka, Japan; 896 Institute of Computer Science, Heidelberg University, Heidelberg, Germany; 897 School of Mathematics and Statistics, University of Sydney, Sydney, NSW Australia; 898 Ben May Department for Cancer Research, University of Chicago, Chicago, IL USA; 899 Department of Human Genetics, University of Chicago, Chicago, IL USA; 900 Tri-Institutional PhD Program in Computational Biology and Medicine, Weill Cornell Medicine, New York, NY USA; 901 The First Affiliated Hospital, Xi’an Jiaotong University, Xi’an, China; 902 Department of Medicine and Therapeutics, The Chinese University of Hong Kong, Shatin, NT, Hong Kong China; 903 Department of Biostatistics, The University of Texas MD Anderson Cancer Center, Houston, TX USA; 904 Duke-NUS Medical School, Singapore, Singapore; 905 Department of Surgery, Ruijin Hospital, Shanghai Jiaotong University School of Medicine, Shanghai, China; 906 School of Computing Science, University of Glasgow, Glasgow, UK; 907 Division of Orthopaedic Surgery, Oslo University Hospital, Oslo, Norway; 908 Eastern Clinical School, Monash University, Melbourne, VIC Australia; 909 Epworth HealthCare, Richmond, VIC Australia; 910 Department of Biostatistics and Computational Biology, Dana-Farber Cancer Institute and Harvard Medical School, Boston, MA USA; 911 Department of Biomedical Informatics, College of Medicine, The Ohio State University, Columbus, OH USA; 912 The Ohio State University Comprehensive Cancer Center (OSUCCC – James), Columbus, OH USA; 913 The University of Texas School of Biomedical Informatics (SBMI) at Houston, Houston, TX USA; 914 Department of Biostatistics, University of North Carolina at Chapel Hill, Chapel Hill, NC USA; 915 Department of Biochemistry and Molecular Genetics, Feinberg School of Medicine, Northwestern University, Chicago, IL USA; 916 Faculty of Medicine and Health, University of Sydney, Sydney, NSW Australia; 917 Department of Pathology, Erasmus Medical Center Rotterdam, Rotterdam, GD The Netherlands; 918 Division of Molecular Carcinogenesis, The Netherlands Cancer Institute, Amsterdam, CX The Netherlands; 919 Institute of Molecular Life Sciences and Swiss Institute of Bioinformatics, University of Zurich, Zurich, Switzerland

**Keywords:** Cancer genomics, Comparative genomics

## Abstract

Long non-coding RNAs (lncRNAs) are a growing focus of cancer genomics studies, creating the need for a resource of lncRNAs with validated cancer roles. Furthermore, it remains debated whether mutated lncRNAs can drive tumorigenesis, and whether such functions could be conserved during evolution. Here, as part of the ICGC/TCGA Pan-Cancer Analysis of Whole Genomes (PCAWG) Consortium, we introduce the Cancer LncRNA Census (CLC), a compilation of 122 GENCODE lncRNAs with causal roles in cancer phenotypes. In contrast to existing databases, CLC requires strong functional or genetic evidence. CLC genes are enriched amongst driver genes predicted from somatic mutations, and display characteristic genomic features. Strikingly, CLC genes are enriched for driver mutations from unbiased, genome-wide transposon-mutagenesis screens in mice. We identified 10 tumour-causing mutations in orthologues of 8 lncRNAs, including *LINC-PINT* and *NEAT1*, but not *MALAT1*. Thus CLC represents a dataset of high-confidence cancer lncRNAs. Mutagenesis maps are a novel means for identifying deeply-conserved roles of lncRNAs in tumorigenesis.

## Abstract (web summary)

Joana Carlevaro-Fita, Andrés Lanzós et al. present the Cancer LncRNA Census (CLC), a manually curated dataset of 122 long noncoding RNAs (lncRNAs) with experimentally-validated functions in cancer based on data from the ICGC/TCGA Pan-Cancer Analysis of Whole Genomes (PCAWG) Consortium. CLC lncRNAs have unique gene features, and a number display evidence for cancer-driving functions that are conserved from humans to mice.

## Introduction

Tumorigenesis is driven by a series of genetic mutations that promote cancer phenotypes and consequently experience positive selection^1^. The systematic discovery of such driver mutations, and the genes whose functions they alter, has been made possible by tumour genome sequencing. By collecting the entirety of such genes for every cancer type, it should be possible to develop a comprehensive view of underlying processes and pathways, and thereby formulate effective, targeted therapeutic strategies.

The cast of genetic elements implicated in tumorigenesis has recently grown as diverse new classes of non-coding RNAs and regulatory features have been discovered. These include the long non-coding RNAs (lncRNAs), of which tens of thousands have been catalogued^2–5^. LncRNAs are >200 nt long transcripts with no protein-coding capacity. Their evolutionary conservation and regulated expression, combined with a number of well-characterised examples, have together led to the view that lncRNAs are bona fide functional genes^6–9^. Current thinking holds that lncRNAs function by forming complexes with proteins and RNA both inside and outside the nucleus^10,11^.

LncRNAs have been shown to play important roles in various cancers. For example, *MALAT1*, an oncogene across numerous cancers, is restricted to the nucleus and plays a housekeeping role in splicing^12,13^. *MALAT1* is overexpressed in a variety of cancer types, and its knockdown potently reduces not only proliferation but also metastasis in vivo in mouse xenograft assays^14^. *MALAT1* is subjected to elevated mutational rates in human tumours, although it has not yet been established whether these mutations drive tumorigenesis^15,16^. On the other hand, lncRNAs may also function as tumour suppressors. *LincRNA-p21* acts as a downstream effector of p53 regulation through recruitment of the repressor hnRNP-K^17^.

Demonstrably conserved functions between human and mouse is potent evidence for gene’s importance, both in cancer and more generally. For well-known protein-coding genes with cancer roles in human, such as *TP53* and *MYC*, mutations in mouse models can recapitulate the human disease^18,19^. For lncRNAs, evolutionary evidence has been mainly limited to discovery of sequence or positional orthologues, with no evidence for conserved functions^20^. Further doubt has been introduced by the fact that mouse knockouts of iconic cancer-related lncRNAs *MALAT1* and *NEAT1* display little to no aberrant phenotype^21–24^. However, a recent study of human and mouse orthologues of *LINC-PINT* showed that both have tumour-suppressor activity in cell lines, acting through a relatively short, conserved region^25^. Nevertheless, it remains unclear whether this generalises to other identified lncRNAs, and whether mutations in them can induce tumours.

These and other examples of lncRNAs linked to cancer, raise the question of how many more remain to be found amongst the ~99% of annotated lncRNAs that are presently uncharacterised^5,26,27^. Recent tumour genome sequencing studies, in step with advanced bioinformatic driver-gene prediction methods, have yielded hundreds of new candidate protein-coding driver genes^28^. For economic reasons, these studies initially restricted their attention to exomes or the ~2% of the genome covering protein-coding exons^29^. Unfortunately such a strategy ignores mutations in the remaining ~98% of genomic sequence, home to the majority of lncRNAs^5,12^. Driver-gene identification methods rely on statistical models that make a series of assumptions about and simplifications of complex tumour mutation patterns^30^. It is critical to test the performance of such methods using true-positive lists of known cancer driver genes. For protein-coding genes, this role has been fulfilled by the Cancer Gene Census (CGC)^31^, which is collected and regularly updated by manual annotators. Comparison of driver predictions to CGC genes facilitates further method refinement and comparison between methods^32–35^.

In addition to its benchmarking role, the CGC resource has also been useful in identifying unique biological features of cancer genes. For example, CGC genes tend to be more conserved and longer. Furthermore, they are enriched for genes with transcription regulator activity and nucleic acid binding functions^36,37^.

Until very recently, efforts to discover cancer lncRNAs have depended on classical functional genomics approaches of differential expression using microarrays or RNA sequencing^17,27^. While valuable, differential expression per se is not direct evidence for causative roles in tumour evolution. To more directly identify lncRNAs that drive cancer progression, a number of methods, including several within the Pan-Cancer Analysis of Whole Genomes (PCAWG) Network^16^, have recently been developed to search for signals of positive selection using mutation maps of tumour genomes. OncodriveFML utilises nucleotide-level functional impact scores like those inferred from predicted changes in RNA secondary structure together with an empirical significance estimate, to identify lncRNAs with an excess of high-impact mutations^34^. Another method, ExInAtor, identifies candidates with elevated mutational load, using trinucleotide-adjusted local background^15^. Furthermore, The ICGC/TCGA Pan-Cancer Analysis of Whole Genomes (PCAWG) Consortium aggregated whole genome sequencing data from 2658 cancers across 38 tumour types generated by the ICGC and TCGA projects^38^, and applied diverse tools to identify cancer driver lncRNAs^16^. A clear impediment in such analyses has been the lack of true-positive set of known lncRNA driver genes, analogous to CGC. Valuable resources of cancer lncRNAs have been created, notably LncRNADisease^39^ and Lnc2Cancer^40^. These include minimally filtered data from numerous sources, which is beneficial in creating inclusive gene lists, but has drawbacks arising from permissive criteria for inclusion (including expression changes), and inconsistent gene identifiers.

To facilitate the future discovery of cancer lncRNAs, and gain insights into their biology, we have compiled a highly-curated set of cases with roles in cancer processes. Here we present the Cancer LncRNA Census (CLC), the first compendium of lncRNAs with direct functional or genetic evidence for cancer roles. We demonstrate the utility of CLC in assessing the performance of driver lncRNA predictions. Through analysis of this gene set, we demonstrate that cancer lncRNAs have a unique series of features that may in future be used to assist de novo predictions. Finally, we show that CLC genes have conserved cancer roles across the ~80 million years of evolution separating humans and rodents.

## Results

### Definition of cancer-related lncRNAs

As part of recent efforts to identify driver lncRNAs by the Drivers and Functional Interpretation Group (PCAWG-2-5-9-14) within the ICGC/TCGA Pan-Cancer Analysis of Whole Genomes Network (henceforth PCAWG)^16,38^, we discovered the need for a high-confidence reference set of cancer-related lncRNA genes, which we henceforth refer to as cancer lncRNAs. We here present Version 1 of the Cancer LncRNA Census (CLC).

Cancer lncRNAs were identified from the literature using defined and consistent criteria, being direct experimental (in vitro or in vivo) or genetic (somatic or germline) evidence for roles in cancer progression or phenotypes (see Methods). Alterations in expression alone were not considered sufficient evidence. Importantly, only lncRNAs with GENCODE identifiers were included to allow direct integration and comparison between large-scale genomic projects^41^. For every cancer lncRNA, one or more associated cancer types were collected.

Attesting to the value of this approach, we identified several cases in semi-automatically annotated cancer lncRNA databases of lncRNAs that were misassigned GENCODE identifiers, usually with an overlapping protein-coding gene^39^. We also excluded a number of published lncRNAs for which we could not find evidence to meet our criteria, for example *CONCR*, *SRA1* and *KCNQ1OT1*^42–44^. We plan to collect these excluded lncRNAs in future versions of CLC.

**Fig. 1:**
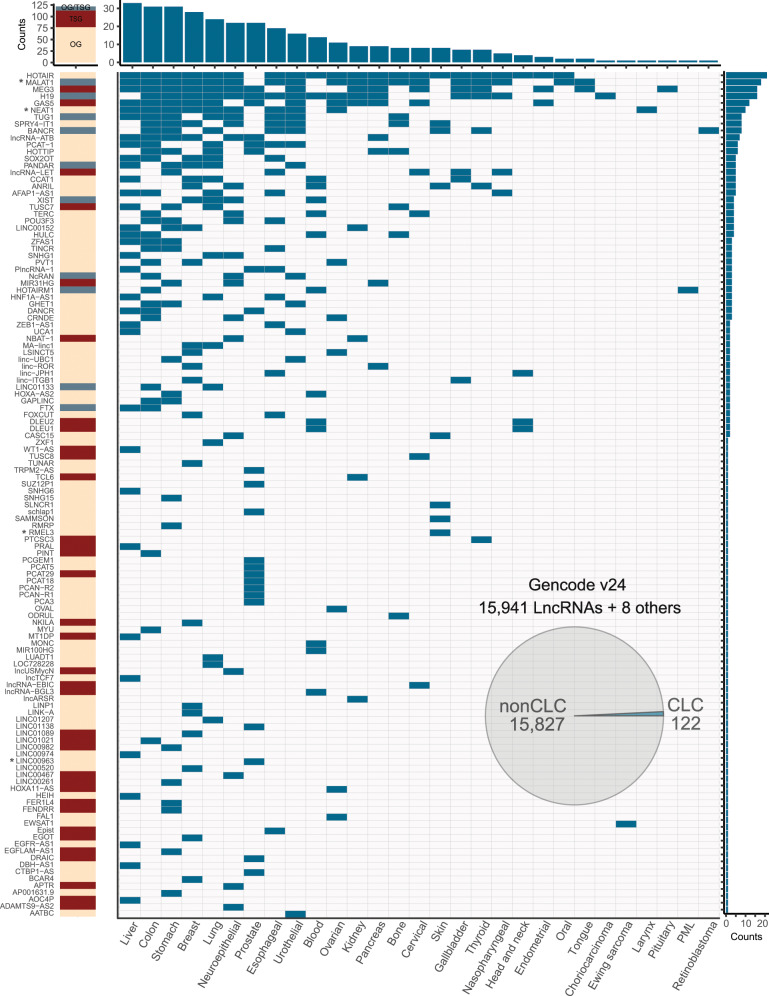
Overview of the Cancer LncRNA Census. Rows represent the 122 CLC genes, columns represent 29 cancer types. Asterisks next to gene names indicate that they are predicted as drivers by PCAWG, based either on gene or promoter evidence (see Supplementary Data 1). Blue cells indicate evidence for the involvement of a given lncRNA in that cancer type. Left column indicates functional classification: tumour suppressor (TSG), oncogene (OG) or both (OG/TSG). Above and to the right, barplots indicate the total counts of each column/row. The piechart shows the fraction that CLC represents within GENCODE v24 lncRNAs. Note that 8 CLC genes are classified as “pseudogenes” by GENCODE. “nonCLC” refers to all other GENCODE-annotated lncRNAs, which are used as background in comparative analyses.

Version 1 of CLC contains 122 lncRNA genes, however, eight of them are annotated as pseudogenes rather than lncRNAs by GENCODE. The remaining 114 CLC genes correspond to 0.72% of a total of 15,941 lncRNA gene loci annotated in GENCODE v24^5,45^ (Fig. 1). For comparison, the Cancer Gene Census (CGC) (COSMIC v78, downloaded 3 October 2016) lists 561 or 2.8% of protein-coding genes^31^. The entire remaining set of 15,827 lncRNA loci is henceforth referred to as non-CLC (Fig. 1). The full CLC dataset is found in Supplementary Data 1.

The cancer classification terminology used amongst the source literature for CLC was not uniform. Therefore, using the International Classification of Diseases for Oncology^46^, we reassigned the cancer types described in the original research articles to a reduced set of 29 (Fig. 1 and Supplementary Fig. 1).

Altogether, CLC contains 333 unique lncRNA-cancer type relationships. Out of 122 genes, 77 (63.1%) were shown to function as oncogenes, 35 (28.7%) as tumour suppressors, and 10 (8.2%) with evidence for both activities depending on the tumour type (Fig. 1 and Supplementary Fig. 1). It is unclear whether the difference in the frequencies of oncogenes and tumour suppressors has a biological explanation, or is simply the result of ascertainment bias. For protein-coding genes in the CGC (COSMIC v85, downloaded 25 May 2018), approximately equal numbers of oncogenes and tumour-suppressor genes are recorded (43% and 44%, respectively). It is important to take into account that the oncogene and tumour-suppressor classifications were deduced from the collected references. While a gene has shown oncogenic properties in a particular cancer type, future publications could show that it functions as tumour suppressor in a different tissue, for example, the most studied lncRNAs in CLC (top of Fig. 1) are enriched in dual functions.

The most prolific lncRNAs, with ≥16 recorded cancer types, are *HOTAIR*, *MALAT1*, *MEG3* and *H19* (Fig. 1 and Supplementary Fig. 1). It is not clear whether this reflects their unique pan-cancer functionality, or is simply a result of their being amongst the most early-discovered and widely-studied lncRNAs.

In vitro experiments were the most frequent evidence source, usually consisting of RNAi-mediated knockdown in cultured cell lines, coupled to phenotypic assays such as proliferation or migration (Supplementary Fig. 1). Far fewer have been studied in vivo, or have cancer-associated somatic or germline mutations. Nineteen lncRNAs had three or more independent evidence sources (Supplementary Fig. 1).

### CLC and other databases

**Fig. 2:**
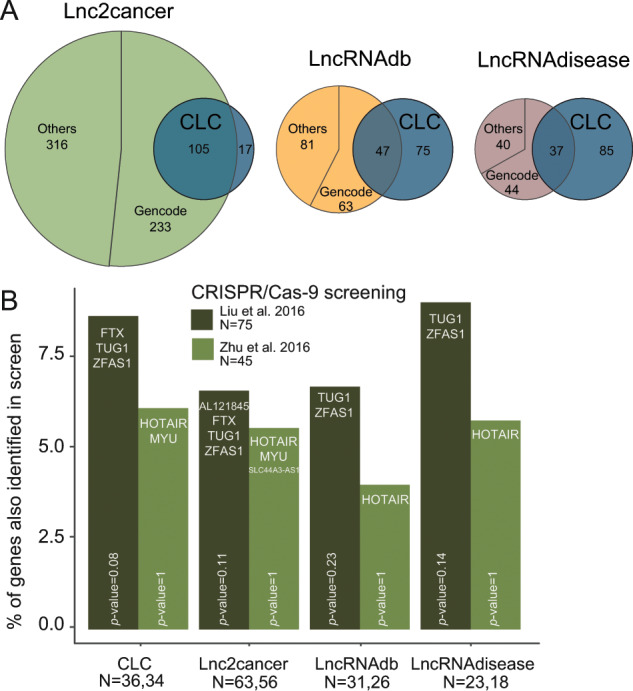
Intersection of CLC with public databases. **a** Proportional Venn diagrams displaying the overlap between CLC set and the three indicated databases. Shown are the total numbers of unique human lncRNAs contained in each intersection (note that for LncRNADisease, numbers refer only to cancer-related genes). Databases are divided into genes that belong to GENCODE v24 annotation and others. **b** Barplot shows the percent of GENCODE v24 lncRNAs of each database that is present in the final list of cancer lncRNA candidates of two CRISPR/Cas-9 cancer screenings (Liu et al.^9^ and Zhu et al.^47^). *N* represents the number of GENCODE v24 lncRNAs from each database that were tested in each of the two CRISPR/Cas-9 screenings. Names of the genes that overlap between the databases and the screenings are shown in each bar. *p*-values were calculated using Fisher’s exact test.

There are a number of relevant lncRNA databases presently available: the Lnc2Cancer database (*n* = 654)^40^, the LncRNADisease database (*n* = 121)^39^ and lncRNAdb (*n* = 191)^26^. CLC covers between 17% and 31% of these databases (Lnc2Cancer and LncRNADisease, respectively) but none of these resources contain the complete list of genes presented here (Fig. 2a). It is important to note that the other databases also include a minority of non-GENCODE genes, ranging from 40 to 316 (33 and 48%) (Fig. 2a). In addition, we intersected the four databases (Supplementary Fig. 2) using only GENCODE-annotated genes. It is clear that CLC has the greatest overlap with the other three, suggesting that it has the greatest specificity.

We sought to use recent unbiased proliferation screen data to independently compare cancer lncRNA databases^9,47^. Using only GENCODE-annotated genes, CLC is the resource that overall has the most nearly-significant (*p*-value = 0.08, Fisher’s exact test) fraction of independently-identified proliferation lncRNAs, although the sparse nature of the data means that this conclusion is not definitive (Fig. 2b).

Finally, we downloaded and collected 8416 bioinformatically-predicted Gencode v24 lncRNAs from a recent TCGA publication^48^, but found no significant overlap with CLC (69 gene; *p-*value = 0.13, Fisher’s exact test).

### CLC for benchmarking lncRNA driver prediction methods

One of the primary motivations for CLC is to develop a high-confidence functional set for benchmarking and comparing methods for identifying driver lncRNAs. In the domain of protein-coding driver-gene predictions, the Cancer Gene Census (CGC) has become such a gold standard training set^31^. Typically, the predicted driver genes belonging to CGC are judged to be true positives, and the fraction of these amongst predictions is used to estimate the positive predictive value (PPV), or precision. This measure can be calculated for increasing cutoff levels, to assess the optimal cutoff.

**Fig. 3:**
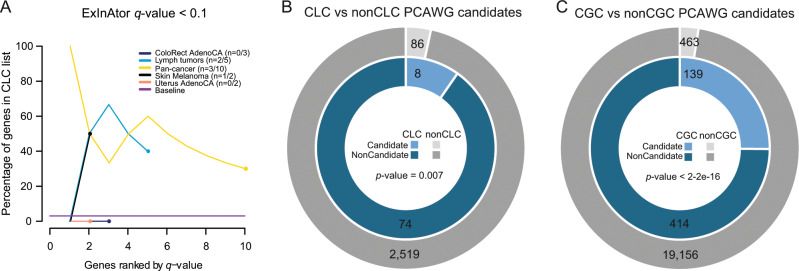
CLC as benchmark for cancer driver predictions. **a** CLC benchmarking of ExInAtor driver lncRNA predictions using PCAWG whole genome tumours at *q*-value (false discovery rate) cutoff of 0.1. Genes sorted increasingly by *q*-value are ranked on *x*-axis. Percentage of CLC genes amongst cumulative set of predicted candidates at each step of the ranking (precision), are shown on the *y*-axis. Black line shows the baseline, being the percentage of CLC genes in the whole list of genes tested. Coloured dots represent the number of candidates predicted under the *q*-value cutoff of 0.1. “*n*” in the legend shows the number of CLC and total candidates for each cancer type. **b** Rate of driver-gene predictions amongst CLC and non-CLC genesets (*q*-value cutoff of 0.1) by all the individual methods and the combined list of drivers developed in PCAWG. *p*-value is calculated using Fisher’s exact test for the difference between CLC and non-CLC genesets. **c** Rate of driver-gene predictions amongst CGC and nonCGC genesets (*q*-value cutoff of 0.1) by all the individual methods and the combined list of drivers developed in PCAWG. *p*-value is calculated using Fisher’s exact test for the difference between CGC and nonCGC genesets.

First, we used CLC to examine the performance of the lncRNA driver predictor ExInAtor^15^ in recalling CLC genes using PCAWG tumour mutation data^16^. A total of 2687 GENCODE lncRNAs were tested here, of which 82 (3.1%) belong to CLC. Driver predictions on several cancers at the standard false discovery rate (*q*-value) cutoff of 0.1 are shown for selected cancers in Fig. 3a. That panel shows the CLC-defined precision (*y*-axis) as a function of predicted driver genes ranked by *q*-value (*x*-axis). We observe rather heterogeneous performance across cancer cohorts. This may reflect a combination of intrinsic biological differences and differences in cohort sizes, which differs widely between the datasets shown. For the merged pan-cancer dataset, ExInAtor predicted three CLC genes amongst its top ten candidates (*q*-value *<* 0.1), a rate far in excess of the background expectation (baseline, fraction of all lncRNAs in CLC). Similar enrichments are observed for other cancer types. These results support both the predictive value of ExInAtor, and the usefulness of CLC in assessing lncRNA driver predictors. In addition, we repeated the same analysis for each of the three mentioned databases (lnc2cancer, lncRNAdb and lncRNAdisease) (*q*-value *<* 0.2) (Supplementary Fig. 3). The precision level of all databases is around 40%, except lncRNAdisease that shows the overall lowest precision. As deduced from Fig. 2, the low number of intersecting genes does not allow a definitive conclusion. However, it is interesting to notice that CLC shows a similar performance to the other databases in terms of sensitivity while increasing specificity. This is likely due to the stringent, function-based inclusion criteria of CLC.

Finally, we assessed the precision (i.e. positive predictive value) of PCAWG lncRNA and protein-coding driver predictions across all cancers and all prediction methods^16^. Using a *q*-value cutoff of 0.1, we found that across all cancer types and methods, a total of 8 (8.5%) of lncRNA predictions belong to CLC (Fig. 3b), while a total of 139 (23.1%) of protein-coding predictions belong to CGC (Fig. 3c). In terms of sensitivity, 9.8% and 25.1% of CLC and CGC genes are predicted as candidates, respectively. Despite the lower detection of CLC genes in comparison with CGC genes, both sensitivity rates significantly exceed the prediction rate of non-CLC and nonCGC genes (*p*-value = 0.007 and *p*-value < 0.001 Fisher’s exact tests, respectively), again highlighting the usefulness of the CLC gene set (Fig. 3c).

### CLC genes are distinguished by function- and disease-related features

**Fig. 4:**
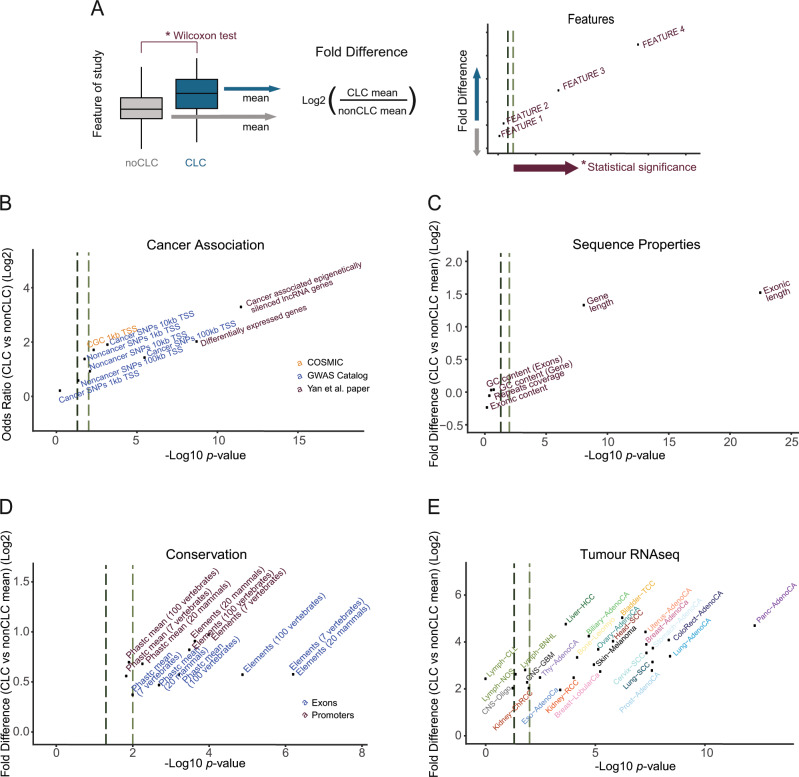
Distinguishing features of CLC genes. **a** Panel showing a hypothetic feature analysis example to illustrate the content of the following figures. All panels in this figure display features (dots), plotted by their log-fold difference (odds ratio in case of panel (**b**)) between CLC/non-CLC genesets (*y*-axis) and statistical significance (*x*-axis). In all plots dark and light green dashed lines indicate 0.05 and 0.01 significance thresholds, respectively. **b** Cancer and non-cancer disease-related data from indicated sources: *y*-axis shows the log2 of the odds ratio obtained by comparing CLC to non-CLC by Fisher’s exact test; *x-*axis displays the estimated *p*-value from the same test. “CGC 1 kb TSS” refers to the fraction of genes that have a nearby known CGC cancer protein-coding gene. This is explored in more detail in the next Figure. “Non-cancer SNPs” refers to GWAS SNPs associated with diseases/traits other than cancer. **c** Sequence and gene properties: *y*-axis shows the log2 fold difference of CLC/non-CLC means; *x*-axis represents the *p*-value obtained. **d** Evolutionary conservation: “Phastc mean” indicates average base-level PhastCons score; “Elements” indicates percent coverage by PhastCons conserved elements (see Methods). Colours distinguish exons (blue) and promoters (purple). **e** Tumour RNA-seq: expression levels of lncRNA genes in different cancer tissues obtained from RNA-seq expression data from PCAWG. For (**b**–**d**), statistical significance was calculated using Wilcoxon test.

We recently found evidence, using a smaller set of cancer-related LncRNAs (CRLs), that cancer lncRNAs are distinguished by various genomic and expression features indicative of biological function^15^. We here extended these findings using a large series of potential gene features, to search for those features distinguishing CLC from non-CLC lncRNAs (Fig. 4a).

First, associations with expected cancer-related features were tested (Fig. 4b). CLC genes are significantly more likely to have their transcription start site (TSS) within 100 kb of cancer-associated germline SNPs (cancer SNPs 100 kb TSS), and more likely to be either differentially expressed or epigenetically-silenced in tumours^49^ (Fig. 4b). Intriguingly, we observed a tendency for CLC lncRNAs to be more likely to lie within 1 kb of known cancer protein-coding genes (CGC 1 kb TSS). While searching for additional evidence of functionality for CLC genes, we found that they are significantly closer to non-cancer, phenotype-associated germline SNPs (non-cancer SNPs 100 kb TSS) in comparison with non-CLC genes (Fig. 4b). Proximity to cancer and non-cancer SNPs support the both cancer roles and general biological functionality of CLC genes.

We next investigated the properties of the genes themselves. As seen in Fig. 4c, and consistent with our previous findings^15^, CLC genes (gene length) and their spliced products (exonic length) are significantly longer than average. No difference was observed in the ratio of exonic to total length (exonic content), nor overall exon repetitive sequence coverage (repeats coverage), nor GC content.

CLC genes also tend to have greater evidence of function, as inferred from evolutionary conservation. Base-level conservation at various evolutionary depths was calculated for lncRNA exons and promoters (Fig. 4d). Across all measures tested, using either average base-level scores or percent coverage by conserved elements, we found that CLC genes’ exons are significantly more conserved than other lncRNAs (Fig. 4d). The same was observed for conservation of promoter regions.

High levels of gene expression in normal tissues are known to correlate with lncRNA conservation, and are hypothesized to be a reflection of functionality^50^. In addition, genes with oncogenic roles tend to be highly expressed in cancer samples^36^. We found that CLC has consistently higher steady-state expression levels compared with non-CLC genes across PCAWG tumours (Fig. 4e), as well as healthy organs and cultured cell lines (Supplementary Fig. 4). As deduced from proximity to cancer and non-cancer SNPs, high levels of expression in cancer and normal samples reflect important functionality for CLC genes.

Finally, we investigated whether CLC transcripts might be initiated by any types of Transposable Elements (TEs) (see Methods). We found that CLC TSSs are enriched for one category, “Simple repeats” (Supplementary Fig. 5).

### Evidence for genomic clustering of non-coding and protein-coding cancer genes

In light of recent evidence for colocalisation and coexpression of disease-related lncRNAs and protein-coding genes^51^, we were curious whether such an effect holds for cancer-related lncRNAs and protein-coding genes. We asked, more specifically, whether CLC genes tend to be closer to CGC genes than expected by chance, and whether this is manifested in a more co-regulated expression.

**Fig. 5:**
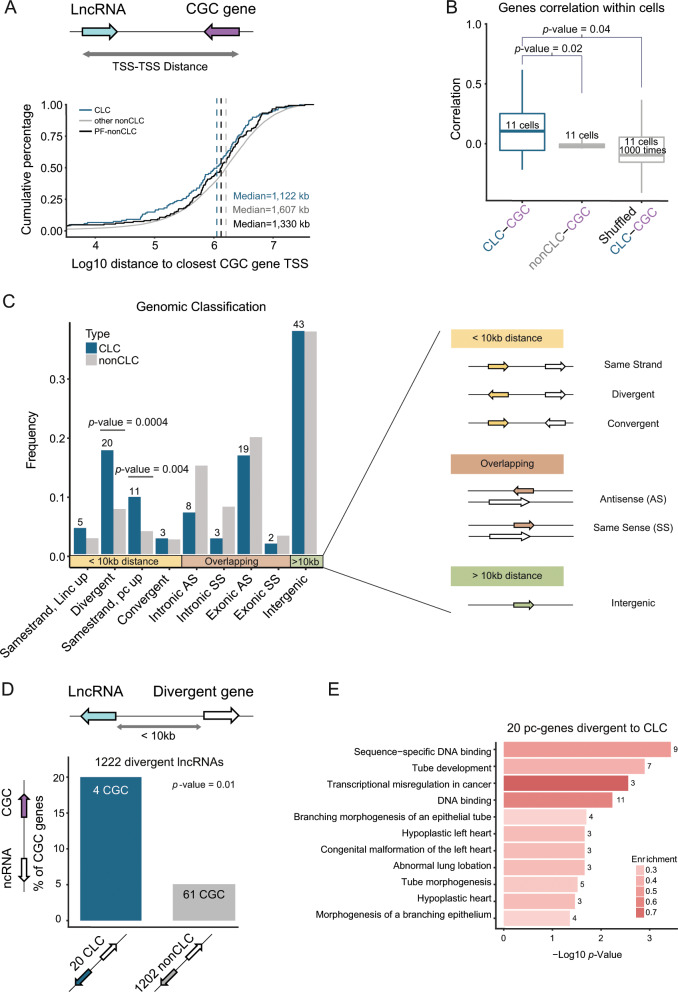
Evidence for genomic clustering of non-coding and protein-coding cancer genes. **a** Cumulative distribution of the genomic distance of lncRNA transcription start site (TSS) to the closest Cancer Gene Census (CGC) (protein-coding) gene TSS. LncRNAs are divided into CLC (*n* = 122), potentially functional non-CLC genes (PF-non-CLC) (*n* = 149), and other non-CLC genes (*n* = 15,678). **b** Boxplot shows the distribution of the gene expression correlation between CLC and their closest CGC genes in 11 human cell lines, including two control analyses (distance-matched non-CLC-CGC pairs, and shuffled CLC-CGC pairs). Correlation was calculated for gene pairs within each cell type, using Pearson method. *p*-value for Kolmogorov–Smirnov test is shown. **c** Genomic classification of lncRNAs. Genes are classified according to distance and orientation to the closest protein-coding gene, and these are grouped into three categories: genes closer than 10 kb to closest protein-coding gene, genes overlapping a protein-coding gene and intergenic genes (>10 kb from closest protein-coding gene). *p*-values for Fisher’s exact tests are shown. **d** The percentage of divergent CLC (left bar) and non-CLC (right bar) genes divergent to a cancer protein-coding gene (CGC). Numbers represent numbers of genes with which the percentage is calculated. *p*-value for Fisher’s exact test is shown. **e** Functional annotations of the 20 protein-coding genes (pc-genes) divergent to CLC genes from panel (**c**). Bars indicate the –log10 (corrected) *p*-value (see Methods) and are coloured based on the “enrichment”: the number of genes that contain the functional term divided by the total number of queried genes. Numbers at the end of the bars correspond to the number of genes that fall into the category.

To this aim, we computed TSS-TSS distances from lncRNAs to protein-coding genes and we found that CLC genes on average tend to lie moderately closer to protein-coding genes of all types, compared with non-CLC lncRNAs (Supplementary Fig. 6A, B). Since CLC genes are enriched for functional features (i.e. expression and conservation), we could not rule out the possibility that proximity to protein-coding genes is a feature of functional lncRNAs rather than cancer lncRNA genes. In order to further investigate this possibility, we repeated the analysis dividing the non-CLC set into potentially functional non-CLC genes (PF-non-CLC) (non-CLC genes sampled to match CLC expression and conservation, *N* = 149, Supplementary Fig. 7) and “other nonCLC” (the rest of non-CLC). Interestingly, when comparing distances to any type of protein-coding genes, both CLC and PF-non-CLC are significantly closer than the rest of lncRNA (Wilcoxon test, *p*-value = 0.03 and 0.007, respectively), being the PF-non-CLC genes the closest ones (median 21.9, 29 and 37.8 kb, for PF-non-CLC, CLC and other non-CLC, respectively) (Supplementary Fig. 6C). However, when assessing specifically for distance to CGC genes, only CLC set is significantly closer than the rest of lncRNAs (Wilcoxon test, *p*-value = 0.0008) and it represents the group with the lowest distance (median 1122, 1330 and 1607 kb for CLC, PF-non-CLC and other non-CLC, respectively) (Fig. 5a). Thus, although proximity to protein-coding genes seems to be a feature of potentially functional lncRNAs, CLC genes are closer to cancer genes compared with other lncRNAs with similar function-like properties.

It has been widely proposed that proximal lncRNA/protein-coding gene pairs are involved in *cis*-regulatory relationships, which is reflected in expression correlation^52^. We next asked whether proximal CLC-CGC pairs exhibit this behaviour. An important potential confounding factor, is the known positive correlation between nearby gene pairs^53^, and this must be controlled for. Using gene expression data across 11 human cell lines, we observed a positive correlation between CLC-CGC gene pairs for each cell type (Fig. 5b). To control for the effect of proximity on correlation, we next randomly sampled a similar number of non-CLC lncRNAs with matched distances (TSS-TSS) from the same CGC genes, and found that this correlation was lost (Fig. 5b, “nonCLC-CGC”). To further control for a possible correlation arising from the simple fact that both CGC and CLC genes are involved in cancer, and CLC genes are in general enriched for conservation and expression, we next randomly shuffled the CLC-CGC pairs 1000 times, again observing no correlation (Fig. 5b, “Shuffled CLC-CGC”). Together these results show that genomically proximal protein-coding/non-coding gene pairs exhibit an expression correlation that exceeds that expected by chance, even when controlling for genomic distance.

These results prompted us to further explore the genomic localization of CLC genes relative to their proximal protein-coding gene and the nature of their neighbouring genes. Next, we observed an unexpected difference in the genomic organisation of CLC genes: when classified by orientation with respect to nearest protein-coding gene^5^, we found a significant enrichment of CLC genes immediately downstream and on the same strand as protein-coding genes (“Samestrand, pc up”, Fig. 5c). Moreover, CLC genes are approximately twice as likely to lie in an upstream, divergent orientation to a protein-coding gene (“Divergent”, Fig. 5c). Of these CLC genes, 20% are divergent to a CGC gene, compared with 5% for non-CLC genes (*p*-value = 0.018, Fisher’s exact test) (Fig. 5d), and several are divergent to protein-coding genes that have also been linked or defined to be involved in cancer, despite not being classified as CGCs (Supplementary Data 2).

Given this noteworthy enrichment of CGC genes among the divergent protein-coding genes of the CLC set, we next inspected the functional annotation of those protein-coding genes. Examining their Gene Ontology (GO) terms, molecular pathways and other gene function related terms, we found this group of genes to be enriched in GO terms for “sequence-specific DNA binding”, “DNA binding”, “tube development” and “transcriptional misregulation in cancer” (Fig. 5e and Supplementary Data 3), contrary to the GO terms of the divergent protein-coding genes of the non-CLC set (Supplementary Data 4). These results were confirmed by another, independent GO-analysis suite (see Methods). Interestingly, three out of the top four functional groups were observed previously in a study of protein-coding genes divergent to long upstream antisense transcripts in primary mouse tissues^54^.

Thus, CLC genes appear to be non-randomly distributed with respect to protein-coding genes, and particularly their CGC subset.

### Evidence for anciently conserved cancer roles of lncRNAs

In mouse, numerous studies have employed unbiased forward genetic screens to identify genes that either inhibit or promote tumorigenesis^55^. These studies use engineered, randomly-integrating transposons carrying bidirectional polyadenylation sites as well as strong promoters. Insertions, or clusters of insertions, called “common insertion sites” (CIS) that are identified in sequenced tumour DNA, are assumed to act as driver mutations^55^, and thereby implicate the overlapping or neighbouring gene locus as either an oncogene or tumour-suppressor gene. Although these studies have traditionally been focused on identifying protein-coding driver genes, they can in principle also identify non-coding RNA driver loci^55^.

**Fig. 6:**
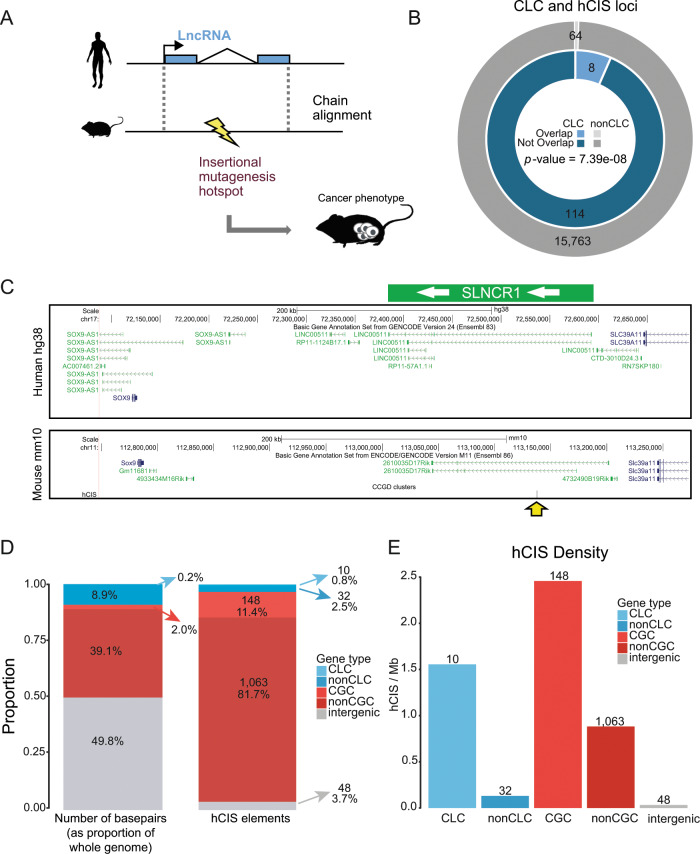
Evidence for ancient conserved cancer roles of lncRNAs. **a** Functional conservation of human CLC genes was inferred by the presence of Common Insertion Sites (CIS), identified in transposon-mutagenesis screens, at orthologous regions in the mouse genome. Orthology was inferred from Chain alignments and identified using LiftOver utility. **b** Number of CLC and non-CLC genes that contain human orthologous common insertion sites (hCIS) (see Table 1). Significance was calculated using Fisher’s exact test. **c** UCSC browser screenshot of a CLC gene (*SLNCR1*, ENSG00000227036) intersecting a CIS (yellow arrow). **d** Number of basepairs and number of overlapping hCIS for cancer driver protein-coding genes (CGC), non-cancer driver protein-coding genes (nonCGC), cancer-related lncRNAs (CLC), rest of GENCODE lncRNAS (non-CLC) and the rest of the genome that do not overlap any of the previous element types (intergenic). Arrows indicate the number of hCIS and the percentage for each element type. **e** Number of overlapping hCIS per megabase of genomic span for each gene class.

We thus reasoned that comparison of mouse CISs to orthologous human regions could yield independent evidence for the functionality of human cancer lncRNAs (Fig. 6a). To test this, we collected a comprehensive set of CISs in mouse^56^, consisting of 2906 loci from seven distinct cancer types (Supplementary Data 5). These sites were then mapped to orthologous regions in the human genome, resulting in 1301 non-overlapping human CISs, or hCISs. 6.9% (90) of these CISs lie outside of protein-coding gene boundaries.

Mapping hCISs to lncRNA annotations, we discovered altogether eight CLC genes (6.6%) carrying at least one insertion within their gene span: *DLEU2*, *GAS5*, *MONC*, *NEAT1*, *PINT*, *PVT1*, *SLNCR1*, *XIST* (Table 1). Two cases, *DLEU2* and *MONC*, each have two independent hCIS sites. In contrast, just 64 (0.4%) non-CLC lncRNAs contained hCISs (Fig. 6b). A good example is *SLNCR1*, shown in Fig. 6c, which drives invasiveness of human melanoma cells^57^, and whose mouse orthologue contains a CIS discovered in pancreatic cancer. It is noteworthy that no hCIS was found to overlap *MALAT1* despite its being amongst the most widely-studied cancer lncRNAs^14^. This agrees with the lack of strong phenotypic effects when deleting this gene in mouse models, as discussed in the Introduction^21–23^. We examined the possibility that hCIS insertions in these CLC genes could in fact be caused by nearby, protein-coding cancer genes. However, none of these eight CLC genes are within 100 kb of a CGC gene, with the exception of *PVT1* lncRNA, lying 58 kb from *c-MYC* oncogene.

**Table 1:** List of intergenic CIS human (GRCh38)/mouse (GRCm38) gene pairs.

Human CLC name	Human CLC ID	Chr human	Start human	End human	Chr mouse	Start mouse	End mouse	PubMed ID	Cancer type mouse
*DLEU2*	ENSG00000231607	chr13	50,048,971	50,049,063	chr14	61,631,880	61,631,972	24316982	Liver
*DLEU2*	ENSG00000231607	chr13	50,049,117	50,049,206	chr14	61,632,026	61,632,110	24316982	Liver
*GAS5*	ENSG00000234741	chr1	173,864,370	173,864,435	chr1	161,038,091	161,038,156	25961939	Sarcoma
*MONC*	ENSG00000215386	chr21	16,539,096	16,539,161	chr16	77,598,935	77,599,000	23685747	Nervous System
*MONC*	ENSG00000215386	chr21	16,561,654	16,561,655	chr16	77,616,439	77,616,440	24316982	Liver
*NEAT1*	ENSG00000245532	chr11	65,444,511	65,444,512	chr19	5,825,497	5,825,498	24316982	Liver
*PINT*	ENSG00000231721	chr7	131,049,455	131,049,456	chr6	31,179,149	31,179,150	22699621	Pancreatic
*PVT1*	ENSG00000249859	chr8	128,007,970	128,007,971	chr15	62,186,646	62,186,647	22699621	Pancreatic
*SLNCR1*	ENSG00000227036	chr17	72,507,275	72,507,276	chr11	113,137,613	113,137,614	22699621	Pancreatic
*XIST*	ENSG00000229807	chrX	73,841,539	73,841,540	chrX	103,473,862	103,473,863	24316982	Liver

This analysis would suggest that CLC genes are enriched for hCISs; however, there remains the possibility that this is confounded by their greater length and possible overlap with protein-coding genes. To account for this, we only selected hCIS elements that do not overlap protein-coding regions (90 hCIS) and we performed two separate validations using only regions that do not overlap protein-coding genes from the CLC and non-CLC genesets. First, groups of non-CLC genes with CLC-matched length were randomly sampled, and the number of intersecting hCISs per unit gene length (Mb) was counted (Supplementary Fig. 8A). Second, CLC genes were randomly relocated in the genome, and the number of genes intersecting at least one hCIS was counted (Supplementary Fig. 8B). Both analyses showed that the number of intersecting hCISs per Mb of CLC gene span is far greater than expected in comparison with both non-CLC genes (Supplementary Fig. 8A) and intergenic space (nucleotides that do not overlap neither lncRNAs neither protein-coding genes) (Supplementary Fig. 8B). Interestingly, non-CLC genes also show an enrichment for hCIS sites in comparison with intergenic regions (Supplementary Fig. 8C), suggesting that more cancer lncRNAs remain to be discovered.

We further compared the enrichment of hCIS in protein-coding genes, lncRNA genes and other intergenic space. Compared with the genomic space they occupy, there is a clear enrichment of hCIS elements in both protein-coding CGC genes, as well as CLC lncRNAs (Fig. 6d). Expressed as insertion rate per megabase of gene span, it is clear that CLC genes are targeted more frequently than background intergenic DNA and non-cancer-related lncRNA genes. Of note are the non-background insertion rates for non-cancer-related protein-coding (nonCGC) and lncRNA genes (non-CLC), suggesting that there remain substantial numbers of undiscovered cancer genes in both groups.

Together these analyses demonstrate that CLC genes are orthologous to mouse cancer-causing genomic loci at a rate greater than expected by random chance. These identified cases, and possibly other CLC genes, display cancer functions that have been conserved over tens of millions of years since human-rodent divergence.

## Discussion

We have presented the Cancer LncRNA Census, the first controlled set of GENCODE-annotated lncRNAs with demonstrated roles in tumorigenesis or cancer phenotypes.

The present state of knowledge of lncRNAs in cancer, and indeed lncRNAs generally, remains incomplete. Consequently, our aim was to create a gene set with the greatest possible confidence, by eliminating the relatively large number of published cancer lncRNAs with as-yet unproven functional roles in disease processes. Thus, we defined cancer lncRNAs as those having direct experimental or genetic evidence supporting a causative role in cancer phenotypes. By this measure, gene expression changes alone do not suffice. By introducing these well-defined inclusion criteria, we hope to ensure that CLC contains the highest possible proportion of bona fide cancer genes, giving it maximum utility for de novo predictor benchmarking. In addition, its basis in GENCODE ensures portability across datasets and projects. Inevitably some well-known lncRNAs did not meet these criteria (including *SRA1*, *CONCR, KCNQ1OT1*)^42–44^; these may be included in future when more validation data becomes available. We believe that CLC will complement the established lncRNA databases such as lncRNAdb, LncRNADisease and Lnc2Cancer, which are more comprehensive, but are likely to have a higher false-positive rate due to their more relaxed inclusion criteria^26,39,40^.

De novo lncRNA driver-gene discovery is likely to become increasingly important as the number of sequenced tumours grow. The creation and refinement of statistical methods for driver-gene discovery will depend on the available of high-quality true-positive genesets such as CLC. It will be important to continue to maintain and improve the CLC in step with anticipated growth in publications on validated cancer lncRNAs. Very recently, CRISPR-based screens^9,47^ have catalogued large numbers of lncRNAs contributing to proliferation in cancer cell lines, which will be incorporated in future versions.

We used CLC to estimate the performance of de novo driver lncRNA predictions from the PCAWG project, made using the ExInAtor pipeline^15^. Supporting the usefulness of this approach, we found an enrichment for CLC genes amongst the top-ranked driver predictions. Extending this to the full set of PCAWG driver predictors, approximately ten percent of CLC genes (9.8%) are called as drivers by at least one method^16^, which is lower to the rate of CGC genes identified (25.1%).

The low rate of concordance between de novo predictions and CLC genes may be due to technical or biological factors. Indeed, it is important to state that we do not yet know whether CLC holds “cancer driver” lncRNAs, and indeed, how many such genes exist. In principle, lncRNAs may play two distinct roles in cancer: first, as driver genes, defined as those whose mutations are early and positively-selected events in tumorigenesis; or second, as “downstream genes”, which do make a genuine contribution to cancer phenotypes, but through non-genetic alterations in cellular networks resulting from changes in expression, localisation or molecular interactions. These downstream genes may not display positively-selected mutational patterns, but would be expected to display cancer-specific alterations in expression. A key question for the future is how lncRNAs break down between these two categories, and the utility of CLC in benchmarking de novo driver predictions will depend on this. However, the identification of lncRNAs whose silencing or overexpression is sufficient for tumour formation in mouse, would seem to suggest that they are true “driver genes”.

Analysis of the CLC gene set has broadened our understanding of the unique features of cancer lncRNAs, and generally supports the notion that lncRNAs have intrinsic biological functionality. Cancer lncRNAs are distinguished by a series of features that are consistent with both roles in cancer (e.g. tumour expression changes), and general biological functionality (e.g. high expression, evolutionary conservation). Elevated evolutionary conservation in the exons of CLC genes would appear to support their functionality as a mature RNA transcript, in contrast to the act of their transcription alone^58^. Another intriguing observation has been the colocalisation of cancer lncRNAs with known protein-coding cancer genes: these are genomically proximal and exhibit elevated expression correlation. This points to a regulatory link between cancer lncRNAs and protein-coding genes, perhaps through chromatin looping, as described in previous reports for *CCAT1* and *MYC*, for example^59^.

One important caveat for all features discussed here is ascertainment bias: almost all lncRNAs discussed have been curated from published, single-gene studies. It is entirely possible that selection of genes for initial studies was highly non-random, and influenced by a number of factors—including high expression, evolutionary conservation and proximity to known cancer genes—that could bias our inference of lncRNA features. This may be the explanation for the observed excess of cancer lncRNAs in divergent configuration to protein-coding genes. However, the general validity of some of the CLC-specific features described here—including high expression and evolutionary conservation—were also observed in recent unbiased genome-wide screens^9,15^, suggesting that they are genuine.

Despite the relatively low concordance of CLC genes with PCAWG driver predictions, the results of this study strongly support the value and key cancer role of identified lncRNAs in cancer. Most notably, the existence of a core set of eight lncRNAs with independently-identified mouse orthologues with similar cancer functions, is a powerful evidence that these genes are bona fide cancer genes, whose overexpression or silencing can drive tumour formation. To our knowledge this is the most direct demonstration to date of anciently conserved functions and disease roles for lncRNAs. It will be intriguing to investigate in future whether more human-mouse orthologous lncRNAs have been identified in such screens.

## Methods

### Manual curation

All lncRNAs in lncRNAdb and those listed in Schmitt and Chang’s recent review article were collected^26,60^. To these were added all cases from LncRNADisease and Lnc2Cancer databases^39,40^. This primary list formed the basis for a manual literature search: all available publications for each gene were identified by keyword search in PubMed. If publications were found conforming to at least one of the inclusion criteria (below) and the gene has a GENCODE ID, then it was added to CLC, with appropriate information on the associated cancer, biological activity. For the numerous cases where no GENCODE ID was supplied in the original publication, any available ID, or primer or siRNA sequence was used to identify the gene using the UCSC Genome Browser Blat tool^61^.

Inclusion criteria sufficient to define a cancer lncRNA and link it to a cancer type were

Class t: In vitro demonstration that their knockdown and/or overexpression in cultured cancer cells results in changes to cancer-associated phenotypes. These typically include proliferation rates, migration, sensitivity to apoptosis, or anchorage-independent growth.

Class v: In vivo demonstration that their knockdown and/or overexpression in cancer cells alters their tumorigenicity when injected into animal models.

Class g: Germline mutations or variants that predispose humans to cancer.

Class s: Somatic mutations that show evidence for positive selection during tumour formation.

An additional criterion was allowed to link an lncRNA to a cancer type, only if at least one of the above criteria was already met for another cancer:

Class p: Prognosis, the lncRNAs expression is statistically linked to disease progression or response to treatment.

If an lncRNA was found to promote tumorigenesis or cancer phenotype, it was defined as “oncogene”. Conversely those found to inhibit such phenotypes were defined as “tumour suppressor”. Several lncRNAs were found to have both activities recorded in different cancer types, and were given both labels. For every lncRNA-cancer association, a single representative publication is recorded. Finally, it is important to note that no lncRNAs were included based on evidence from previous driver-gene discovery studies of the types represented by OncodriveFML, ExInAtor, ncdDetect or others described in PCAWG^15,16,34,62^.

CLC set at this stage relies on GENCODE v24 annotation, and therefore all CLC genes have a GENCODE v24 ID assigned. However, data relative to GENCODE v24 was not available for all types of data and analyses used in this study (i.e. all data relative to PCAWG is based on GENCODE v19). Thus, for some analyses only genes also present in GENCODE v19 could be used (specified in the corresponding methods sections) and the total number of genes analyzed in these cases is slightly lower (107 instead of 122 CLC genes and 13,503 instead of 15,827 non-CLC).

### LncRNA and protein-coding driver prediction analysis

LncRNA and protein-coding predictions for ExInAtor and the rest of PCAWG methods, as well as the combined list of drivers, were extracted from the consortium database^16^. Parameters and details about each individual methods and the combined list of drivers can be found on the main PCAWG driver publication^16^ and false discovery rate correction was applied on each individual cancer type for each individual method in order to define candidates (*q*-value cutoffs of 0.1 and 0.2, specified in the corresponding sections). This way, we combined the predicted candidates of each individual method in each individual cancer type (including pan-cancer). To calculate sensitivity (percentage of true positives that are predicted as candidates) and precision (percentage of predicted candidates that are true positives) for lncRNA and protein-coding predictions we used the CLC and CGC (COSMIC v78, downloaded 3 October 2016) sets, respectively. To assess the statistical significance of sensitivity rates, we used Fisher’s exact test.

### Feature identification

We compiled several quantitative and qualitative traits of GENCODE lncRNAs and used them to compare CLC genes to the rest of lncRNAs (referred to as “non-CLC”). Analysis of quantitative traits were performed using Wilcoxon test while qualitative traits were tested using Fisher' exact test. These methods principally refer to Figs. 4 and 5 as well as Supplementary Figs. 4, 5, 6 and 7.

Cancer SNPs: On 4 October 2016, we collected all 2192 SNPs related to “cancer”, “tumour” and “tumor” terms in the NHGRI-EBI Catalog of published genome-wide association studies^63,64^ (https://www.ebi.ac.uk/gwas/home). Then we calculated the closest SNP to each lncRNA TSS using *closest* function from Bedtools v2.19^65^ (GENCODE v24).

Non-cancer SNPs: On 31 July 2017, we collected all 29,813 SNPs not related to “cancer”, “tumour” and “tumor” terms in the NHGRI-EBI Catalog of published genome-wide association studies^63,64^ (https://www.ebi.ac.uk/gwas/home). Then we calculated the closest SNP to each lncRNA TSS using *closest* function from Bedtools v2.19^65^ (GENCODE v24).

Epigenetically-silenced lncRNAs: We obtained a published list of 203 cancer-associated epigenetically-silenced lncRNA genes present in GENCODE v24^49^. These candidates were identified due to DNA methylation alterations in their promoter regions affecting their expression in several cancer types.

Differentially expressed in cancer: We collected a list of 3533 differentially expressed lncRNAs in cancer compared with normal samples^49^ (GENCODE v24).

Sequence/gene properties: Exonic positions of each gene were defined as the the union of exons from all its transcripts. Introns were defined as all remaining non-exonic nucleotides within the gene span. Repeats coverage refers to the percent of exonic nucleotides of a given gene overlapping repeats and low complexity DNA sequence regions obtained from RepeatMasker data housed in the UCSC Genome Browser^66^. Exonic content refers to the fraction of total gene span covered by exons. For this section we used GENCODE v19.

Evolutionary conservation: Two types of PhastCons conservation data were used: base-level scores and conserved elements. These data for different multispecies alignments (GRCh38/hg38) were downloaded from UCSC genome browser^66^. Mean scores and percent overlap by elements were calculated for exons and promoter regions (GENCODE v24). Promoters were defined as the 200 nt region centred on the annotated gene start.

Expression: We used polyA+RNA-seq data from 10 human cell lines produced by ENCODE^67,68^, from various human tissues by the Illumina Human Body Map Project (HBM) (www.illumina.com; ArrayExpress ID: E-MTAB-513), and from cancer samples from PCAWG RNA-seq expression data^16^. In this last case, for each cancer type we computed the expression mean of genes across all RNA-seq samples belonging to that cancer type (GENCODE v19).

Transposable elements: We downloaded 5,520,016 transposable elements from the UCSC table browser^69^ on 3 August 2017. We separated them by element types and counted how many of them intersected or not with the transcription start sites of CLC and non-CLC genes, in order to detect any association with the Fisher' exact test.

Distance to protein-coding genes and CGC genes: For each lncRNA we calculated the TSS to TSS distance to the closest protein-coding gene (GENCODE v24) or CGC gene (downloaded on 3 October 2016 from Cosmic database)^31^ using *closest* function from Bedtools v2.19^65^. In order to divide non-CLC genes into potentially functional non-CLC (PF-non-CLC) and others, we sampled the list of all non-CLC genes to get a subsample that has a matched distribution to CLC genes in conservation (% of conserved elements, from Vertebrate Multiz Alignment 100 Species from UCSC genome browser data, in exonic regions). Then we sampled again the resulting subset to get a final subset that also matches CLC genes in terms of expression (median of expression across 16 human tissues, data from Illumina Human Body Map Project (HBM)). To create the non-CLC samples we used the *matchDistribution* script: https://github.com/julienlag/matchDistribution.

Coexpression with closest CGC gene: We took CLC-CGC gene pairs whose TSS-TSS distance was <200 kb. RNA-seq data from 11 human cell lines from ENCODE was used to assess expression levels^67,68^. ENCODE RNA-seq data were obtained from ENCODE Data Coordination Centre (DCC) in September 2016, https://www.encodeproject.org/matrix/?type=Experiment. All data is relative to GENCODE v24. We calculated the expression correlation of gene pairs within each of the 11 cell lines, using the Pearson measure. To control for the effect of proximity, we randomly sampled a subset of non-CLC-CGC pairs matching the same TSS-TSS distance distribution as above, and performed the same expression correlation analysis (“non-CLC-CGC”). Finally, to further control for the fact that CLC and CGC are both cancer genes, which may influence their expression correlation, we shuffled CLC-CGC pairs 1000 times, and tested expression correlation for each set (“Shuffled CLC-CGC”).

Genomic classification: We used an in-house script (https://github.com/gold-lab/shared_scripts/tree/master/lncRNA.annotator) to classify lncRNA transcripts into different genomic categories based on their orientation and proximity to the closest protein-coding gene (GENCODE v24): a 10 kb distance was used to distinguish “genic” from “intergenic” lncRNAs. When transcripts belonging to the same gene had different classifications, we used the category represented by the largest number of transcripts.

Functional enrichment analysis: The list of protein-coding genes (GENCODE v24) that are divergent and closer than 10 kb to CLC genes (or non-CLC) was used for a functional enrichment analysis (20 unique genes in the case of CLC analysis and 1202 in the case of non-CLC analysis). We show data obtained using g:Profiler web server^70^, g:GOSt, with default parameters for functional enrichment analysis of protein-coding genes divergent to CLC and using Bonferroni correction for protein-coding gene divergent to non-CLC. For CLC analysis we performed the same test with independent methods: Metascape (http://metascape.org)^71^ and GeneOntoloy (Panther classification system)^72,73^. In both cases similar results were found.

### Mouse mutagenesis screen analysis

We extracted the genomic coordinates of transposon common insertion sites (CISs) in Mouse (GRCm38/mm10) http://ccgd-starrlab.oit.umn.edu/about.php56. This database contains target sites identified by transposon-based forward genetic screens in mice. LiftOver^61^ was used at default settings to obtain aligned human genome coordinates (hCISs) (GRCh38/hg38). We discarded hCIS regions longer than 1000 nucleotides for all the analyses; and also those that overlap protein-coding genes (except for Fig. 6b). The remainders (90 hCISs) were intersected with the genomic coordinates of CLC and non-CLC genes that do not overlap protein-coding genes.

To correctly assess the statistical enrichment of CLC in hCIS regions, we performed two control analyses:

Length-matched sampling: To calculate if the enrichment of hCIS intersecting genes in CLC set is higher and statistically different from non-CLC set, while controlling by gene length, we created 1000 samples of non-CLC genes with the same gene length distribution as CLC genes. Each sample was intersected with hCIS, and the number of intersecting hCISs per Mb of gene length was calculated. To create the non-CLC samples we used the *matchDistribution* script: https://github.com/julienlag/matchDistribution. Finally, we calculated an empirical *p*-value by counting how many of the simulated non-CLC enrichments were higher or equal than the real CLC value.

Randomly repositioning of CLC and non-CLC genes: We randomly relocated CLC/non-CLC genes 10,000 times within the non-protein-coding regions of the genome using the tool *shuffle* from BedTools v19^65^. In each iteration, we calculated the number of genes that intersected at least one hCIS, and created the distribution of these simulated values. Finally, we calculated an empirical *p*-value by counting how many of the simulated values were higher or equal than the real values. This analysis was performed separately for CLC and non-CLC genes.

### Reporting summary

Further information on research design is available in the Nature Research Reporting Summary linked to this article.

## Supplementary information

Supplementary Information

Description of additional supplementary items

Reporting Summary

## Data Availability

The data reported in this study are summarized in the manuscript and its Supporting Information files. The list of CLC genes are also available from the GOLD Lab website (https://www.gold-lab.org/clc). Somatic and germline variant calls, mutational signatures, subclonal reconstructions, transcript abundance, splice calls and other core data generated by the ICGC/TCGA Pan-cancer Analysis of Whole Genomes Consortium is described here^38^ and available for download at https://dcc.icgc.org/releases/PCAWG. Additional information on accessing the data, including raw read files, can be found at https://docs.icgc.org/pcawg/data/. In accordance with the data access policies of the ICGC and TCGA projects, most molecular, clinical and specimen data are in an open tier which does not require access approval. To access potentially identification information, such as germline alleles and underlying sequencing data, researchers will need to apply to the TCGA Data Access Committee (DAC) via dbGaP (https://dbgap.ncbi.nlm.nih.gov/aa/wga.cgi?page=login) for access to the TCGA portion of the dataset, and to the ICGC Data Access Compliance Office (DACO; http://icgc.org/daco) for the ICGC portion. In addition, to access somatic single nucleotide variants derived from TCGA donors, researchers will also need to obtain dbGaP authorisation.

## Code availability

Custom code are available from the corresponding author upon request. The core computational pipelines used by the PCAWG Consortium for alignment, quality control and variant calling are available to the public at https://dockstore.org/search?search=pcawg under the GNU General Public License v3.0, which allows for reuse and distribution.
