# Supplementary material for: Cancer LncRNA Census reveals evidence for deep functional conservation of long noncoding RNAs in tumorigenesis
Source: Commun Biol. 2020 Feb 5;3:56. doi: 10.1038/s42003-019-0741-7 (PMC7002399; doi:10.1038/s42003-019-0741-7)
Supplement: Supplementary file 1 — Supplementary Information [file 42003_2019_741_MOESM1_ESM.pdf]

Supplementary Figure 1

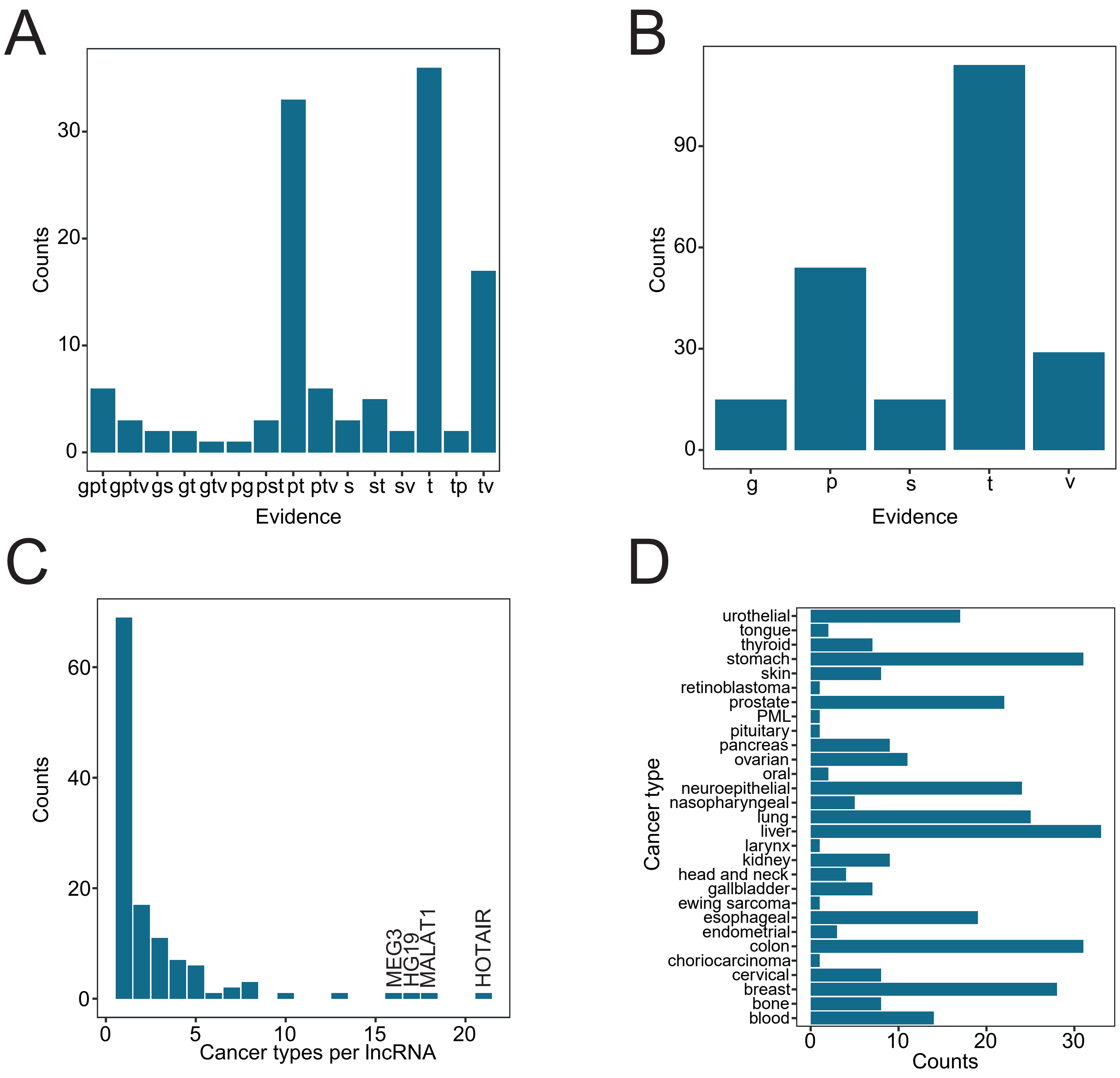

Supplementary Figure 1: CLC summary statistics. (A) Barplot showing the non-redundant number of genes in CLC broken down by supporting evidence types. p: prognostic; t: in vitro; v: in vivo; g: germline mutations; s: somatic mutations. (B) Similar as previous, but with redundant number of genes per individual evidence type. (C) Histogram of genes broken down by their number of associated cancer types. (D) Histogram of cancer types, by their redundant number of associated lncRNAs.

Supplementary Figure 2

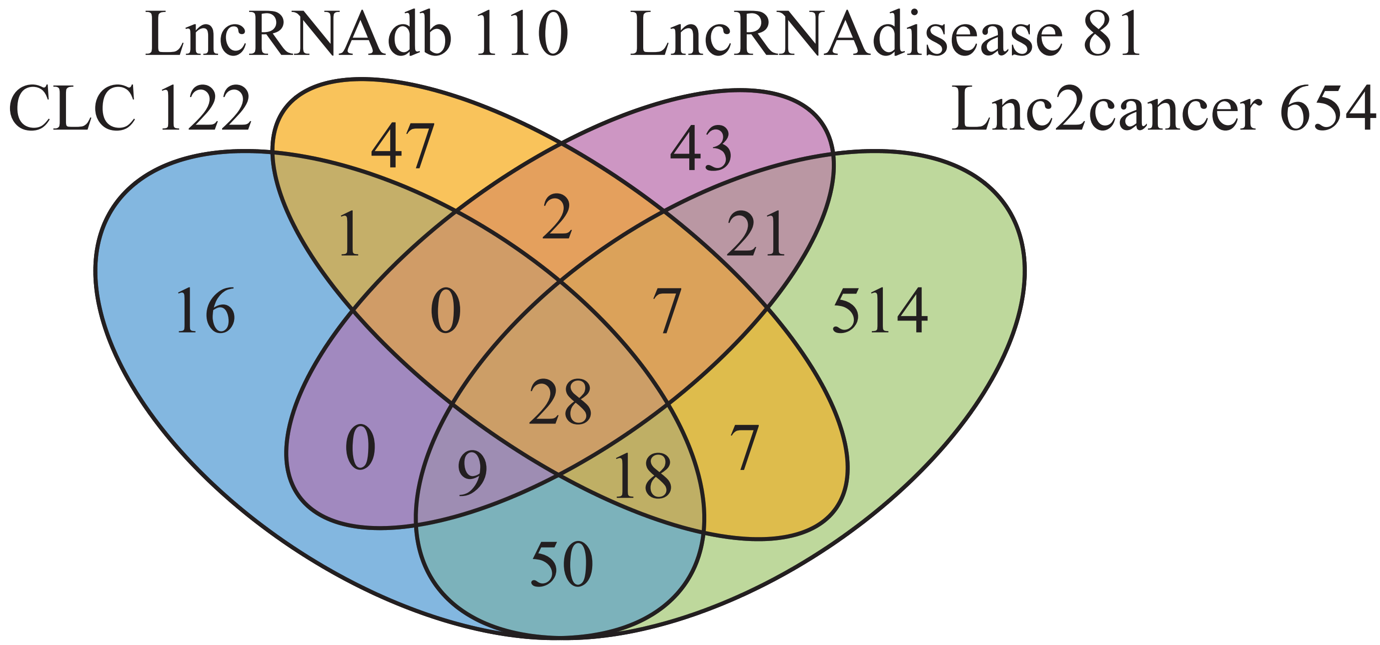

Supplementary Figure 2: Intersection between cancer lncRNA databases. Venn diagram representing the number of GENCODE genes that are present in each of the four databases and their intersections. Numbers next to databases names correspond to the total number of included GENCODE genes.

Supplementary Figure 3

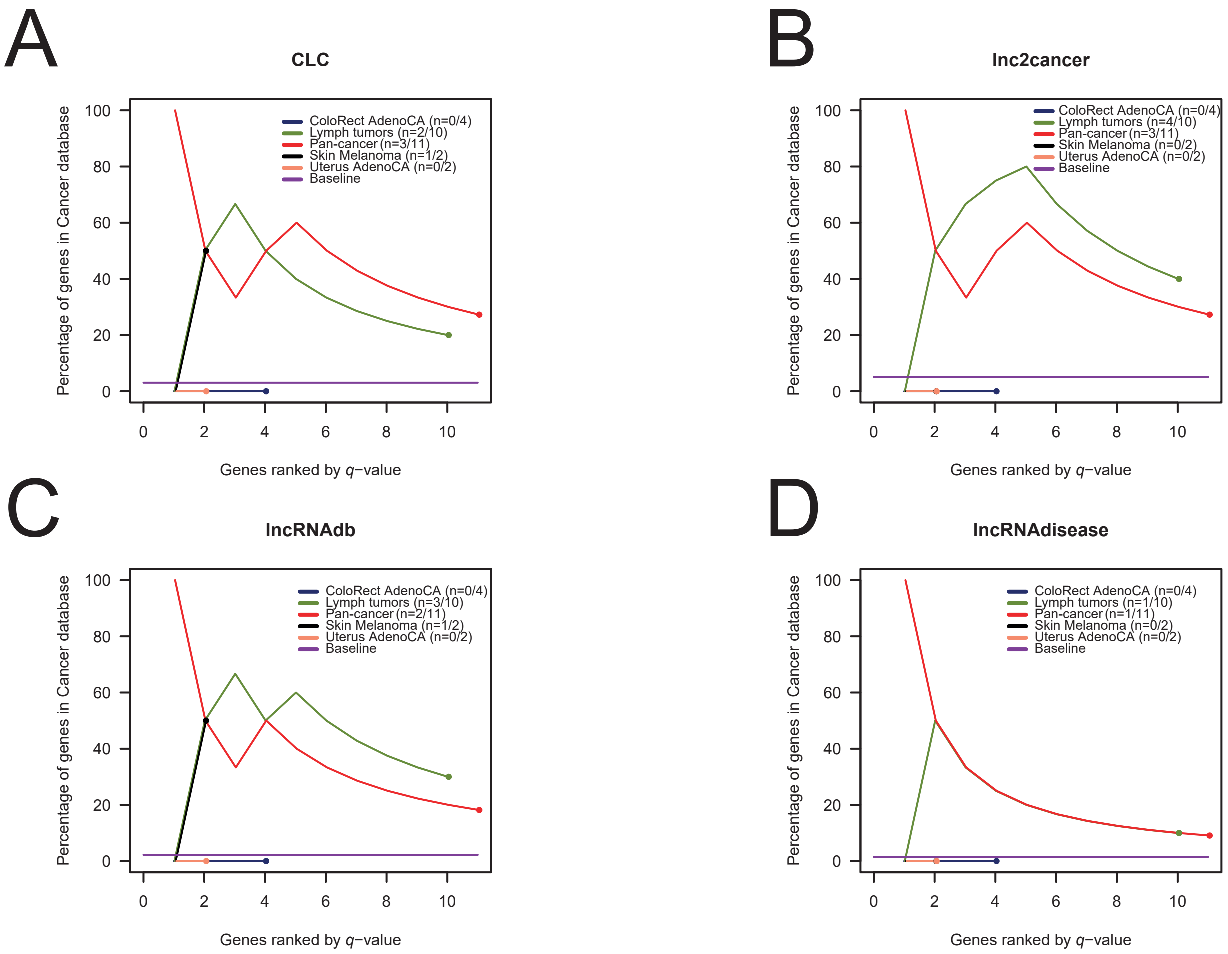

Supplementary Figure 3: Benchmarking of ExInAtor driver lncRNA predictions for four cancer databases. (A) CLC, (B) lnc2cancer, (C) lncRNAdb and (D) lncRNAdisease. PCAWG whole genome tumours candidates at *q*-value (false discovery rate) cutoff of 0.2 sorted increasingly by *q*-value are ranked on *x*-axis. Percentage of genes from each database amongst cumulative set of predicted candidates at each step of the ranking (precision), are shown on the *y*-axis. Black line shows the baseline, being the percentage of genes from each database in the whole list of genes tested. Coloured dots represent the number of candidates predicted under the *q*-value cutoff of 0.2. “n” in the legend shows the number of genes in each database and total candidates for each cancer type.

Supplementary Figure 4

A

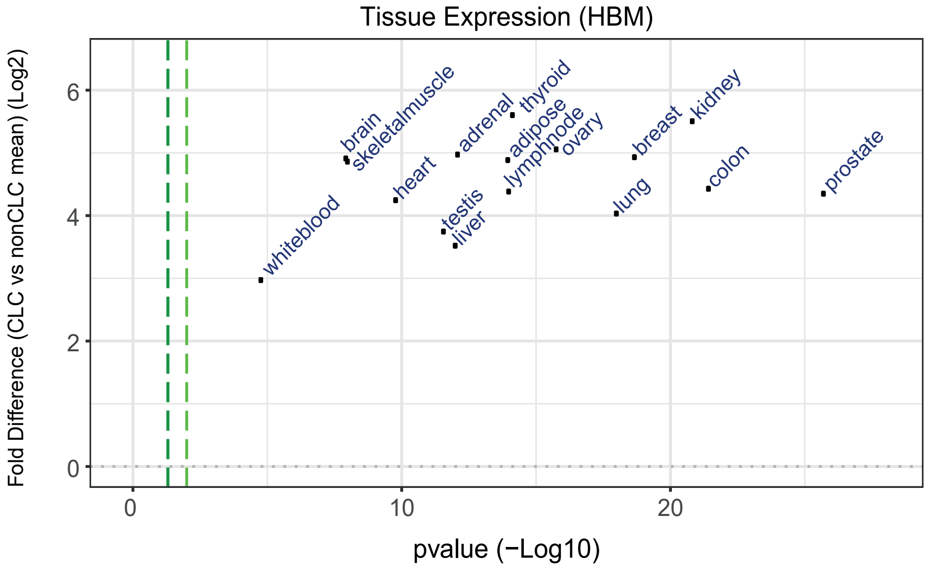

B

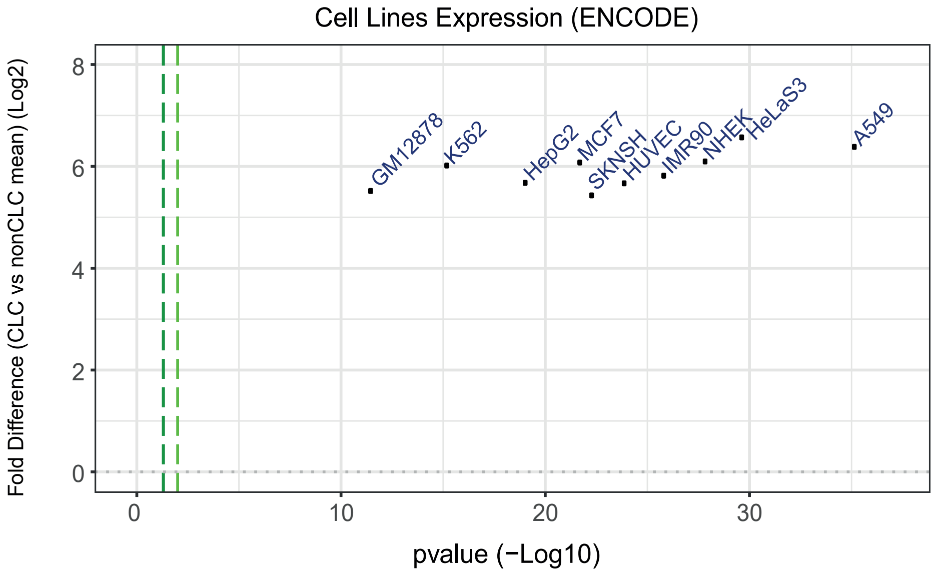

Supplementary Figure 4: CLC genes are highly expressed. Panels display feature analysis results similar to Figure 4 using other datasets. (A) Panel displaying the log fold difference between CLC and nonCLC genesets (*y*-axis) and statistical significance by Wilcoxon test (*x*-axis) when comparing RNAseq expression levels in human tissues (each dot represents a different tissue) from Human Body Map data. (B) Same than previous panel for expression data in human cell lines instead of tissues, from ENCODE RNAseq data.

Supplementary Figure 5

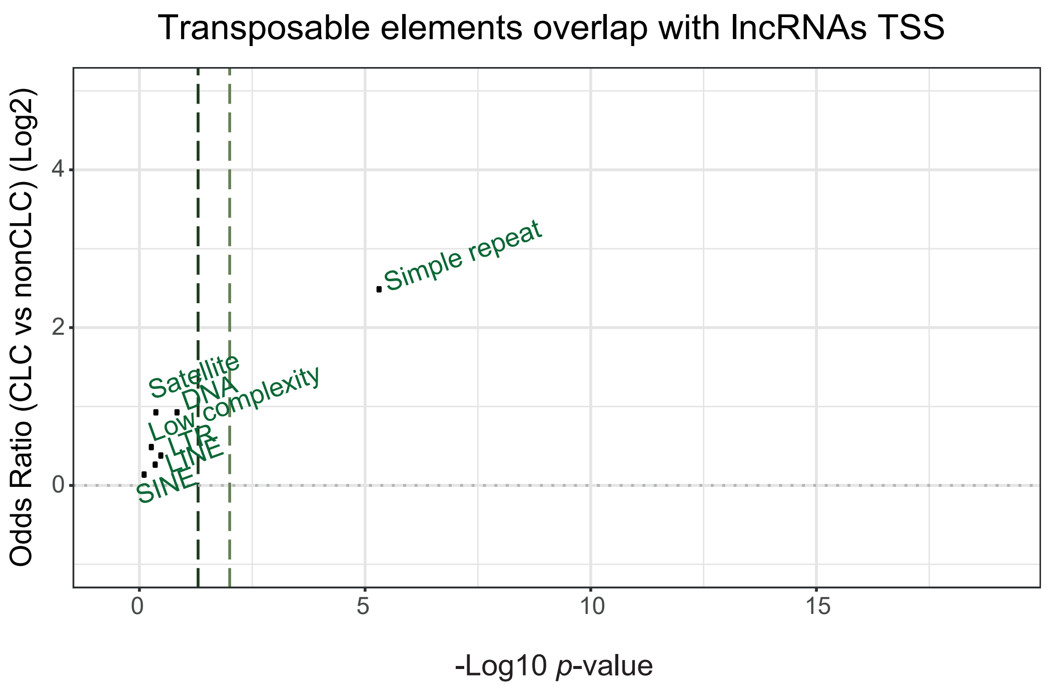

Supplementary Figure 5: CLC TSSs association with Transposable Elements. Figure shows the comparison of the intersection of each category of Transposable elements with transcription start sites (TSS) of CLC and nonCLC genes. *y*-axis shows the log2 of the Odds Ratio obtained by comparing CLC to nonCLC by Fisher's exact test; *x*-axis displays the estimated *p*-value from the same test.

Supplementary Figure 6

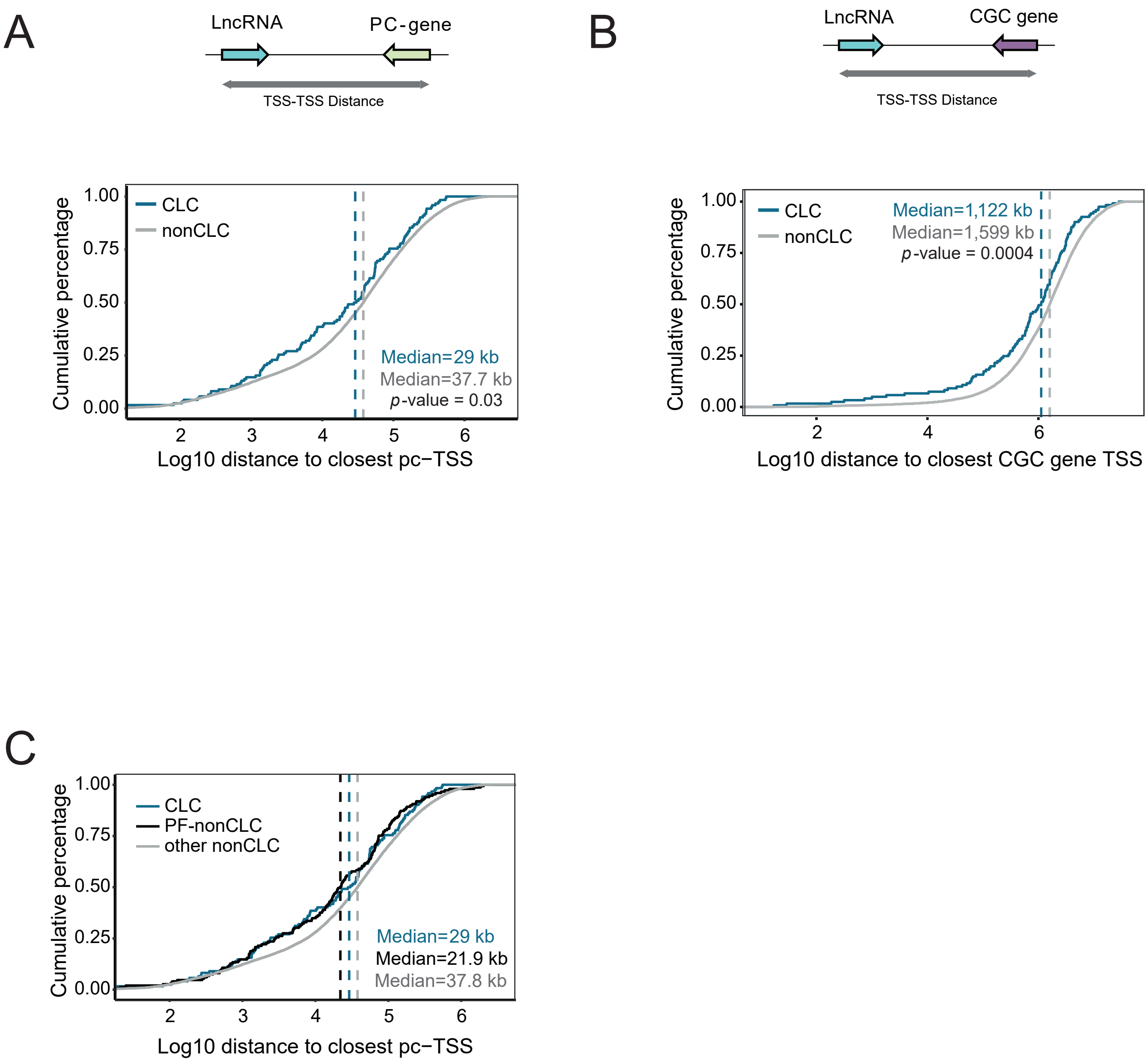

Supplementary Figure 6: CLC lncRNAs tend to be closer to protein-coding genes. (A) Cumulative distribution of the genomic distance from CLC and nonCLC genes, to the closest protein-coding gene (NB this may be a CGC gene or not). Distances are defined as the distance of the annotated transcription start site (TSS) of each gene in the pair. *p*-value for Wilcoxon test is shown. (B) Same as (A) for genomic distance to closest CGC genes. (C) Same than A dividing nonCLC genes into two groups: potentially functional nonCLC (PF-nonCLC) (those nonCLC genes that are in the same range of expression and conservation than CLC genes) and other nonCLC (the rest of nonCLC genes).

Supplementary Figure 7

A

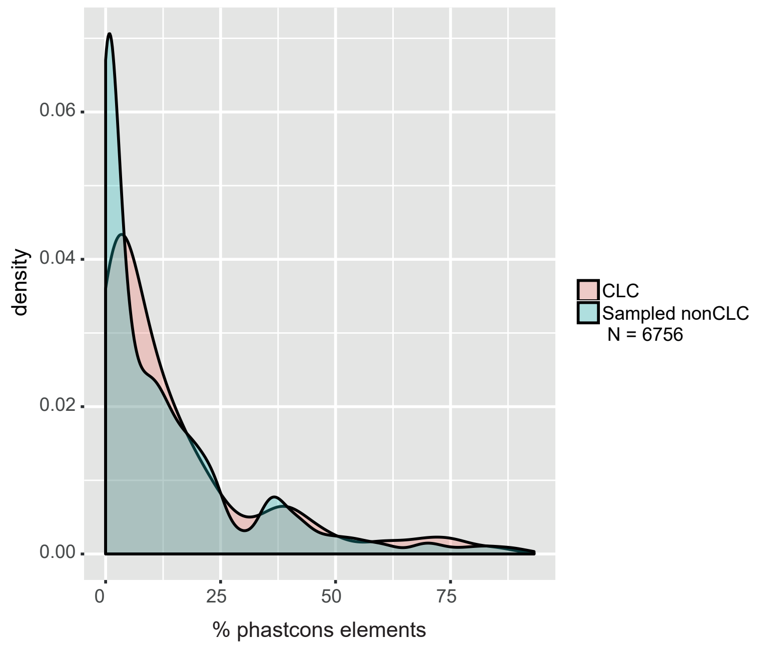

B

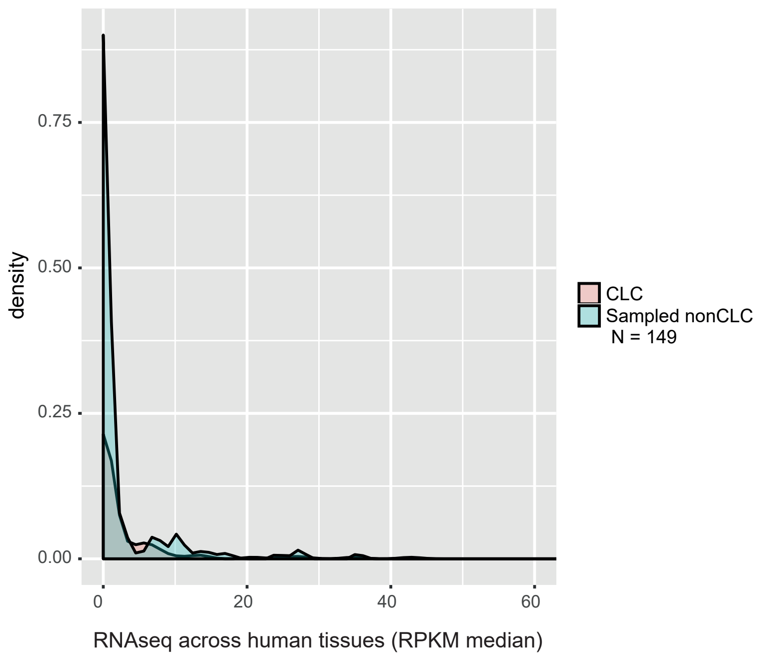

Supplementary Figure 7: sampling nonCLC genes. (A) Density plot comparing the percentage of PhastCons conserved elements in lncRNA exons of CLC genes and a subset of nonCLC sampled to match CLC conservation distribution. (B) Same than A but comparing the median of RNAseq expression values across 16 human tissues. NonCLC subset here is sampled from the subset obtained after matching conservation distribution.

Supplementary Figure 8

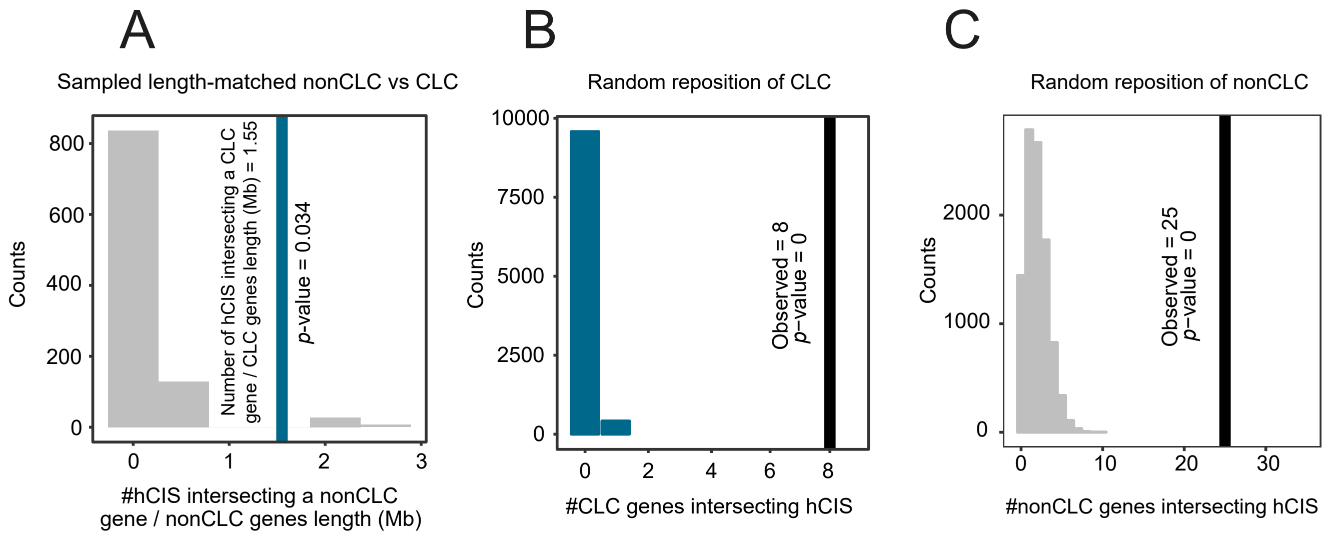

Supplementary Figure 8: hCIS enrichment corrected by gene length. Nucleotides intersecting protein-coding genes were subtracted from the genomic regions in the analyses shown here. (A) Distribution of the number of intersecting hCIS per Megabase (Mb) of total gene length, for 1000 subsets of nonCLC genes with same length distribution as CLC genes (grey). Vertical blue line represents the overall value for CLC geneset: 1.55 hCIS sites per Mb of gene span. (B) Distribution of the number of genes overlapping a hCIS after 10,000 genomic randomizations of CLC genes (blue). Vertical black line represents the observed number of CLC genes (8) that intersect a hCIS. (C) Distribution of the number of intersecting genes with a hCIS after 10,000 genomic randomizations of nonCLC genes (grey). Vertical black line represents the observed number of nonCLC genes that intersect a hCIS (25).

Supplementary Note:

TCGA/ICGC Pan-Cancer Analysis of Whole Genomes Network

Steering committee

Peter J Campbell#[1,2], **Gad Getz#**[3,4,5,6], **Jan O Korbel#**[7,8], **Lincoln D Stein#**[9,10] and **Joshua M Stuart#**[11]

Executive committee

Sultan T Al-Sedairy[12], Axel Aretz[13], Cindy Bell[14], Miguel Betancourt[15], Christiane Buchholz[16], Fabien Calvo[17], Christine Chomienne[18], Michael Dunn[19], Stuart Edmonds[20], Eric Green[21], Shailja Gupta[22], Carolyn M Hutter[21], Karine Jegalian[23], Jennifer L Jennings[24,25], Nic Jones[26], Hyung-Lae Kim[27], Youyong Lu[28,29,30], Hitoshi Nakagama[31], Gerd Nettekoven[32], Laura Planko[32], David Scott[26], Tatsuhiro Shibata[33,34], Kiyo Shimizu[35], Lincoln D Stein[9,10], Michael Rudolf Stratton[2], Takashi Yugawa[36], Giampaolo Tortora[37,38], K VijayRaghavan[22], Huanming Yang[39] and Jean C Zenklusen[40]

Ethics and legal working group

Don Chalmers#[41], Yann Joly[42], **Bartha M Knoppers#**[42], Fruzsina Molnár-Gábor[43], Mark Phillips[42], Adrian Thorogood[42] and David Townend[44]

Technical working group

Brice Aminou[45], Javier Bartolome[46], Keith A Boroevich[47,48], Rich Boyce[7], Angela N Brooks[3,49,50], Alex Buchanan[51], Ivo Buchhalter[52,53,54], Adam P Butler[2], Niall J Byrne[45], Andy Cafferkey[7], Peter J Campbell[1,2], Zhaohong Chen[55], Sunghoon Cho[56], Wan Choi[57], Peter Clapham[2], Brandi N Davis-Dusenbery[58], Francisco M De La Vega[59,59,60,61,62], Jonas Demeulemeester[63,64], Michelle T Dow[55], Lewis Jonathan Dursi[9,65], Juergen Eils[66,67], Roland Eils[52,54,66,67], Kyle Ellrott[51], Claudiu Farcas[55], Francesco Favero[68], Nodirjon Fayzullaev[45], Vincent Ferretti[45,69], Paul Flicek[7], Nuno A Fonseca[7,70], Josep Ll Gelpi[46,71], Gad Getz[3,4,5,6], Bob Gibson[45], Robert L Grossman[72], Olivier Harismendy[73], Allison P Heath[74], Michael C Heinold[52,54], Julian M Hess[3,75], Oliver Hofmann[76], Jongwhi H Hong[77], Thomas J Hudson[78,79], Barbara Hutter[80,81,82], Carolyn M Hutter[21], Daniel Hübschmann[54,66,83,84,85], Seiya Imoto[86,87], Sinisa Ivkovic[88], Seung-Hyup Jeon[57], Wei Jiao[9], Jongsun Jung[89], Rolf Kabbe[52], Andre Kahles[90,91,92,93,94], Jules NA Kerssemakers[52], Hyung-Lae Kim[27], Hyunghwan Kim[57], Jihoon Kim[95], Youngwook Kim[96,97], Kortine Kleinheinz[52,54], Jan O Korbel[7,8], Michael Koscher[98], Antonios Koures[55], Milena Kovacevic[88], Chris Lawerenz[67], Ignaty Leshchiner[3], Jia Liu[99], Dimitri Livitz[3], George L Mihaiescu[45], Sanja Mijalkovic[88], Ana Mijalkovic Mijalkovic-Lazic[88], Satoru Miyano[87], Naoki Miyoshi[87], Hardeep K Nahal-Bose[45], Hidewaki Nakagawa[48], Mia Nastic[88], Steven J Newhouse[7], Jonathan Nicholson[2], **Brian D O'Connor#**[45,50], David Ocana[7], Kazuhiro Ohi[86], Lucila

Ohno-Machado[55], Larsson Omberg[100], BF Francis Ouellette[101,102], Nagarajan Paramasivam[52,81], Marc D Perry[45,103], Todd D Pihl[104], Manuel Prinz[52], Montserrat Puiggròs[105], Petar Radovic[88], Keiran M Raine[2], Esther Rheinbay[3,6,106], Mara Rosenberg[3,106], Romina Royo[105], Gunnar Rätsch[90,93,94,107,108,109], Gordon Saksena[3], Matthias Schlesner[52,110], Solomon I Shorser[9], Charles Short[7], Heidi J Sofia[21], Jonathan Spring[72], **Lincoln D Stein**#[9,10], Adam J Struck[51], Grace Tiao[3], Nebojsa Tijanic[88], David Torrents[105,111], Peter Van Loo[63,64], Miguel Vazquez[105,112], David Vicente[105], Jeremiah A Wala[3,6,49], Zhining Wang[40], Sebastian M Waszak[8], Joachim Weischenfeldt[8,113,114], Johannes Werner[52,115], Ashley Williams[55], Youngchoon Woo[57], Adam J Wright[9], Qian Xiang[116], **Sergei Yakneen**#[8], Liming Yang[40], Denis Yuen[9], **Christina K Yung**#[45] and **Junjun Zhang**#[45]

Annotations working group

Angela N Brooks#[3,49,50], Ivo Buchhalter[52,53,54], Peter J Campbell[1,2], Priyanka Dhingra[117,118], Lars Feuerbach[119], Mark Gerstein[120,121,122,123], Gad Getz[3,4,5,6], Mark P Hamilton[124], Henrik Hornshøj[125], Todd A Johnson[47], Andre Kahles[90,91,92,93,94], Abdullah Kahraman[126,127,128], Manolis Kellis[3,129], **Ekta Khurana**#[117,118,130,131], Jan O Korbel[7,8], Morten Muhlig Nielsen[125], Jakob Skou Pedersen[125,132], Paz Polak[3,4,6], Jüri Reimand[9,133], Esther Rheinbay[3,6,106], Nicola D Roberts[2], Gunnar Rätsch[90,93,94,107,108,109], Richard Sallari[3], Nasa Sinnott-Armstrong[3,61], Alfonso Valencia[105,111], Miguel Vazquez[105,112], Sebastian M Waszak[8], Joachim Weischenfeldt[8,113,114] and Christian von Mering[128,134]

Quality control working group

Sergi Beltran[135,136], Ivo Buchhalter[52,53,54], Peter J Campbell[1,2], Roland Eils[52,54,66,67], Daniela S Gerhard[137], Gad Getz[3,4,5,6], **Ivo G Gut**#[135,136], Marta Gut[135,136], Barbara Hutter[80,81,82], Daniel Hübschmann[54,66,83,84,85], Kortine Kleinheinz[52,54], Jan O Korbel[7,8], Dimitri Livitz[3], Marc D Perry[45,103], Keiran M Raine[2], Esther Rheinbay[3,6,106], Mara Rosenberg[3,106], Gordon Saksena[3], Matthias Schlesner[52,110], Miranda D Stobbe[135,136], Jean-Rémi Trotta[135], Johannes Werner[52,115] and Justin P Whalley[135]

Novel somatic mutation calling methods

Matthew H Bailey[138,139], Beifang Niu[140], Matthias Bieg[81,141], Paul C Boutros[9,133,142,143], Ivo Buchhalter[52,53,54], Adam P Butler[2], Ken Chen[144], Zechen Chong[145], **Li Ding**#[138,139,146], Oliver Drechsel[136,147], Lewis Jonathan Dursi[9,65], Roland Eils[52,54,66,67], Kyle Ellrott[51], Shadrielle MG Espiritu[9], Yu Fan[148], Robert S Fulton[138,139,146], Shengjie Gao[149], Josep Ll Gelpi[46,71], Mark Gerstein[120,121,122,123], Gad Getz[3,4,5,6], Santiago Gonzalez[7,8], Ivo G Gut[135,136], Faraz Hach[150,151], Michael C Heinold[52,54], Julian M Hess[3,75], Jonathan Hinton[2], Taobo Hu[152], Vincent Huang[9], Yi Huang[153,154], Barbara Hutter[80,81,82], David R Jones[2], Jongsun Jung[89], Natalie Jäger[52], Hyung-Lae Kim[27], Kortine Kleinheinz[52,54], Sushant Kumar[122,123], Yogesh Kumar[152], Christopher M Lalansingh[9], Ignaty Leshchiner[3], Ivica Letunic[155], Dimitri Livitz[3], Eric Z Ma[152], Yosef E

Maruvka[3,75,106], R Jay Mashl[139,156], Michael D McLellan[138,139,146], Andrew Menzies[2], Ana Milovanovic[46], Morten Muhlig Nielsen[125], Stephan Ossowski[136,147,157], Nagarajan Paramasivam[52,81], Jakob Skou Pedersen[125,132], Marc D Perry[45,103], Montserrat Puiggròs[105], Keiran M Raine[2], Esther Rheinbay[3,6,106], Romina Royo[105], S Cenk Sahinalp[151,158,159], Gordon Saksena[3], Iman Sarrafi[151,159], Matthias Schlesner[52,110], **Jared T Simpson#**[9,160], Lucy Stebbings[2], Chip Stewart[3], Miranda D Stobbe[135,136], Jon W Teague[2], Grace Tiao[3], David Torrents[105,111], Jeremiah A Wala[3,6,49], Jiayin Wang[139,154,161], Wenyi Wang[148], Sebastian M Waszak[8], Joachim Weischenfeldt[8,113,114], Michael C Wendl[139,162,163], Johannes Werner[52,115], David A Wheeler[164,165], Zhenggang Wu[152], Hong Xue[152], Sergei Yakneen[8], Takafumi N Yamaguchi[9], Kai Ye[161,166], Venkata D Yellapantula[167,168], Christina K Yung[45] and Junjun Zhang[45]

Drivers and functional interpretation

Federico Abascal[2], Samirkumar B Amin[169,170,171], Gary D Bader[10], Jonathan Barenboim[9], Rameen Beroukhim[3,6,172], Johanna Bertl[125,173], Keith A Boroevich[47,48], Søren Brunak[174,175], Peter J Campbell[1,2], Joana Carlevaro-Fita[176,177,178], Dimple Chakravarty[179], Calvin Wing Yiu Chan[52,180], Ken Chen[144], Jung Kyoon Choi[181], Jordi Deu-Pons[182,183], Priyanka Dhingra[117,118], Klev Diamanti[184], Lars Feuerbach[119], J Lynn Fink[105,185], Nuno A Fonseca[7,70], Joan Frigola[182], Carlo Gambacorti-Passerini[186], Dale W Garsed[187,188], **Mark Gerstein#**[120,121,122,123], **Gad Getz#**[3,4,5,6], Abel Gonzalez-Perez[183,189,190], Qianyun Guo[132], Ivo G Gut[135,136], David Haan[11], Mark P Hamilton[124], Nicholas J Haradhvala[3,106], Arif O Harmanci[123,191], Mohamed Helmy[192], Carl Herrmann[52,54,193], Julian M Hess[3,75], Asger Hobolth[132,173], Ermin Hodzic[159], Chen Hong[119,180], Henrik Hornshøj[125], Keren Isaev[9,133], Jose MG Izarzugaza[174], Rory Johnson[177,194], Todd A Johnson[47], Malene Juul[125], Randi Istrup Juul[125], Andre Kahles[90,91,92,93,94], Abdullah Kahraman[126,127,128], Manolis Kellis[3,129], Ekta Khurana[117,118,130,131], Jaegil Kim[3], Jong K Kim[195], Youngwook Kim[96,97], Jan Komorowski[184,196], Jan O Korbel[7,8], Sushant Kumar[122,123], Andrés Lanzós[177,178,194], **Erik Larsson#**[90], **Michael S Lawrence#**[3,47,106], Donghoon Lee[123], Kjong-Van Lehmann[90,92,93,94,197], Shantao Li[123], Xiaotong Li[123], Ziao Lin[3,198], Eric Minwei Liu[117,118,199], Lucas Lochovsky[170,200,201,202], Shaoke Lou[122,123], Tobias Madsen[125], Kathleen Marchal[203,204], Iñigo Martincorena[2], Alexander Martinez-Fundichely[117,118,130], Yosef E Maruvka[3,75,106], Patrick D McGillivray[122], William Meyerson[123,205], Ferran Muiños[183,190], Loris Mularoni[183,190], Hidewaki Nakagawa[48], Morten Muhlig Nielsen[125], Marta Paczkowska[9], Keunchil Park[206,207], Kiejung Park[208], **Jakob Skou Pedersen#**[125,132], Oriol Pich[183,190], Tirso Pons[209], Sergio Pulido-Tamayo[203,204], **Benjamin J Raphael#**[120], Jüri Reimand[9,133], Iker Reyes-Salazar[190], Matthew A Reyna[120], Esther Rheinbay[3,6,106], Mark A Rubin[131,194,210,211,212], Carlota Rubio-Perez[183,190,213], Radhakrishnan Sabarinathan[183,190,214], S Cenk Sahinalp[151,158,159], Gordon Saksena[3], Leonidas Salichos[122,123], Chris Sander[49,90,215,216], Steven E Schumacher[3,217], Mark Shackleton[188,218], Ofer Shapira[3,49], Ciyue Shen[216,219], Raunak Shrestha[151], Shimin Shuai[9,10], Nikos Sidiropoulos[113], Lina Sieverling[119,180], Nasa Sinnott-Armstrong[3,61], Lincoln D Stein[9,10], **Joshua M Stuart#**[11], David Tamborero[183,190], Grace Tiao[3], Tatsuhiko Tsunoda[47,220,221,222], Husen M Umer[184,223], Liis Uusküla-Reimand[224,225], Alfonso Valencia[105,111], Miguel Vazquez[105,112], Lieven PC Verbeke[204,226], Claes Wadelius[227], Lina Wadi[9], Jiayin Wang[139,154,161], Jonathan Warrell[122,123], Sebastian M Waszak[8], Joachim Weischenfeldt[8,113,114], **David A Wheeler#**[164,165], Guanming Wu[228], Jun Yu[229], [230], Jing Zhang[123], Xuanping Zhang[154,231], Yan Zhang[123,232,233], Zhongming Zhao[234], Lihua

Zou[235] and Christian von Mering[128,134]

Integration of transcriptome and genome

Samirkumar B Amin[169,170,171], Philip Awadalla[9,10], Peter J Bailey[236], **Alvis Brazma#**[7], **Angela N Brooks#**[3,49,50], Claudia Calabrese[7,8], Aurélien Chateigner[45], Isidro Cortés-Ciriano[237,238,239], Brian Craft[240], David Craft[3], Chad J Creighton[241], Natalie R Davidson[90,92,93,109,197], Deniz Demircioğlu[242,243], Serap Erkek[8], Nuno A Fonseca[7,70], Milana Frenkel-Morgenstern[244], Mary J Goldman[240], Liliana Greger[7], Jonathan Göke[242,245], Yao He[246], Katherine A Hoadley[247,248], Yong Hou[39,249], Matthew R Huska[250], Andre Kahles[90,91,92,93,94], Ekta Khurana[117,118,130,131], Helena Kilpinen[251], Jan O Korbel[7,8], Fabien C Lamaze[9], Kjong-Van Lehmann[90,92,93,94,197], Chang Li[39,249], Siliang Li[39,249], Xiaobo Li[39,249], Xinyue Li[39], Dongbing Liu[39,249], Fenglin Liu[246,252], Xingmin Liu[39,249], Maximillian G Marin[50], Julia Markowski[250], Matthew Meyerson[3,6,49,177,253], Tannistha Nandi[254], Morten Muhlig Nielsen[125], Akinyemi I Ojesina[255,256,257], BF Francis Ouellette[101,102], Qiang Pan-Hammarström[39,258], Peter J Park[238,259], Chandra Sekhar Pedamallu[3,6,172], Jakob Skou Pedersen[125,132], Marc D Perry[45,103], **Gunnar Rätsch#**[90,93,94,107,108,109], Roland F Schwarz[7,84,250,260], Yuichi Shiraishi[87], Reiner Siebert[261,262], Cameron M Soulette[50], Stefan G Stark[93,197,263,264], Oliver Stegle[7,8,265], Hong Su[39,249], Patrick Tan[254,266,267,268], Bin Tean Teh[266,267,268,269,270], Lara Urban[7,8], Jian Wang[39], Sebastian M Waszak[8], Kui Wu[39,249], Qian Xiang[116], Heng Xiong[39,249], Sergei Yakneen[8], Huanming Yang[39], Chen Ye[39,249], Christina K Yung[45], Fan Zhang[246], Junjun Zhang[45], Xiuqing Zhang[39], Zemin Zhang[246,271], Liangtao Zheng[246], Jingchun Zhu[240] and Shida Zhu[39,249]

Integration of epigenome and genome

Hiroyuki Aburatani[272], **Benjamin P Berman#**[273,274,275], Hans Binder[276,277], **Benedikt Brors#**[82,119,278], Huy Q Dinh[273], Lars Feuerbach[119], Shengjie Gao[149], Ivo G Gut[135,136], Simon C Heath[135,136], Steve Hoffmann[277,279,280,281], Charles D Imbusch[119], Ekta Khurana[117,118,130,131], Helene Kretzmer[277,281], Peter W Laird[282], Jose I Martin-Subero[111,283], Genta Nagae[272,284], **Christoph Plass#**[285], Paz Polak[3,4,6], Hui Shen[286], Reiner Siebert[261,262], Nasa Sinnott-Armstrong[3,61], Miranda D Stobbe[135,136], Qi Wang[98], Dieter Weichenhan[285], Sergei Yakneen[8] and Wanding Zhou[286]

Patterns of structural variations, signatures, genomic correlations, retrotransposons, mobile elements

Kadir C Akdemir[144], Eva G Alvarez[287,288,289], Adrian Baez-Ortega[290], **Rameen Beroukhim#**[3,6,172], Paul C Boutros[9,133,142,143], David D L Bowtell[187,291], Benedikt Brors[82,119,278], Kathleen H Burns[292], **Peter J Campbell#**[1,2], Kin Chan[293], Ken Chen[144], Isidro Cortés-Ciriano[237,238,239], Ana Dueso-Barroso[46], Andrew J Dunford[3], Paul A Edwards[294,295], Xavier Estivill[296], Dariush Etemadmoghadam[187,188], Lars Feuerbach[119], J Lynn Fink[105,185], Milana Frenkel-Morgenstern[244], Dale W Garsed[187,188], Mark Gerstein[120,121,122,123], Dmitry A Gordenin[297], David Haan[11], James E Haber[298], Julian M Hess[3,75], Barbara Hutter[80,81,82], Marcin Imielinski[299,300], David TW Jones[301,302],

Young Seok Ju[2,181], Marat D Kazanov[303,304,305], Leszek J Klimczak[306], Youngil Koh[307,308], Jan O Korbel[7,8], Kiran Kumar[3], Eunjung Alice Lee[309], Jake June-Koo Lee[238,259], Yilong Li[2], Andy G Lynch[294,295,310], Geoff Macintyre[294], Florian Markowetz[294,295], Iñigo Martincorena[2], Alexander Martinez-Fundichely[117,118,130], Matthew Meyerson[3,6,49,177,253], Satoru Miyano[87], Hidewaki Nakagawa[48], Fabio CP Navarro[122], Stephan Ossowski[136,147,157], Peter J Park[238,259], John V Pearson[311,312], Montserrat Puiggròs[105], Karsten Rippe[84], Nicola D Roberts[2], Steven A Roberts[313], Bernardo Rodriguez-Martin[287,288,289], Steven E Schumacher[3,217], Ralph Scully[314], Mark Shackleton[188,218], Nikos Sidiropoulos[113], Lina Sieverling[119,180], Chip Stewart[3], David Torrents[105,111], Jose MC Tubio[287,288,289], Izar Villasante[105], Nicola Waddell[311,312], Jeremiah A Wala[3,6,49], Joachim Weischenfeldt[8,113,114], Lixing Yang[315], Xiaotong Yao[299,316], Sung-Soo Yoon[308], Jorge Zamora[2,287,288,289] and Cheng-Zhong Zhang[3,6,49]

Mutation signatures and processes

Ludmil B Alexandrov[2,317], Erik N Bergstrom[318], Arnoud Boot[267,319], Paul C Boutros[9,133,142,143], Kin Chan[293], Kyle Covington[165], Akihiro Fujimoto[48], Gad Getz[3,4,5,6], Dmitry A Gordenin[297], Nicholas J Haradhvala[3,106], Mi Ni Huang[267,319], S. M. Ashiqul Islam[317], Marat D Kazanov[303,304,305], Jaegil Kim[3], Leszek J Klimczak[306], Michael S Lawrence[3,47,106], Iñigo Martincorena[2], John R McPherson[267,319], Sandro Morganella[2], Ville Mustonen[320,321,322], Hidewaki Nakagawa[48], Avlin Wei Tian Ng[323], Serena Nik-Zainal[2,324,325,326], Paz Polak[3,4,6], Stephenie D Prokopec[9], Steven A Roberts[313], **Steven G Rozen**#[267,268,319], Radhakrishnan Sabarinathan[183,190,214], Natalie Saini[297], Tatsuhiro Shibata[33,34], Yuichi Shiraishi[87], **Michael Rudolf Stratton**#[2], **Bin Tean Teh**#[266,267,268,269,270], Ignacio Vázquez-García[2,167,327,328], Yang Wu[267,319], Fouad Yousif[9] and Willie Yu[329]

Germline cancer genome

Ludmil B Alexandrov[2,317], Eva G Alvarez[287,288,289], Adrian Baez-Ortega[290], Matthew H Bailey[138,139], Mattia Bosio[46,136,147], G Steven Bova[330], Alvis Brazma[7], Alicia L Bruzos[287,288,289], Ivo Buchhalter[52,53,54], Carlos D Bustamante[60,61], Atul J Butte[331], Andy Cafferkey[7], Claudia Calabrese[7,8], Peter J Campbell[1,2], Stephen J Chanock[332], Nilanjan Chatterjee[333,334], Jieming Chen[123,335], Francisco M De La Vega[59,59,60,61,62], Olivier Delaneau[336,337,338], German M Demidov[136,147,339], Anthony DiBiase[340], Li Ding[138,139,146], Oliver Drechsel[136,147], Lewis Jonathan Dursi[9,65], Douglas F Easton[341,342], Serap Erkek[8], Georgia Escaramis[147,343,344], **Xavier Estivill**#[296], Erik Garrison[2], Mark Gerstein[120,121,122,123], Gad Getz[3,4,5,6], Dmitry A Gordenin[297], Nina Habermann[8], Olivier Harismendy[73], Eoghan Harrington[345], Shuto Hayashi[87], José María Heredia-Genestar[346], Aliaksei Z Holik[147], Xing Hua[332], Kuan-lin Huang[139,347], Seiya Imoto[86,87], Sissel Juul[345], Ekta Khurana[117,118,130,131], Hyung-Lae Kim[27], Youngwook Kim[96,97], Leszek J Klimczak[306], **Jan O Korbel**#[7,8], Roelof Koster[348], Sushant Kumar[122,123], Ivica Letunic[155], Yilong Li[2], Tomas Marques-Bonet[111,135,346,349], R Jay Mashl[139,156], Simon Mayes[350], Michael D McLellan[138,139,146], Lisa Mirabello[332], Francesc Muyas[136,147,339], Hidewaki Nakagawa[48], Arcadi Navarro[111,135,346], Steven J Newhouse[7], Stephan Ossowski[136,147,157], Esa Pitkänen[8], Aparna Prasad[136], Raquel Rabionet[136,147,351], Benjamin Raeder[8], Tobias Rausch[8], Steven A Roberts[313], Bernardo Rodriguez-

Martin[287,288,289], Gunnar Rätsch[90,93,94,107,108,109], Natalie Saini[297], Matthias Schlesner[52,110], Roland F Schwarz[7,84,250,260], Ayellet V Segre[3,3,352], Tal Shmaya[59], Suyash S Shringarpure[61], Nikos Sidiropoulos[113], Reiner Siebert[261,262], Jared T Simpson[9,160], Lei Song[332], Oliver Stegle[7,8,265], Hana Susak[136,147], Tomas J Tanskanen[353], Grace Tiao[3], Marta Tojo[289], Jose MC Tubio[287,288,289], Daniel J Turner[350], Lara Urban[7,8], Sebastian M Waszak[8], David C Wedge[2,354,355], Joachim Weischenfeldt[8,113,114], David A Wheeler[164,165], Mark H Wright[61], Dai-Ying Wu[59], Tian Xia[356], Sergei Yakneen[8], Kai Ye[161,166], Venkata D Yellapantula[167,168], Jorge Zamora[2,287,288,289] and Bin Zhu[332]

Tumor subtypes and clinical translation

Fatima Al-Shahrour[357], Gurnit Atwal[9,10,358], Peter J Bailey[236], **Andrew V Biankin#**[359,360,361,362], Paul C Boutros[9,133,142,143], Peter J Campbell[1,2], David K Chang[360,362], Susanna L Cooke[362], Vikram Deshpande[106], Bishoy M Faltas[109], William C Faquin[106], **Levi Garraway#**[49], Gad Getz[3,4,5,6], **Sean M Grimmond#**[363], Syed Haider[9], **Katherine A Hoadley#**[247,248], Wei Jiao[9], Vera B Kaiser[364], Rosa Karlić[365], Mamoru Kato[366], Kirsten Kübler[3,6,106], Alexander J Lazar[367], Constance H Li[9,133], David N Louis[106], Adam Margolin[368], Sancha Martin[2,369], Hardeep K Nahal-Bose[45], G Petur Nielsen[106], Serena Nik-Zainal[2,324,325,326], Larsson Omberg[100], Christine P'ng[9], Marc D Perry[45,103], Paz Polak[3,4,6], Esther Rheinbay[3,6,106], Mark A Rubin[131,194,210,211,212], Colin A Semple[364], Dennis C Sgroi[106], Tatsuhiro Shibata[33,34], Reiner Siebert[261,262], Jaclyn Smith[368], **Lincoln D Stein#**[9,10], Miranda D Stobbe[135,136], Ren X Sun[9], Kevin Thai[45], Derek W Wright[370,371], Chin-Lee Wu[106], Ke Yuan[294,369,372] and Junjun Zhang[45]

Evolution and heterogeneity

David J Adams[2], Pavana Anur[373], Rameen Beroukhim[3,6,172], Paul C Boutros[9,133,142,143], David D L Bowtell[187,291], Peter J Campbell[1,2], Shaolong Cao[148], Elizabeth L Christie[187], Marek Cmero[374,375,376], Yupeng Cun[377], Kevin J Dawson[2], Jonas Demeulemeester[63,64], Stefan C Dentro[2,64,354], Amit G Deshwar[378], Nilgun Donmez[151,159], Ruben M Drews[294], Roland Eils[52,54,66,67], Yu Fan[148], Matthew W Fittall[64], Dale W Garsed[187,188], Moritz Gerstung[7,8], Gad Getz[3,4,5,6], Santiago Gonzalez[7,8], Gavin Ha[3], Kerstin Haase[64], Marcin Imielinski[299,300], Lara Jerman[8,379], Yuan Ji[380,381], Clemency Jolly[64], Kortine Kleinheinz[52,54], Juhee Lee[382], Henry Lee-Six[2], Ignaty Leshchiner[3], Dimitri Livitz[3], Geoff Macintyre[294], Salem Malikic[151,159], Florian Markowetz[294,295], Iñigo Martincorena[2], Thomas J Mitchell[2,295,383], Quaid D Morris[358,384], Ville Mustonen[320,321,322], Layla Oesper[385], Martin Peifer[377], Myron Peto[386], Benjamin J Raphael[120], Daniel Rosebrock[3], Yulia Rubanova[160,358], S Cenk Sahinalp[151,158,159], Adriana Salcedo[9], Matthias Schlesner[52,110], Steven E Schumacher[3,217], Subhajit Sengupta[387], Ruian Shi[384], Seung Jun Shin[264], **Paul T Spellman#**[388], Oliver Spiro[3], Lincoln D Stein[9,10], Maxime Tarabichi[2,64], **Peter Van Loo#**[63,64], Shankar Vembu[384,389], Ignacio Vázquez-García[2,167,327,328], Wenyi Wang[148], **David C Wedge#**[2,354,355], David A Wheeler[164,165], Jeffrey A Wintersinger[192,358,390], Tsun-Po Yang[377], Xiaotong Yao[299,316], Kaixian Yu[391], Ke Yuan[294,369,372] and Hongtu Zhu[392,393]

Exploratory: portals, visualization and software infrastructure

Fatima Al-Shahrour[357], Elisabet Barrera[7], Wojciech Bazant[7], Alvis Brazma[7], Isidro Cortés-Ciriano[237,238,239], Brian Craft[240], David Craft[3], Vincent Ferretti[45,69], Nuno A Fonseca[7,70], Anja Füllgrabe[7], Mary J Goldman[240], **David Haussler#**[240,394], Wolfgang Huber[8], Maria Keays[7], Alfonso Muñoz[7], Brian D O'Connor[45,50], Irene Papatheodorou[7], Robert Petryszak[7], Elena Piñeiro-Yáñez[357], Alfonso Valencia[105,111], **Miguel Vazquez#**[105,112], John N Weinstein[395,396], Qian Xiang[116], Junjun Zhang[45] and **Jingchun Zhu#**[240]

Exploratory: mitochondrial variants and HLA/immunogenicity

Peter J Campbell[1,2], Yiwen Chen[148], Chad J Creighton[241], Li Ding[138,139,146], Akihiro Fujimoto[48], Masashi Fujita[48], Gad Getz[3,4,5,6], Leng Han[231], Takanori Hasegawa[87], Shuto Hayashi[87], Seiya Imoto[86,87], Young Seok Ju[2,181], Hyung-Lae Kim[27], Youngwook Kim[96,97], Youngil Koh[307,308], Mitsuhiro Komura[87], Jun Li[148], **Han Liang#**[397], Iñigo Martincorena[2], Satoru Miyano[87], Shinichi Mizuno[398], **Hidewaki Nakagawa#**[48], Keunchil Park[206,207], Eigo Shimizu[87], Yumeng Wang[148,399], John N Weinstein[395,396], Yanxun Xu[400], Rui Yamaguchi[87], Fan Yang[384], Yang Yang[231], Christopher J Yoon[181], Sung-Soo Yoon[308], Yuan Yuan[148], Fan Zhang[246] and Zemin Zhang[246,271]

Exploratory: pathogens

Malik Alawi[401,402], Ivan Borozan[9], Daniel S Brewer[403,404], Colin S Cooper[404,405,406], Nikita Desai[45], **Roland Eils#**[52,54,66,67], Vincent Ferretti[45,69], Adam Grundhoff[401,407], Murat Iskar[408], Kortine Kleinheinz[52,54], Peter Lichter[408], **Hidewaki Nakagawa#**[48], Akinyemi I Ojesina[255,256,257], Chandra Sekhar Pedamallu[3,6,172], Matthias Schlesner[52,110], Xiaoping Su[144] and Marc Zapatka[408]

Tumor Specific Providers – Australia (Ovarian cancer)

Kathryn Alsop[409,410], Australian Ovarian Cancer Study Group[187,311,411], **David D L Bowtell#**[187,291], Timothy JC Bruxner[185], Angelika N Christ[185], Elizabeth L Christie[187], Stephen M Cordner[412], Prue A Cowin[187], Ronny Drapkin[413], Dariush Etemadmoghadam[187,188], Sian Fereday[414], Dale W Garsed[187,188], Joshy George[170], Sean M Grimmond[363], Anne Hamilton[187], Oliver Holmes[311,312], Jillian A Hung[415,416], Karin S Kassahn[185,417], Stephen H Kazakoff[311,312], Catherine J Kennedy[418,419], Conrad R Leonard[311,312], Linda Mileshkin[187], David K Miller[185,360,420], Gisela Mir Arnau[187], Chris Mitchell[187], Felicity Newell[311,312], Katia Nones[311,312], Ann-Marie Patch[311,312], John V Pearson[311,312], Michael C Quinn[311,312], Mark Shackleton[188,218], Darrin F Taylor[185], Heather Thorne[187], Nadia Traficante[187], Ravikiran Vedururu[187], Nick M Waddell[312], Nicola Waddell[311,312], Paul M Waring[253], Scott Wood[311,312], Qinying Xu[311,312] and Anna deFazio[421,422,423]

Tumor Specific Providers – Australia (Pancreatic cancer)

Matthew J Anderson[185], Davide Antonello[424], Andrew P Barbour[425,426], Claudio Bassi[424], Samantha Bersani[427], **Andrew V Biankin#**[359,360,361,362], Timothy JC Bruxner[185], Ivana

Cataldo[427,428], David K Chang[360,362], Lorraine A Chantrill[360], Yoke-Eng Chiew[421], Angela Chou[360,429], Angelika N Christ[185], Sara Cingarlini[37], Nicole Cloonan[430], Vincenzo Corbo[428,431], [432], Fraser R Duthie[433,434], J Lynn Fink[105,185], Anthony J Gill[360,435], Janet S Graham[362,436], **Sean M Grimmond#**[363], Ivon Harliwong[185], Oliver Holmes[311,312], Nigel B Jamieson[361,362,437], Amber L Johns[360,420], Karin S Kassahn[185,417], Stephen H Kazakoff[311,312], James G Kench[360,435,438], Luca Landoni[424], Rita T Lawlor[428], Conrad R Leonard[311,312], Andrea Mafficini[428], Neil D Merrett[424,439], David K Miller[185,360,420], Marco Miotto[424], Elizabeth A Musgrove[362], Adnan M Nagrial[360], Felicity Newell[311,312], Katia Nones[311,312], Karin A Oien[253,440], Marina Pajic[360], Ann-Marie Patch[311,312], John V Pearson[311,312], Mark Pinese[360], Andreia V Pinho[360], Michael C Quinn[311,312], Alan J Robertson[185], Ilse Rooman[360], Borislav C Rusev[428], Jaswinder S Samra[424,435], Maria Scardoni[427], Christopher J Scarlett[360,441], Aldo Scarpa[428], Elisabetta Sereni[424], Katarzyna O Sikora[428], Michele Simbolo[431], Morgan L Taschuk[45], Christopher W Toon[360], Giampaolo Tortora[37,38], Caterina Vicentini[428], Nick M Waddell[312], Nicola Waddell[311,312], Scott Wood[311,312], Jianmin Wu[360], Qinying Xu[311,312] and Nikolajs Zeps[442]

Tumor Specific Providers – Australia (Skin cancer)

Lauri A Aaltonen[443], Andreas Behren[444], Hazel Burke[445], Jonathan Cebon[444], Rebecca A Dagg[446], Ricardo De Paoli-Iseppi[447], Ken Dutton-Regester[311], Matthew A Field[448], Anna Fitzgerald[449], Sean M Grimmond[363], **Nicholas K Hayward#**[311,445], Peter Hersey[445], Oliver Holmes[311,312], Valerie Jakrot[445], Peter A Johansson[311], Hojabr Kakavand[447], Stephen H Kazakoff[311,312], Richard F Kefford[450], Loretta MS Lau[451], Conrad R Leonard[311,312], Georgina V Long[452], **Graham J Mann#**[453,454], Felicity Newell[311,312], Katia Nones[311,312], Ann-Marie Patch[311,312], John V Pearson[311,312], Hilda A Pickett[451], Antonia L Pritchard[311], Gulietta M Pupo[455], Robyn PM Saw[452], Sarah-Jane Schramm[456], **Richard A Scolyer#**[422,452,457,458], Mark Shackleton[188,218], Catherine A Shang[459], Ping Shang[452], Andrew J Spillane[452], Jonathan R Stretch[452], Varsha Tembe[456], John F Thompson[452], Ricardo E Vilain[457], Nick M Waddell[312], Nicola Waddell[311,312], James S Wilmott[452], Scott Wood[311,312], Qinying Xu[311,312] and Jean Y Yang[460]

Tumor Specific Providers – Canada (Pancreatic cancer)

John Bartlett[461,462], Prashant Bavi[463], Ivan Borozan[9], Dianne E Chadwick[464], Michelle Chan-Seng-Yue[463], Sean Cleary[463,465], Ashton A Connor[466,467], Karolina Czajka[468], Robert E Denroche[463], Neesha C Dhani[469], Jenna Eagles[79], Vincent Ferretti[45,69], Steven Gallinger[463,466,467], Robert C Grant[463,470], David Hedley[469], Michael A Hollingsworth[471], **Thomas J Hudson#**[78,79], Gun Ho Jang[463], Jeremy Johns[79], Sangeetha Kalimuthu[463], Sheng-Ben Liang[472], Ilinca Lungu[463,473], Xuemei Luo[9], Faridah Mbabaali[79], **John D McPherson#**[79,463,474], Treasa A McPherson[470], Jessica K Miller[79], Malcolm J Moore[469], Faiyaz Notta[463,475], Danielle Pasternack[79], Gloria M Petersen[476], Michael H A Roehrl[133,463,477,478,479], Michelle Sam[79], Iris Selander[470], Stefano Serra[253], Sagedeh Shahabi[472], **Lincoln D Stein#**[9,10], Morgan L Taschuk[45], Sarah P Thayer[106], Lee E Timms[79], Gavin W Wilson[9,463], Julie M Wilson[463] and Bradly G Wouters[480]

Tumor Specific Providers – Canada (Prostate cancer)

Timothy A Beck[45], Vinayak Bhandari[9], Paul C Boutros[9,133,142,143], **Robert G Bristow#**[133,481,482,483,484], Colin C Collins[151], Shadrielle MG Espiritu[9], Neil E Fleshner[485], Natalie S Fox[9], Michael Fraser[9], Syed Haider[9], Lawrence E Heisler[486], Vincent Huang[9], Emilie Lalonde[9], Julie Livingstone[9], John D McPherson[79,463,474], Alice Meng[487], Veronica Y Sabelnykova[9], Adriana Salcedo[9], Yu-Jia Shiah[9], Theodorus Van der Kwast[488] and Takafumi N Yamaguchi[9]

Tumor Specific Providers – China (Gastric cancer)

Shuai Ding[489], Daiming Fan[490], Yong Hou[39,249], Yi Huang[153,154], Lin Li[39], Siliang Li[39,249], Dongbing Liu[39,249], Xingmin Liu[39,249], **Youyong Lu#**[28,29,30], Yongzhan Nie[490,491], Hong Su[39,249], Jian Wang[39], Kui Wu[39,249], Xiao Xiao[154], Rui Xing[29,492], **Huanming Yang#**[39], Shanlin Yang[489], Yingyan Yu[493], [230], Xiuqing Zhang[39], Yong Zhou[39] and Shida Zhu[39,249]

Tumor Specific Providers – EU: France (Renal cancer)

Rosamonde E Banks[494], Guillaume Bourque[495,496], Alvis Brazma[7], Paul Brennan[497], **Mark Lathrop#**[496], Louis Letourneau[498], Yasser Riazalhosseini[496], Ghislaine Scelo[497], **Jörg Tost#**[499], Naveen Vasudev[500] and Juris Viksna[501]

Tumor Specific Providers – EU: United Kingdom (Breast cancer)

Sung-Min Ahn[502], Ludmil B Alexandrov[2,317], Samuel Aparicio[503], Laurent Arnould[504], MR Aure[505], Shriram G Bhosle[2], E Birney[7], Ake Borg[506], S Boyault[507], AB Brinkman[508], JE Brock[509], A Broeks[510], Adam P Butler[2], AL Børresen-Dale[505], C Caldas[511,512], Peter J Campbell[1,2], Suet-Feung Chin[511,512], Helen Davies[2], C Desmedt[513], L Dirix[514], S Dronov[2], Anna Ehinger[515], JE Eyfjord[516], GG Van den Eynden[517], A Fatima[217], Jorge Reis Filho[518], JA Foekens[519], PA Futreal[520], Øystein Garred[521,522], Moritz Gerstung[7,8], Dilip D Giri[518], D Glodzik[2], Dorthe Grabau[523], Holmfridur Hilmarsdottir[516], GK Hooijer[524], Jocelyne Jacquemier[525], SJ Jang[526], Jon G Jonasson[516], Jos Jonkers[527], HY Kim[525], Tari A King[528,529], Stian Knappskog[2], G Kong[525], S Krishnamurthy[530], S Van Laere[514], SR Lakhani[531], A Langerød[505], Denis Larsimont[532], HJ Lee[526], JY Lee[533], Ming Ta Michael Lee[520], Yilong Li[2], Ole Christian Lingjærde[534], Gaetan MacGrogan[535], JW Martens[536], Sancha Martin[2,369], Iñigo Martincorena[2], Andrew Menzies[2], Sandro Morganella[2], Ville Mustonen[320,321,322], Serena Nik-Zainal[2,324,325,326], Sarah O'Meara[2], I Pauporté[18], Sarah Pinder[537], X Pivot[538], Elena Provenzano[539], CA Purdie[540], Keiran M Raine[2], M Ramakrishna[2], K Ramakrishnan[2], AL Richardson[217], M Ringnér[506], Javier Bartolomé Rodriguez[105], FG Rodríguez-González[175], G Romieu[541], Roberto Salgado[253], Torill Sauer[534], R Shepherd[2], AM Sieuwerts[177], PT Simpson[531], M Smid[542], C Sotiriou[55], PN Span[543], J Staaf[506], Lucy Stebbings[2], Ólafur Andri Stefánsson[544], Alasdair Stenhouse[545], **Michael Rudolf Stratton#**[2], HG Stunnenberg[249,546], Fred Sweep[547], BK Tan[548], Jon W Teague[2], Gilles Thomas[549], AM Thompson[545], S Tommasi[550], I Treilleux[551,552], Andrew Tutt[217], NT Ueno[393], Peter Van Loo[63,64], P Vermeulen[514], Alain Viari[428], MJ van de Vijver[253], A Vincent-Salomon[546],

David C Wedge[2,354,355], Bernice Huimin Wong[553], Lucy Yates[2], X Zou[2], CHM van Deurzen[536] and L van't Veer[554,555]

Tumor Specific Providers – Germany (Malignant lymphoma)

Ole Ammerpohl[556,557], Sietse Aukema[558,559], Anke K Bergmann[560], Stephan H Bernhart[276,277,281], Hans Binder[276,277], Arndt Borkhardt[561], Christoph Borst[562], Benedikt Brors[82,119,278], Birgit Burkhardt[563], Alexander Claviez[564], Roland Eils[52,54,66,67], Maria Elisabeth Goebler[565], Andrea Haake[556], Siegfried Haas[562], Martin Hansmann[566], Jessica I Hoell[561], Steve Hoffmann[277,279,280,281], Michael Hummel[567], Daniel Hübschmann[54,66,83,84,85], Dennis Karsch[568], Wolfram Klapper[559], Kortine Kleinheinz[52,54], Michael Kneba[568], Jan O Korbel[7,8], Helene Kretzmer[277,281], Markus Kreuz[569], Dieter Kube[570], Ralf Küppers[571], Chris Lawerenz[67], Dido Lenze[567], Peter Lichter[408], Markus Loeffler[569], Cristina López[262,556], Luisa Mantovani-Löffler[572], Peter Möller[573], German Ott[574], Bernhard Radlwimmer[408], Julia Richter[556,559], Marius Rohde[575], Philip C Rosenstiel[576], Andreas Rosenwald[577], Markus B Schilhabel[576], Matthias Schlesner[52,110], Stefan Schreiber[578], **Reiner Siebert#**[261,262], Peter F Stadler[276,277,281], Peter Staib[579], Stephan Stilgenbauer[580], Stephanie Sungalee[8], Monika Szczepanowski[559], Umut H Toprak[54,581], Lorenz HP Trümper[570], Rabea Wagener[262,556] and Thorsten Zenz[82]

Tumor Specific Providers – Germany (Pediatric Brain cancer)

Ivo Buchhalter[52,53,54], Juergen Eils[66,67], Roland Eils[52,54,66,67], Volker Hovestadt[408], Barbara Hutter[80,81,82], David TW Jones[301,302], Natalie Jäger[52], Christof von Kalle[84], Marcel Kool[98,301], Jan O Korbel[7,8], Andrey Korshunov[98], Pablo Landgraf[582], Chris Lawerenz[67], Hans Lehrach[583], **Peter Lichter#**[408], Paul A Northcott[584], Stefan M Pfister[98,301,585], Bernhard Radlwimmer[408], Guido Reifenberger[582], Matthias Schlesner[52,110], Hans-Jörg Warnatz[583], Joachim Weischenfeldt[8,113,114], Stephan Wolf[586], Marie-Laure Yaspo[583] and Marc Zapatka[408]

Tumor Specific Providers – Germany (Prostate cancer)

Yassen Assenov[587], Benedikt Brors[82,119,278], Juergen Eils[66,67], Roland Eils[52,54,66,67], Lars Feuerbach[119], Clarissa Gerhauser[285], Jan O Korbel[7,8], Chris Lawerenz[67], Hans Lehrach[583], Sarah Minner[588], Christoph Plass[285], **Guido Sauter#**[589], Thorsten Schlomm[114,590], Nikos Sidiropoulos[113], Ronald Simon[589], **Holger Sültmann#**[82,591], Hans-Jörg Warnatz[583], Dieter Weichenhan[285], Joachim Weischenfeldt[8,113,114] and Marie-Laure Yaspo[583]

Tumor Specific Providers – India (Oral cancer)

Nidhan K Biswas[592], Luca Landoni[424], Arindam Maitra[592], **Partha P Majumder#**[592] and **Rajiv Sarin#**[593]

Tumor Specific Providers – Italy (Pancreatic cancer)

Davide Antonello[424], Stefano Barbi[431], Claudio Bassi[424], Samantha Bersani[427], Giada Bonizzato[428], Cinzia Cantù[428], Ivana Cataldo[427,428], Sara Cingarlini[37], Vincenzo Corbo[428,431], [432], Angelo P Dei Tos[594], Matteo Fassan[595], Sonia Grimaldi[428], Luca Landoni[424], Rita T Lawlor[428], Claudio Luchini[427], Andrea Mafficini[428], Giuseppe Malleo[424], Giovanni Marchegiani[424], Michele Milella[37], Marco Miotto[424], Salvatore Paiella[424], Antonio Pea[424], Paolo Pederzoli[424], Borislav C Rusev[428], Andrea Ruzzenente[424], Roberto Salvia[424], Maria Scardoni[427], **Aldo Scarpa#**[428], Elisabetta Sereni[424], Michele Simbolo[431], Nicola Sperandio[428], Giampaolo Tortora[37,38] and Caterina Vicentini[428]

Tumor Specific Providers – Japan (Biliary tract cancer)

Yasuhito Arai[33], Natsuko Hama[33], Nobuyoshi Hiraoka[596], Fumie Hosoda[33,597], Mamoru Kato[366], Hiromi Nakamura[33], Hidenori Ojima[598], Takuji Okusaka[599], **Tatsuhiro Shibata#**[33,34], Yasushi Totoki[33] and Tomoko Urushidate[34]

Tumor Specific Providers – Japan (Gastric cancer)

Hiroyuki Aburatani#[272], Yasuhito Arai[33], Masashi Fukayama[600], Natsuko Hama[33], Fumie Hosoda[33,597], Shumpei Ishikawa[601], Hitoshi Katai[602], Mamoru Kato[366], Hiroto Katoh[603], Daisuke Komura[601], Genta Nagae[272,284], Hiromi Nakamura[33], Hirofumi Rokutan[604], Mihoko Saito-Adachi[33], **Tatsuhiro Shibata#**[33,34], Akihiro Suzuki[272,605], Hirokazu Taniguchi[606], Kenji Tatsuno[272], Yasushi Totoki[33], Tetsuo Ushiku[600], Shinichi Yachida[33,607] and Shogo Yamamoto[272]

Tumor Specific Providers – Japan (Liver cancer)

Hiroyuki Aburatani[272], Hiroshi Aikata[608], Koji Arihiro[608], Shun-ichi Ariizumi[609], Keith A Boroevich[47,48], Kazuaki Chayama[608], Akihiro Fujimoto[48], Masashi Fujita[48], Mayuko Furuta[48], Kunihito Gotoh[610], Natsuko Hama[33], Takanori Hasegawa[87], Shinya Hayami[611], Shuto Hayashi[87], Satoshi Hirano[612], Seiya Imoto[86,87], Mamoru Kato[366], Yoshiiku Kawakami[608], Kazuhiro Maejima[48], Satoru Miyano[87], Genta Nagae[272,284], **Hidewaki Nakagawa#**[48], Hiromi Nakamura[33], Toru Nakamura[612], Kaoru Nakano[48], Hideki Ohdan[608], Aya Sasaki-Oku[48], **Tatsuhiro Shibata#**[33,34], Yuichi Shiraishi[87], Hiroko Tanaka[87], Yasushi Totoki[33], Tatsuhiko Tsunoda[47,220,221,222], Masaki Ueno[611], Rui Yamaguchi[87], Masakazu Yamamoto[609] and Hiroki Yamaue[611]

Tumor Specific Providers – Singapore (Biliary tract cancer)

Su Pin Choo[613], Ioana Cutcutache[267,319], Narong Khuntikeo[424,614], John R McPherson[267,319], Choon Kiat Ong[615], Chawalit Pairojkul[253], Irinel Popescu[616], **Steven G Rozen#**[267,268,319], **Patrick Tan#**[254,266,267,268] and **Bin Tean Teh#**[266,267,268,269,270]

Tumor Specific Providers – South Korea (Blood cancer)

Keun Soo Ahn[617], Hyung-Lae Kim[27], Youngil Koh[307,308] and **Sung-Soo Yoon#**[308]

Tumor Specific Providers – Spain (Chronic Lymphocytic Leukemia)

Marta Aymerich[618], **Elias Campo#**[619,620], Josep Ll Gelpi[46,71], Ivo G Gut[135,136], Marta Gut[135,136], Armando Lopez-Guillermo[621], Carlos López-Otín[622], Xose S Puente[623], Romina Royo[105] and David Torrents[105,111]

Tumor Specific Providers – United Kingdom (Bone cancer)

Fernanda Amary[624], Daniel Baumhoer[625], Sam Behjati[2], Bodil Bjerkehagen[626], **Peter J Campbell#**[1,2], **Adrienne M Flanagan#**[627], PA Futreal[520], Ola Myklebost[628], Nischalan Pillay[629], Patrick Tarpey[630], Roberto Tirabosco[631] and Olga Zaikova[632]

Tumor Specific Providers – United Kingdom (Chronic myeloid disorders)

Jacqueline Boultwood[633], David T Bowen[2], Adam P Butler[2], **Peter J Campbell#**[1,2], Mario Cazzola[634], Carlo Gambacorti-Passerini[186], Anthony R Green[295], Eva Hellstrom-Lindberg[635], Luca Malcovati[634], Sancha Martin[2,369], Jyoti Nangalia[636], Elli Papaemmanuil[2] and Paresh Vyas[311,637]

Tumor Specific Providers – United Kingdom (Esophageal cancer)

Yeng Ang[638], Hugh Barr[639], Duncan Beardsmore[640], Matthew Eldridge[294], **Rebecca C Fitzgerald#**[325], James Gossage[641], Nicola Grehan[325], George B Hanna[642], Stephen J Hayes[643,644], Ted R Hupp[645], David Khoo[646], Jesper Lagergren[635,647], Laurence E Lovat[251], Shona MacRae[395], Maria O'Donovan[325], J Robert O'Neill[648], Simon L Parsons[649], Shaun R Preston[650], Sonia Puig[651], Tom Roques[652], Grant Sanders[248], Sharmila Sothi[653], Simon Tavaré[294], Olga Tucker[654], Richard Turkington[655], Timothy J Underwood[656] and Ian Welch[657]

Tumor Specific Providers – United Kingdom (Prostate cancer)

Nicholas Van As[658], Daniel M Berney[659], Johann S De Bono[405], G Steven Bova[330], Daniel S Brewer[403,404], Adam P Butler[2], Declan Cahill[658], Niedzica Camacho[405], **Colin S Cooper#**[404,405,406], Nening M Dennis[658], Tim Dudderidge[658], Sandra E Edwards[405], **Rosalind A Eeles#**[405,658], Cyril Fisher[658], Christopher S Foster[660,661], Mohammed Ghori[2], Pelvender Gill[637], Vincent J Gnanapragasam[383,662], Gunes Gundem[2], Freddie C Hamdy[663], Steve Hawkins[294], Steven Hazell[658], William Howat[383], William B Isaacs[292], Katalin Karaszi[637], Jonathan D Kay[251], Vincent Khoo[658], Zsofia Kote-Jarai[405], Barbara Kremeyer[2], Pardeep Kumar[658], Adam Lambert[637], Daniel A Leongamornlert[2,405], Naomi Livni[658], Hayley J Luxton[251], Andy G Lynch[294,295,310], Luke Marsden[637],

Charlie E Massie[294], Lucy Matthews[405], Erik Mayer[658,664], Ultan McDermott[2], Sue Merson[405], Thomas J Mitchell[2,295,383], David E Neal[294,383], Anthony Ng[665], David Nicol[658], Christopher Ogden[658], Edward W Rowe[658], Nimish C Shah[383], Jon W Teague[2], Sarah Thomas[658], Alan Thompson[658], Peter Van Loo[63,64], Clare Verrill[637,666], Tapio Visakorpi[330], Anne Y Warren[383,667], David C Wedge[2,354,355], Hayley C Whitaker[251], Yong-Jie Yu[659], Yongwei Yu[230], Jorge Zamora[2,287,288,289] and Hongwei Zhang[230]

Tumor Specific Providers – United States (TCGA)

Adam Abeshouse[199], Nishant Agrawal[72], Rehan Akbani[325,668], Hikmat Al-Ahmadie[199], Monique Albert[462], Kenneth Aldape[253,646,669], Adrian Ally[670], Yeng Ang[638], Elizabeth L Appelbaum[139,251], Joshua Armenia[671], Sylvia Asa[649,672], J Todd Auman[673], Matthew H Bailey[138,139], Miruna Balasundaram[670], Saianand Balu[248], Jill Barnholtz-Sloan[674,675], Hugh Barr[639], John Bartlett[461,462], Oliver F Bathe[676,677], Stephen B Baylin[656,678], Duncan Beardsmore[640], Christopher Benz[679], Andrew Berchuck[680], Benjamin P Berman[273,274,275], Rameen Beroukhim[3,6,172], Mario Berrios[681], Darell Bigner[294,682], Michael Birrer[106], Tom Bodenheimer[248], Lori Boice[651], Moiz S Bootwalla[683], Marcus Bosenberg[684], Reanne Bowlby[670], Jeffrey Boyd[685], Russell R Broaddus[669], Malcolm Brock[686], Denise Brooks[670], Susan Bullman[3,172], Samantha J Caesar-Johnson[40], Thomas E Carey[687], Rebecca Carlsen[670], Robert Cerfolio[688], Vishal S Chandan[689], Hsiao-Wei Chen[638,671], Andrew D Cherniack[3,3,49,172], Jeremy Chien[690], Juok Cho[3], Eric Chuah[670], Carrie Cibulskis[3], Kristian Cibulskis[3], Leslie Cope[691], Matthew G Cordes[139,652], Kyle Covington[165], Erin Curley[692], Bogdan Czerniak[646,669], Ludmila Danilova[691], Ian J Davis[693], Timothy Defreitas[3], John A Demchok[40], Noreen Dhalla[670], Rajiv Dhir[694], Li Ding[138,139,146], HarshaVardhan Doddapaneni[165], Adel El-Naggar[646,669], Ina Felau[40], Martin L Ferguson[695], Gaetano Finocchiaro[696], Kwun M Fong[697], Scott Frazer[3], William Friedman[698], Catrina C Fronick[139,652], Lucinda A Fulton[139], Robert S Fulton[138,139,146], Stacey B Gabriel[3], Jianjiong Gao[671], Nils Gehlenborg[3,699], Jeffrey E Gershenwald[700,701], Gad Getz[3,4,5,6], Ronald Ghossein[518], Nasra H Giama[702], Richard A Gibbs[165], Carmen Gomez[703], James Gossage[641], Ramaswamy Govindan[138], Nicola Grehan[325], George B Hanna[642], D Neil Hayes[248,704,705], Stephen J Hayes[643,644], Apurva M Hegde[395,668], David I Heiman[3], Zachary Heins[199], Austin J Hepperla[248], Katherine A Hoadley[247,248], Andrea Holbrook[706], Robert A Holt[670], Alan P Hoyle[248], Ralph H Hruban[707], Jianhong Hu[165], Mei Huang[651], David Huntsman[708], Ted R Hupp[645], Jason Huse[199], **Carolyn M Hutter#**[21], Christine A Iacobuzio-Donahue[518], Michael Ittmann[709,710], Joy C Jayaseelan[165], Stuart R Jefferys[248], Corbin D Jones[711], Steven JM Jones[712], Hartmut Juhl[713], Koo Jeong Kang[714], Beth Karlan[715], Katayoon Kasaian[716], Electron Kebebew[717,718], David Khoo[646], Hark Kim[31], Jaegil Kim[3], Tari A King[528,529], Viktoriya Korchina[165], Ritika Kundra[638,671], Jesper Lagergren[635,647], Phillip H Lai[706], Peter W Laird[282], Eric Lander[3], Michael S Lawrence[3,47,106], Alexander J Lazar[367], Xuan Le[719], Darlene Lee[670], Douglas A Levine[199,720], Lora Lewis[165], Tim Ley[721], Haiyan Irene Li[670], Pei Lin[3], W Marston Linehan[722], Eric Minwei Liu[117,118,199], Fei Fei Liu[384], Laurence E Lovat[251], Yiling Lu[723], Lisa Lype[724], Yussanne Ma[670], Shona MacRae[395], Dennis T Maglinte[706], Elaine R Mardis[139,685,725], Jeffrey Marks[424,726], Marco A Marra[670], Thomas J Matthew[50], Michael Mayo[670], Karen McCune[727], Michael D McLellan[138,139,146], Samuel R Meier[3], Shaowu Meng[248], Matthew Meyerson[3,6,49,177,253], Piotr A Mieczkowski[247], Tom Mikkelsen[728], Christopher A Miller[139], Gordon B Mills[368,395,668], Richard A

Moore[670], Carl Morrison[253,729], Lisle E Mose[248], Catherine D Moser[702], Andrew J Mungall[670], Karen Mungall[670], David Mutch[730], Donna M Muzny[165], Jerome Myers[731], Yulia Newton[50], Michael S Noble[3], Peter O'Donnell[732], Brian Patrick O'Neill[733], Angelica Ochoa[199], Akinyemi I Ojesina[255,256,257], Joong Won Park[31], Joel S Parker[734], Simon L Parsons[649], Harvey Pass[735], Alessandro Pastore[90], Chandra Sekhar Pedamallu[3,6,172], Nathan A Pennell[736], Charles M Perou[737], Gloria M Petersen[476], Nicholas Petrelli[738], Olga Potapova[739], Shaun R Preston[650], Sonia Puig[651], Janet S Rader[740], Suresh Ramalingam[741], W Kimryn Rathmell[742], Victor Reuter[253,518], Sheila M Reynolds[724], Matthew Ringel[743], Jeffrey Roach[744], Lewis R Roberts[702], A Gordon Robertson[670], Tom Roques[652], Mark A Rubin[131,194,210,211,212], Sara Sadeghi[670], Gordon Saksena[3], Charles Saller[745], Francisco Sanchez-Vega[638,671], Chris Sander[49,90,215,216], Grant Sanders[248], Dirk Schadendorf[80,746], Jacqueline E Schein[670], Heather K Schmidt[139], Nikolaus Schultz[671], Steven E Schumacher[3,217], Richard A Scolyer[422,452,457,458], Raja Seethala[747], Yasin Senbabaoglu[90], Troy Shelton[692], Yan Shi[248], Juliann Shih[3,172,177], Ilya Shmulevich[724], Craig Shriver[748], Sabina Signoretti[172,177,749], Janae V Simons[248], Samuel Singer[424,750], Payal Sipahimalani[670], Tara J Skelly[247], Karen Smith-McCune[727], Nicholas D Socci[90], Heidi J Sofia[21], Matthew G Soloway[734], Anil K Sood[751], Sharmila Sothi[653], Angela Tam[670], Donghui Tan[247], Roy Tarnuzzer[40], Nina Thiessen[670], R Houston Thompson[752], Leigh B Thorne[651], Ming Tsao[649,672], Olga Tucker[654], Richard Turkington[655], Christopher Umbricht[640,753], Timothy J Underwood[656], David J Van Den Berg[681], Erwin G Van Meir[754], Umadevi Veluvolu[247], Douglas Voet[3], Jiayin Wang[139,154,161], Linghua Wang[165], Zhining Wang[40], Paul Weinberger[755], John N Weinstein[395,396], Daniel J Weisenberger[706], Ian Welch[657], David A Wheeler[164,165], Dennis Wigle[756], Matthew D Wilkerson[247], Richard K Wilson[139,757], Boris Winterhoff[758], Maciej Wiznerowicz[759,760], Tina Wong[139,670], Winghing Wong[139], Liu Xi[165], Liming Yang[40], Christina Yau[294,679,680], Venkata D Yellapantula[167,168], **Jean C Zenklusen#**[40], Hailei Zhang[3], Hongxin Zhang[671] and Jiashan Zhang[40]

# Denotes **working group or project co-leader**

Author Affiliations

1. Department of Haematology, University of Cambridge, Cambridge CB2 2XY, UK.
2. Wellcome Sanger Institute, Wellcome Genome Campus, Hinxton, Cambridge, CB10 1SA, UK.
3. Broad Institute of MIT and Harvard, Cambridge, MA 02142, USA.
4. Center for Cancer Research, Massachusetts General Hospital, Boston, MA 02129, USA.
5. Department of Pathology, Massachusetts General Hospital, Boston, MA 02115, USA.
6. Harvard Medical School, Boston, MA 02115, USA.
7. European Molecular Biology Laboratory, European Bioinformatics Institute (EMBL-EBI), Wellcome Genome Campus, Hinxton, Cambridge, CB10 1SD, UK.
8. Genome Biology Unit, European Molecular Biology Laboratory (EMBL), Heidelberg 69117, Germany.
9. Computational Biology Program, Ontario Institute for Cancer Research, Toronto, ON M5G 0A3, Canada.
10. Department of Molecular Genetics, University of Toronto, Toronto, ON M5S 1A8, Canada.
11. Biomolecular Engineering Department, University of California, Santa Cruz, Santa Cruz, CA 95064, USA.

12. King Faisal Specialist Hospital and Research Centre, Al Maather, Riyadh 12713, Saudi Arabia.
13. DLR Project Management Agency, Bonn 53227, Germany.
14. Genome Canada, Ottawa, ON K2P 1P1, Canada.
15. Instituto Carlos Slim de la Salud, Mexico City, Mexico.
16. Federal Ministry of Education and Research, Berlin 10117, Germany.
17. Institut Gustave Roussy, Villejuif 94805, France.
18. Institut National du Cancer (INCA), Boulogne-Billancourt 92100, France.
19. The Wellcome Trust, London NW1 2BE, UK.
20. Prostate Cancer Canada, Toronto, ON M5C 1M1, Canada.
21. National Human Genome Research Institute, National Institutes of Health, Bethesda, MD 20892, USA.
22. Department of Biotechnology, Ministry of Science & Technology, Government of India, New Delhi, Delhi 110003, India.
23. Science Writer, Garrett Park, MD 20896, USA.
24. International Cancer Genome Consortium (ICGC)/ICGC Accelerating Research in Genomic Oncology (ARGO) Secretariat, Toronto, ON M5G 0A3, Canada.
25. Adaptive Oncology Initiative, Ontario Institute for Cancer Research, Toronto, ON M5G 0A3, Canada.
26. Cancer Research UK, London EC1V 4AD, UK.
27. Department of Biochemistry, College of Medicine, Ewha Womans University, Seoul 07895, South Korea.
28. Chinese Cancer Genome Consortium, Shenzhen 518083, China.
29. Laboratory of Molecular Oncology, Beijing,100142, China.
30. Peking University Cancer Hospital & Institute, Key Laboratory of Carcinogenesis and Translational Research (Ministry of Education), Peking University Cancer Hospital & Institute, Beijing 100142, China.
31. National Cancer Center, Tokyo 104-0045, Japan.
32. German Cancer Aid, Bonn 53113, Germany.
33. Division of Cancer Genomics, National Cancer Center Research Institute, Tokyo 104-0045, Japan.
34. Laboratory of Molecular Medicine, Human Genome Center, The Institute of Medical Science, The University of Tokyo, Minato-ku, Tokyo 108-8639, Japan.
35. Japan Agency for Medical Research and Development, Chiyoda-ku, Tokyo 100-0004 Japan.
36. Japan Agency for Medical Research and Development, Chiyoda-ku, Tokyo 100-0004, Japan.
37. Medical Oncology, University and Hospital Trust of Verona, Verona 37134, Italy.
38. University of Verona, Verona 37129, Italy.
39. BGI-Shenzhen, Shenzhen 518083, China.
40. National Cancer Institute, National Institutes of Health, Bethesda, MD 20892, USA.
41. Centre for Law and Genetics, University of Tasmania, Sandy Bay Campus, Hobart, Tasmania 7001 Australia.
42. Centre of Genomics and Policy, McGill University and Génome Québec Innovation Centre, Montreal, QC H3A 1A4, Canada.
43. Heidelberg Academy of Sciences and Humanities, Heidelberg 69120, Germany.
44. CAPHRI Research School, Maastricht University, Maastricht, ER 6229, The Netherlands.

45. Genome Informatics Program, Ontario Institute for Cancer Research, Toronto, ON M5G 0A3, Canada.
46. Barcelona Supercomputing Center (BSC), Barcelona 08034, Spain.
47. Laboratory for Medical Science Mathematics, RIKEN Center for Integrative Medical Sciences, Yokohama, Kanagawa 230-0045, Japan.
48. RIKEN Center for Integrative Medical Sciences, Yokohama, Kanagawa 230-0045, Japan.
49. Dana-Farber Cancer Institute, Boston, MA 02215, USA.
50. University of California Santa Cruz, Santa Cruz, CA 95064, USA.
51. Oregon Health and Science University, Portland, OR 97239, USA.
52. Division of Theoretical Bioinformatics, German Cancer Research Center (DKFZ), Heidelberg 69120, Germany.
53. Heidelberg Center for Personalized Oncology (DKFZ-HIPO), German Cancer Research Center, Heidelberg 69120, Germany.
54. Institute of Pharmacy and Molecular Biotechnology and BioQuant, Heidelberg University, Heidelberg 69120, Germany.
55. University of California San Diego, San Diego, CA 92093, USA.
56. PDXen Biosystems Inc, Seoul 4900, South Korea.
57. Electronics and Telecommunications Research Institute, Daejoen 34129, South Korea.
58. Seven Bridges Genomics, Charlestown, MA 02129, USA.
59. Annai Systems, Inc, Carlsbad, CA 92013, USA.
60. Department of Biomedical Data Science, Stanford University School of Medicine, Stanford, CA 94305, USA.
61. Department of Genetics, Stanford University School of Medicine, Stanford, CA 94305, USA.
62. Departments of Genetics and Biomedical Data Science, Stanford University School of Medicine, Stanford, CA 94305, USA.
63. University of Leuven, Leuven B-3000, Belgium.
64. The Francis Crick Institute, London NW1 1AT, UK.
65. The Hospital for Sick Children, Toronto, ON M5G 0A4, Canada.
66. Heidelberg University, Heidelberg 69120, Germany.
67. New BIH Digital Health Center, Berlin Institute of Health (BIH) and Charité - Universitätsmedizin Berlin, Berlin 10117, Germany.
68. Rigshospitalet, Copenhagen 2200, Denmark.
69. Department of Biochemistry and Molecular Medicine, University of Montreal, Montreal, QC H3C 3J7, Canada.
70. CIBIO/InBIO - Research Center in Biodiversity and Genetic Resources, Universidade do Porto, Vairão 4485-601, Portugal.
71. Department Biochemistry and Molecular Biomedicine, University of Barcelona, Barcelona 08028, Spain.
72. University of Chicago, Chicago, IL 60637, USA.
73. Division of Biomedical Informatics, Department of Medicine, & Moores Cancer Center, UC San Diego School of Medicine, San Diego, CA 92093, USA.
74. Children's Hospital of Philadelphia, Philadelphia, PA 19146, USA.
75. Massachusetts General Hospital Center for Cancer Research, Charlestown, MA 02129, USA.
76. University of Melbourne Centre for Cancer Research, University of Melbourne, Melbourne,

VIC 3010, Australia.
77. Syntekabio Inc, Daejon 34025, South Korea.
78. AbbVie, North Chicago, IL 60064, USA.
79. Genomics Program, Ontario Institute for Cancer Research, Toronto, ON M5G 0A3, Canada.
80. German Cancer Consortium (DKTK), Heidelberg 69120, Germany.
81. Heidelberg Center for Personalized Oncology (DKFZ-HIPO), German Cancer Research Center (DKFZ), Heidelberg 69120, Germany.
82. National Center for Tumor Diseases (NCT) Heidelberg, Heidelberg 69120, Germany.
83. Department of Pediatric Immunology, Hematology and Oncology, University Hospital, Heidelberg 69120, Germany.
84. German Cancer Research Center (DKFZ), Heidelberg 69120, Germany.
85. Heidelberg Institute for Stem Cell Technology and Experimental Medicine (HI-STEM), Heidelberg 69120, Germany.
86. Institute of Medical Science, University of Tokyo, Tokyo 108-8639, Japan.
87. The Institute of Medical Science, The University of Tokyo, Tokyo 108-8639, Japan.
88. Seven Bridges, Charlestown, MA 02129, USA.
89. Genome Integration Data Center, Syntekabio, Inc, Daejon, 34025, South Korea.
90. Computational Biology Center, Memorial Sloan Kettering Cancer Center, New York, NY 10065, USA.
91. ETH Zurich, Department of Biology, Zürich 8093, Switzerland.
92. ETH Zurich, Department of Computer Science, Zurich 8092, Switzerland.
93. SIB Swiss Institute of Bioinformatics, Lausanne 1015, Switzerland.
94. University Hospital Zurich, Zurich, 8091, Switzerland.
95. Health Sciences Department of Biomedical Informatics, University of California San Diego, La Jolla, CA 92093, USA.
96. Department of Health Sciences and Technology, Sungkyunkwan University School of Medicine, Seoul 06351, South Korea.
97. Samsung Genome Institute, Seoul 06351, South Korea.
98. Functional and Structural Genomics, German Cancer Research Center (DKFZ), Heidelberg 69120, Germany.
99. Leidos Biomedical Research, Inc, McLean, VA 22102, USA.
100. Sage Bionetworks, Seattle WA 98109, USA.
101. Genome Informatics, Ontario Institute for Cancer Research, Toronto, ON M5G 2C4, Canada.
102. Department of Cell and Systems Biology, University of Toronto, Toronto, ON M5S 3G5, Canada.
103. Department of Radiation Oncology, University of California San Francisco, San Francisco, CA 94518, USA.
104. CSRA Incorporated, Fairfax, VA 22042, USA.
105. Barcelona Supercomputing Center, Barcelona 08034, Spain.
106. Massachusetts General Hospital, Boston, MA 02114, USA.
107. Department of Biology, ETH Zurich, Wolfgang-Pauli-Strasse 27, 8093 Zürich, Switzerland.
108. Department of Computer Science, ETH Zurich, Zurich 8092, Switzerland.
109. Weill Cornell Medical College, New York, NY 10065, USA.

110. Bioinformatics and Omics Data Analytics, German Cancer Research Center (DKFZ), Heidelberg 69120, Germany.
111. Institució Catalana de Recerca i Estudis Avançats (ICREA), Barcelona 08010, Spain.
112. Department of Clinical and Molecular Medicine, Faculty of Medicine and Health Sciences, Norwegian University of Science and Technology, Trondheim 7030, Norway.
113. Finsen Laboratory and Biotech Research & Innovation Centre (BRIC), University of Copenhagen, Copenhagen 2200, Denmark.
114. Department of Urology, Charité Universitätsmedizin Berlin, Berlin 10117, Germany.
115. Department of Biological Oceanography, Leibniz Institute of Baltic Sea Research, Seestraße 15, Rostock 18119, Germany.
116. Ontario Institute for Cancer Research, Toronto, ON M5G 0A3, Canada.
117. Department of Physiology and Biophysics, Weill Cornell Medicine, New York, NY 10065, USA.
118. Institute for Computational Biomedicine, Weill Cornell Medicine, New York, NY 10021, USA.
119. Division of Applied Bioinformatics, German Cancer Research Center (DKFZ), Heidelberg 69120, Germany.
120. Department of Computer Science, Princeton University, Princeton, NJ 08540, USA.
121. Department of Computer Science, Yale University, New Haven, CT 06520, USA.
122. Department of Molecular Biophysics and Biochemistry, Yale University, New Haven, CT 06520, USA.
123. Program in Computational Biology and Bioinformatics, Yale University, New Haven, CT 06520, USA.
124. Department of Internal Medicine, Stanford University, Stanford, CA 94305, USA.
125. Department of Molecular Medicine (MOMA), Aarhus University Hospital, Aarhus N 8200, Denmark.
126. Clinical Bioinformatics, Swiss Institute of Bioinformatics, Geneva 1202, Switzerland.
127. Institute for Pathology and Molecular Pathology, University Hospital Zurich, Zurich 8091, Switzerland.
128. Institute of Molecular Life Sciences, University of Zurich, Zurich 8057, Switzerland.
129. MIT Computer Science and Artificial Intelligence Laboratory, Massachusetts Institute of Technology, Cambridge, MA 02139, USA.
130. Englander Institute for Precision Medicine, Weill Cornell Medicine, New York, NY 10065, USA.
131. Meyer Cancer Center, Weill Cornell Medicine, New York, NY 10065, USA.
132. Bioinformatics Research Centre (BiRC), Aarhus University, Aarhus 8000, Denmark.
133. Department of Medical Biophysics, University of Toronto, Toronto, ON M5S 1A8, Canada.
134. Institute of Molecular Life Sciences and Swiss Institute of Bioinformatics, University of Zurich, Zurich 8057, Switzerland.
135. CNAG-CRG, Centre for Genomic Regulation (CRG), Barcelona Institute of Science and Technology (BIST), Barcelona 08028, Spain.
136. Universitat Pompeu Fabra (UPF), Barcelona 08003, Spain.
137. Office of Cancer Genomics, National Cancer Institute, US National Institutes of Health, Bethesda, MD 20892, USA.

138. Alvin J. Siteman Cancer Center, Washington University School of Medicine, St Louis, MO 63110, USA.
139. The McDonnell Genome Institute at Washington University, St Louis, MO 63108, USA.
140. Computer Network Information Center,Chinese Academy of Sciences, Beijing 100190, China.
141. Center for Digital Health, Berlin Institute of Health and Charitè - Universitätsmedizin Berlin, Berlin 10117, Germany.
142. Department of Pharmacology, University of Toronto, Toronto, ON M5S 1A8, Canada.
143. University of California Los Angeles, Los Angeles, CA 90095, USA.
144. University of Texas MD Anderson Cancer Center, Houston, TX 77030, USA.
145. Department of Genetics and Informatics Institute, University of Alabama at Birmingham, Birmingham, AL 35294, USA.
146. Department of Genetics, Department of Medicine, Washington University in St Louis, St Louis, MO 63110, USA.
147. Centre for Genomic Regulation (CRG), The Barcelona Institute of Science and Technology, Barcelona 08003, Spain.
148. Department of Bioinformatics and Computational Biology, The University of Texas MD Anderson Cancer Center, Houston, TX 77030, USA.
149. Beijing Genomics Institute, Shenzhen 518083, China.
150. Department of Urologic Sciences, University of British Columbia, Vancouver, BC V5Z 1M9, Canada.
151. Vancouver Prostate Centre, Vancouver, BC V6H 3Z6, Canada.
152. Division of Life Science and Applied Genomics Center, Hong Kong University of Science and Technology, Clear Water Bay, Hong Kong, China.
153. Geneplus-Shenzhen, Shenzhen 518122, China.
154. School of Computer Science and Technology, Xi'an Jiaotong University, Xi'an 710048, China.
155. Biobyte solutions GmbH, Heidelberg 69126, Germany.
156. Division of Oncology, Washington University School of Medicine, St Louis, MO 63110, USA.
157. Institute of Medical Genetics and Applied Genomics, University of Tübingen, Tübingen 72074, Germany.
158. Indiana University, Bloomington, IN 47405, USA.
159. Simon Fraser University, Burnaby, BC V5A 1S6, Canada.
160. Department of Computer Science, University of Toronto, Toronto, ON M5S 1A8, Canada.
161. School of Electronic and Information Engineering, Xi'an Jiaotong University, Xi'an 710048, China.
162. Department of Genetics, Washington University School of Medicine, St Louis, MO 63110, USA.
163. Department of Mathematics, Washington University in St Louis, St Louis, MO 63130, USA.
164. Department of Molecular and Human Genetics, Baylor College of Medicine, Houston, TX 77030, USA.
165. Human Genome Sequencing Center, Baylor College of Medicine, Houston, TX 77030, USA.
166. The First Affiliated Hospital, Xi'an Jiaotong University, Xi'an 710049, China.
167. Department of Epidemiology and Biostatistics, Memorial Sloan Kettering Cancer Center,

New York, NY 10065, USA.
168. The McDonnell Genome Institute at Washington University, Department of Genetics, Department of Medicine, Siteman Cancer Center, Washington University in St Louis, St Louis, MO 63108, USA.
169. Department of Genomic Medicine, The University of Texas MD Anderson Cancer Center, Houston, TX 77030, USA.
170. The Jackson Laboratory for Genomic Medicine, Farmington, CT 06032, USA.
171. Quantitative & Computational Biosciences Graduate Program, Baylor College of Medicine, Houston, TX 77030, USA.
172. Department of Medical Oncology, Dana-Farber Cancer Institute, Boston, MA 02115, USA.
173. Department of Mathematics, Aarhus University, Aarhus 8000, Denmark.
174. Technical University of Denmark, Lyngby 2800, Denmark.
175. University of Copenhagen, Copenhagen 2200, Denmark.
176. Department for BioMedical Research, University of Bern, Bern 3008, Switzerland.
177. Department of Medical Oncology, Inselspital, University Hospital and University of Bern, Bern 3010, Switzerland.
178. Graduate School for Cellular and Biomedical Sciences, University of Bern, Bern 3012, Switzerland.
179. Department of Genitourinary Medical Oncology - Research, Division of Cancer Medicine, The University of Texas MD Anderson Cancer Center, Houston, TX 77030, USA.
180. Faculty of Biosciences, Heidelberg University, Heidelberg 69120, Germany.
181. Korea Advanced Institute of Science and Technology, Daejeon 34141, South Korea.
182. Institute for Research in Biomedicine (IRB Barcelona), The Barcelona Institute of Science and Technology, Barcelona 8003, Spain.
183. Research Program on Biomedical Informatics, Universitat Pompeu Fabra, Barcelona 08002, Spain.
184. Science for Life Laboratory, Department of Cell and Molecular Biology, Uppsala University, Uppsala SE-75124, Sweden.
185. Queensland Centre for Medical Genomics, Institute for Molecular Bioscience, The University of Queensland, St Lucia, QLD 4072, Australia.
186. University of Milano Bicocca, Monza 20052, Italy.
187. Peter MacCallum Cancer Centre, Melbourne, VIC 3000, Australia.
188. Sir Peter MacCallum Department of Oncology, The University of Melbourne, Melbourne, VIC 3052, Australia.
189. Institut Hospital del Mar d'Investigacions Mèdiques (IMIM), Barcelona 08003, Spain.
190. Institute for Research in Biomedicine (IRB Barcelona), Barcelona 08028, Spain.
191. Center for Precision Health, School of Biomedical Informatics, University of Texas Health Science Center, Houston, TX 77030, USA.
192. The Donnelly Centre, University of Toronto, Toronto, ON M5S 3E1, Canada.
193. Health Data Science Unit, University Clinics, Heidelberg 69120, Germany.
194. Department for Biomedical Research, University of Bern, Bern 3008, Switzerland.
195. Research Core Center, National Cancer Centre Korea, Goyang-si 410-769, South Korea.
196. Institute of Computer Science, Polish Academy of Sciences, Warsawa 01-248, Poland.
197. ETH Zurich, Department of Biology, Wolfgang-Pauli-Strasse 27, 8093 Zürich, Switzerland.

198. Harvard University, Cambridge, MA 02138, USA.
199. Memorial Sloan Kettering Cancer Center, New York, NY 10065, USA.
200. Department of Molecular Biophysics and Biochemistry, New Haven, CT 06520, USA.
201. Program in Computational Biology and Bioinformatics, New Haven, CT 06520, USA.
202. Yale University, New Haven, CT 06520, USA.
203. Department of Information Technology, Ghent University, Ghent B-9000, Belgium.
204. Department of Plant Biotechnology and Bioinformatics, Ghent University, Ghent B-9000, Belgium.
205. Yale School of Medicine, Yale University, New Haven, CT 06520, USA.
206. Division of Hematology-Oncology, Samsung Medical Center, Sungkyunkwan University School of Medicine, Seoul, 06351, South Korea.
207. Samsung Advanced Institute for Health Sciences and Technology, Sungkyunkwan University School of Medicine, Seoul 06351, South Korea.
208. Cheonan Industry-Academic Collaboration Foundation, Sangmyung University, Cheonan 31066, South Korea.
209. Spanish National Cancer Research Centre, Madrid 28029, Spain.
210. Bern Center for Precision Medicine, University Hospital of Bern, University of Bern, Bern 3008, Switzerland.
211. Englander Institute for Precision Medicine, Weill Cornell Medicine and NewYork Presbyterian Hospital, New York, NY 10021, USA.
212. Pathology and Laboratory, Weill Cornell Medical College, New York, NY 10021, USA.
213. Vall d'Hebron Institute of Oncology: VHIO, Barcelona 08035, Spain.
214. National Centre for Biological Sciences, Tata Institute of Fundamental Research, Bangalore 560065, India.
215. cBio Center, Dana-Farber Cancer Institute, Harvard Medical School, Boston, MA 02115, USA.
216. Department of Cell Biology, Harvard Medical School, Boston, MA 02115, USA.
217. Department of Cancer Biology, Dana-Farber Cancer Institute, Boston, MA 02215, USA.
218. Peter MacCallum Cancer Centre and University of Melbourne, Melbourne, VIC 3000, Australia.
219. cBio Center, Dana-Farber Cancer Institute, Boston, MA 02215, USA.
220. CREST, Japan Science and Technology Agency, Tokyo 113-0033, Japan.
221. Department of Medical Science Mathematics, Medical Research Institute, Tokyo Medical and Dental University, Bunkyo-ku, Tokyo 113-8510, Japan.
222. Laboratory for Medical Science Mathematics, Department of Biological Sciences, Graduate School of Science, The University of Tokyo, Bunkyo-ku, Tokyo 113-0033, Japan.
223. Science for Life Laboratory, Department of Oncology-Pathology, Karolinska Institutet, Stockholm 17121, Sweden.
224. Department of Gene Technology, Tallinn University of Technology, Tallinn 12616, Estonia.
225. Genetics & Genome Biology Program, SickKids Research Institute, The Hospital for Sick Children, Toronto, ON M5G 1X8, Canada.
226. Department of Information Technology, Ghent University, Interuniversitair Micro-Electronica Centrum (IMEC), Ghent B-9000, Belgium.
227. Science for Life Laboratory, Department of Immunology, Genetics and Pathology, Uppsala

University, Uppsala SE-75108, Sweden.
228. Oregon Health & Sciences University, Portland, OR 97239, USA.
229. Department of Medicine and Therapeutics, The Chinese University of Hong Kong, Shatin, NT, Hong Kong, China SAR.
230. Second Military Medical University, Shanghai 200433, China.
231. The University of Texas Health Science Center at Houston, Houston, TX 77030, USA.
232. Department of Biomedical Informatics, College of Medicine, The Ohio State University, Columbus, OH 43210, USA.
233. The Ohio State University Comprehensive Cancer Center (OSUCCC – James), Columbus, OH 43210, USA.
234. School of Biomedical Informatics, The University of Texas Health Science Center at Houston, Houston, TX 77030, USA.
235. Department of Biochemistry and Molecular Genetics, Feinberg School of Medicine, Northwestern University, Chicago, IL 60637, USA.
236. University of Glasgow, CRUK Beatson Institute for Cancer Research, Bearsden, Glasgow G61 1BD, UK.
237. Centre for Molecular Science Informatics, Department of Chemistry, University of Cambridge, Cambridge CB2 1EW, UK.
238. Department of Biomedical Informatics, Harvard Medical School, Boston, MA 02115, USA.
239. Ludwig Center, Harvard Medical School, Boston, MA 02115, USA.
240. UC Santa Cruz Genomics Institute, University of California Santa Cruz, Santa Cruz, CA 95064, USA.
241. Department of Medicine, Baylor College of Medicine, Houston, TX 77030, USA.
242. Computational and Systems Biology, Genome Institute of Singapore, Singapore 138672, Singapore.
243. School of Computing, National University of Singapore, Singapore 117417, Singapore.
244. The Azrieli Faculty of Medicine, Bar-Ilan University, Safed 13195, Israel.
245. National Cancer Centre Singapore, Singapore 169610, Singapore.
246. Peking University, Beijing 100871, China.
247. Department of Genetics, University of North Carolina at Chapel Hill, Chapel Hill, NC 27599, USA.
248. Lineberger Comprehensive Cancer Center, University of North Carolina at Chapel Hill, Chapel Hill, NC 27599, USA.
249. China National GeneBank-Shenzhen, Shenzhen 518083, China.
250. Berlin Institute for Medical Systems Biology, Max Delbrück Center for Molecular Medicine, Berlin 13125, Germany.
251. University College London, London WC1E 6BT, UK.
252. School of Life Sciences, Peking University, Beijing 100180, China.
253. Department of Pathology, The University of Melbourne, Melbourne, VIC 3052, Australia.
254. Genome Institute of Singapore, Singapore 138672, Singapore.
255. Department of Epidemiology, University of Alabama at Birmingham, Birmingham, AL 35294, USA.
256. HudsonAlpha Institute for Biotechnology, Huntsville, AL 35806, USA.
257. O'Neal Comprehensive Cancer Center, University of Alabama at Birmingham, Birmingham,

AL 35294, USA.
258. Department of Biosciences and Nutrition, Karolinska Institutet, Stockholm 14183, Sweden.
259. Ludwig Center at Harvard, Boston, MA 02115, USA.
260. German Cancer Consortium (DKTK), Partner site Berlin.
261. Finsen Laboratory and Biotech Research & Innovation Centre (BRIC), University of Copenhagen, Kiel 24118, Germany.
262. Institute of Human Genetics, Ulm University and Ulm University Medical Center, Ulm 89081, Germany.
263. Computational & Systems Biology Program, Memorial Sloan Kettering Cancer Center, New York, NY 10065, USA.
264. Korea University, Seoul 02481, South Korea.
265. Division of Computational Genomics and Systems Genetics, German Cancer Research Center (DKFZ), Heidelberg 69120, Germany.
266. Cancer Science Institute of Singapore, National University of Singapore, Singapore 169609, Singapore.
267. Programme in Cancer & Stem Cell Biology, Duke-NUS Medical School, Singapore 169857, Singapore.
268. SingHealth, Duke-NUS Institute of Precision Medicine, National Heart Centre Singapore, Singapore 169609, Singapore.
269. Institute of Molecular and Cell Biology, Singapore 169609, Singapore.
270. Laboratory of Cancer Epigenome, Division of Medical Science, National Cancer Centre Singapore, Singapore 169610, Singapore.
271. BIOPIC, ICG and College of Life Sciences, Peking University, Beijing 100871, China.
272. Genome Science Division, Research Center for Advanced Science and Technology, The University of Tokyo, Tokyo 153-8904, Japan.
273. Center for Bioinformatics and Functional Genomics, Cedars-Sinai Medical Center, Los Angeles, CA 90048, USA.
274. Department of Biomedical Sciences, Cedars-Sinai Medical Center, Los Angeles, CA 90048, USA.
275. The Hebrew University Faculty of Medicine, Jerusalem 91120, Israel.
276. Bioinformatics Group, Department of Computer, University of Leipzig, Leipzig 04109, Germany, Leipzig 04109, Germany.
277. Interdisciplinary Center for Bioinformatics, University of Leipzig, Leipzig 04109, Germany.
278. German Cancer Consortium (DKTK), German Cancer Research Center (DKFZ), Heidelberg 69120, Germany.
279. Bioinformatics Group, Department of Computer Science, University of Leipzig, Leipzig 04109, Germany.
280. Computational Biology, Leibniz Institute on Aging - Fritz Lipmann Institute (FLI), Jena 07745, Germany.
281. Transcriptome Bioinformatics, LIFE Research Center for Civilization Diseases, University of Leipzig, Leipzig 04109, Germany.
282. Center for Epigenetics, Van Andel Research Institute, Grand Rapids, MI 49503, USA.
283. Institut d'Investigacions Biomèdiques August Pi i Sunyer (IDIBAPS), Barcelona 08036, Spain.
284. Research Center for Advanced Science and Technology, The University of Tokyo, Minato-

ku, Tokyo 108-8639, Japan.
285. Cancer Epigenomics, German Cancer Research Center (DKFZ), Heidelberg 69120, Germany.
286. Van Andel Research Institute, Grand Rapids, MI 49503, USA.
287. Department of Zoology, Genetics and Physical Anthropology, Universidade de Santiago de Compostela, Santiago de Compostela 15706, Spain.
288. Centre for Research in Molecular Medicine and Chronic Diseases (CIMUS), Universidade de Santiago de Compostela, Santiago de Compostela 15706, Spain.
289. The Biomedical Research Centre (CINBIO), Universidade de Vigo, Vigo 36310, Spain.
290. Transmissible Cancer Group, Department of Veterinary Medicine, University of Cambridge, Cambridge CB3 0ES, UK.
291. Sir Peter MacCallum Department of Oncology, University of Melbourne, Melbourne, VIC 3052, Australia.
292. Johns Hopkins School of Medicine, Baltimore, MD 21205, USA.
293. University of Ottawa Faculty of Medicine, Department of Biochemistry, Microbiology and Immunology, Ottawa, ON K1H 8M5, Canada.
294. Cancer Research UK Cambridge Institute, University of Cambridge, Cambridge CB2 0RE, UK.
295. University of Cambridge, Cambridge CB2 1TN, UK.
296. Sidra Medicine, Doha 26999, Qatar.
297. Genome Integrity and Structural Biology Laboratory, National Institute of Environmental Health Sciences (NIEHS), Durham, NC 27709, USA.
298. Brandeis University, Waltham, MA 02254, USA.
299. New York Genome Center, New York, NY 10013, USA.
300. Weill Cornell Medicine, New York, NY 10065, USA.
301. Hopp Children's Cancer Center (KiTZ), Heidelberg 69120, Germany.
302. Pediatric Glioma Research Group, German Cancer Research Center (DKFZ), Heidelberg 69120, Germany.
303. Skolkovo Institute of Science and Technology, Moscow 121205, Russia.
304. A.A.Kharkevich Institute of Information Transmission Problems, Moscow 127051, Russia.
305. Dmitry Rogachev National Research Center of Pediatric Hematology, Oncology and Immunology, Moscow, 117997, Russia.
306. Integrative Bioinformatics Support Group, National Institute of Environmental Health Sciences (NIEHS), Durham, NC 27709, USA.
307. Center For Medical Innovation, Seoul National University Hospital, Seoul 03080, South Korea.
308. Department of Internal Medicine, Seoul National University Hospital, Seoul 03080, South Korea.
309. Division of Genetics and Genomics, Boston Children's Hospital and Harvard Medical School, Boston, MA 02115, USA.
310. School of Medicine/School of Mathematics and Statistics, University of St Andrews, St Andrews, Fife KY16 9SS, UK.
311. Department of Genetics and Computational Biology, QIMR Berghofer Medical Research Institute, Brisbane 4006, Australia.
312. Institute for Molecular Bioscience, University of Queensland, St Lucia, Brisbane, QLD 4072,

Australia.
313. School of Molecular Biosciences and Center for Reproductive Biology, Washington State University, Pullman, WA 99164, USA.
314. Cancer Research Institute, Beth Israel Deaconess Medical Center, Boston, MA 02215, USA.
315. Ben May Department for Cancer Research, Department of Human Genetics, The University of Chicago, Chicago, IL 60637, USA.
316. Tri-institutional PhD program of computational biology and medicine, Weill Cornell Medicine, New York, NY 10065, USA.
317. Department of Cellular and Molecular Medicine and Department of Bioengineering and Moores Cancer Center, University of California San Diego, La Jolla, California 92093, USA.
318. Department of Cellular and Molecular Medicine and Department of Bioengineering and Moores Cancer Center, University of California, San Diego, La Jolla, California 92093, USA.
319. Centre for Computational Biology, Duke-NUS Medical School, Singapore 169857, Singapore.
320. Department of Computer Science, University of Helsinki, Helsinki 00014, Finland.
321. Institute of Biotechnology, University of Helsinki, Helsinki 00014, Finland.
322. Organismal and Evolutionary Biology Research Programme, University of Helsinki, Helsinki 00014, Finland.
323. Programme in Cancer & Stem Cell Biology, Centre for Computational Biology, Duke-NUS Medical School, Singapore 169857, Singapore.
324. Academic Department of Medical Genetics, University of Cambridge, Addenbrooke's Hospital, Cambridge CB2 0QQ, UK.
325. MRC Cancer Unit, University of Cambridge, Cambridge CB2 0XZ, UK.
326. The University of Cambridge School of Clinical Medicine, Cambridge CB2 0SP, UK.
327. Department of Applied Mathematics and Theoretical Physics, Centre for Mathematical Sciences, University of Cambridge, Cambridge CB3 0WA, UK.
328. Department of Statistics, Columbia University, New York, NY 10027, USA.
329. Duke-NUS Medical School, Singapore 169857, Singapore.
330. Faculty of Medicine and Health Technology, Tampere University and Tays Cancer Center, Tampere University Hospital, Tampere Fl-33014, Finland.
331. Institute for Computational Health Sciences and Department of Pediatrics, University of California, San Francisco, CA USA.
332. Division of Cancer Epidemiology and Genetics, National Cancer Institute, National Institutes of Health, Bethesda, MD 20892, USA.
333. Department of Biostatistics, Bloomberg School of Public Health, Johns Hopkins University, Baltimore, MD 21287, USA.
334. Department of Oncology, School of Medicine, Johns Hopkins University, Baltimore, MD 21230, USA.
335. Integrated Graduate Program in Physical and Engineering Biology, Yale University, New Haven, CT 06520, USA.
336. Department of Computational Biology, University of Lausanne, Lausanne 1015, Switzerland.
337. Department of Genetic Medicine and Development, University of Geneva Medical School, Geneva CH1211, Switzerland.

338. Swiss Institute of Bioinformatics, University of Geneva, Geneva CH1211, Switzerland.
339. Institute of Medical Genetics and Applied Genomics, University of Tübingen, Tübingen 72076, Germany.
340. Independent Consultant, Wellesley 02481, USA.
341. Centre for Cancer Genetic Epidemiology, Department of Oncology, University of Cambridge, Cambridge CB1 8RN, UK.
342. Centre for Cancer Genetic Epidemiology, Department of Public Health and Primary Care, University of Cambridge, Cambridge CB1 8RN, UK.
343. CIBER Epidemiología y Salud Pública (CIBERESP), Spain.
344. Research Group on Statistics, Econometrics and Health (GRECS), UdG, Barcelona 8041, Spain.
345. Oxford Nanopore Technologies, New York, NY 10013, USA.
346. Institute of Evolutionary Biology (UPF-CSIC), Department of Experimental and Health Sciences, Universitat Pompeu Fabra, Barcelona 08003, Spain.
347. Icahn School of Medicine at Mount Sinai, New York, NY 10029, USA.
348. Laboratory of Translational Genomics, Division of Cancer Epidemiology and Genetics, National Cancer Institute, National Institutes of Health, Bethesda, MD 20892, USA.
349. Institut Català de Paleontologia Miquel Crusafont, Universitat Autònoma de Barcelona, Barcelona 08193, Spain.
350. Applications Department, Oxford Nanopore Technologies, Oxford OX4 4DQ, UK.
351. Institut de Recerca Sant Joan de Déu; Institut de Biomedicina de la Universitat de Barcelona (IBUB) & Department of Genetics, Microbiology & Statistics, Faculty of Biology, University of Barcelona, Barcelona 08028, Spain.
352. Department of Ophthalmology and Ocular Genomics Institute, Massachusetts Eye and Ear, Harvard Medical School, Boston, MA 02114, USA.
353. Department of Medical and Clinical Genetics, Genome-Scale Biology Research Program, University of Helsinki, Helsinki 00100, Finland.
354. Big Data Institute, Li Ka Shing Centre, University of Oxford, Oxford OX3 7LF, UK.
355. Oxford NIHR Biomedical Research Centre, University of Oxford, Oxford OX4 2PG, UK.
356. School of Electronic Information and Communications, Huazhong University of Science and Technology, Wuhan, Hubei 430074, China.
357. Bioinformatics Unit, Spanish National Cancer Research Centre (CNIO), Madrid 28029, Spain.
358. Vector Institute, Toronto, ON M5G 0A3, Canada.
359. South Western Sydney Clinical School, Faculty of Medicine, University of NSW, Liverpool, NSW 2170, Australia.
360. The Kinghorn Cancer Centre, Cancer Division, Garvan Institute of Medical Research, University of NSW, Sydney, NSW 2010, Australia.
361. West of Scotland Pancreatic Unit, Glasgow Royal Infirmary, Glasgow G31 2ER, UK.
362. Wolfson Wohl Cancer Research Centre, Institute of Cancer Sciences, University of Glasgow, Bearsden, Glasgow G61 1QH, UK.
363. University of Melbourne Centre for Cancer Research, The University of Melbourne, Melbourne, VIC 3052, Australia.
364. MRC Human Genetics Unit, MRC IGMM, University of Edinburgh, Edinburgh EH4 2XU, UK.

365. Bioinformatics Group, Division of Molecular Biology, Department of Biology, Faculty of Science, University of Zagreb, Bioinformatics Group, Division of Molecular Biology, Department of Biology, Faculty of Science, University of Zagreb.
366. Department of Bioinformatics, Research Institute, National Cancer Center Japan, Tokyo 104-0045, Japan.
367. Departments of Pathology, Genomic Medicine, and Translational Molecular Pathology, The University of Texas MD Anderson Cancer Center, Houston, TX 77030, USA.
368. Oregon Health & Science University, Portland, OR 97239, USA.
369. University of Glasgow, Glasgow G61 1BD, UK.
370. MRC-University of Glasgow Centre for Virus Research, Glasgow G61 1QH, UK.
371. Wolfson Wohl Cancer Research Centre, Institute of Cancer Sciences, University of Glasgow, Bearsden G61 1QH, United Kingdom.
372. School of Computing Science, University of Glasgow, Glasgow G12 8RZ, UK.
373. Molecular and Medical Genetics, Oregon Health and Science University, Portland, OR 97201, USA.
374. Department of Surgery, University of Melbourne, Parkville VIC 3010, Australia.
375. The Murdoch Children's Research Institute, Royal Children’s Hospital, Parkville, VIC 3052, Australia.
376. Walter + Eliza Hall Institute, Parkville, VIC 3052, Australia.
377. University of Cologne, Cologne 50931, Germany.
378. The Edward S. Rogers Sr. Department of Electrical and Computer Engineering, University of Toronto, Toronto, ON M5S 3G4, Canada.
379. University of Ljubljana, Ljubljana 1000, Slovenia.
380. Research Institute, NorthShore University HealthSystem, Evanston, IL 60201, USA.
381. Department of Public Health Sciences, The University of Chicago, Chicago IL 60637.
382. Department of Statistics, University of California Santa Cruz, Santa Cruz, CA 95064, USA.
383. Cambridge University Hospitals NHS Foundation Trust, Cambridge CB2 0QQ, UK.
384. University of Toronto, Toronto, ON M5G 2M9, Canada.
385. Department of Computer Science, Carleton College, Northfield, MN 55057, USA.
386. Molecular and Medical Genetics, Oregon Health & Science University, Portland, OR 97239, USA.
387. Center for Psychiatric Genetics, NorthShore University HealthSystem, Evanston, IL 60201, USA.
388. Molecular and Medical Genetics, Knight Cancer Institute, Oregon Health & Science University, Portland, OR 97219, USA.
389. Argmix Consulting, North Vancouver BC V7M 2J5, Canada.
390. University of Toronto, Department of Computer Science, Toronto, ON M5S 2E4, Canada.
391. Department of Biostatistics, The University of Texas MD Anderson Cancer Center, Houston, TX 77030, USA.
392. Department of Biostatistics, University of North Carolina at Chapel Hill, Chapel Hill, NC 27599, USA.
393. The University of Texas MD Anderson Cancer Center, Houston, TX 77030, USA.
394. Howard Hughes Medical Institute, University of California Santa Cruz, Santa Cruz, CA 95065, USA.

395. Cancer Unit, MRC University of Cambridge, Cambridge CB2 0XZ, UK.
396. Department of Bioinformatics and Computational Biology and Department of Systems Biology, The University of Texas MD Anderson Cancer Center, Houston, TX 77030, USA.
397. Department of Bioinformatics and Computational Biology, Department of Systems Biology, The University of Texas MD Anderson Cancer Center, Houston, TX 77030, USA.
398. Department of Health Sciences, Faculty of Medical Sciences, Kyushu University, Fukuoka 812-8582, Japan.
399. Baylor College of Medicine, Houston, TX 77030, USA.
400. Department of Applied Mathematics and Statistics, Johns Hopkins University, Baltimore, MD 21218, USA.
401. Heinrich Pette Institute, Leibniz Institute for Experimental Virology, Hamburg 20251, Germany.
402. University Medical Center Hamburg-Eppendorf, Bioinformatics Core, Hamburg 20246, Germany.
403. Earlham Institute, Norwich NR4 7UZ, UK.
404. Norwich Medical School, University of East Anglia, Norwich NR4 7TJ, UK.
405. The Institute of Cancer Research, London SW7 3RP, UK.
406. University of East Anglia, Norwich NR4 7TJ, UK.
407. German Center for Infection Research (DZIF), Partner Site Hamburg-Borstel-Lübeck-Riems, Hamburg, Germany.
408. Division of Molecular Genetics, German Cancer Research Center (DKFZ), Heidelberg 69120, Germany.
409. Peter MacCallum Cancer Centre, Melbourne, Victoria 3000, Australia.
410. Sir Peter MacCallum Department of Oncology, The University of Melbourne, Melbourne VIC 3000, Australia.
411. QIMR Berghofer Medical Research Institute, Brisbane, QLD 4006, Australia.
412. Victorian Institute of Forensic Medicine, Southbank, Victoria 3006, Australia.
413. University of Pennsylvania, Philadelphia, PA 19104, USA.
414. Peter MacCallum Cancer Centre, Melbourne VIC 3000, Australia.
415. Centre for Cancer Research, The Westmead Institute for Medical Research, Sydney 2145, Australia.
416. Department of Gynaecological Oncology, Westmead Hospital, Sydney 2145, Australia.
417. Genetics and Molecular Pathology, SA Pathology, Adelaide, SA 5000, Australia.
418. Centre for Cancer Research, The Westmead Institute for Medical Research, The University of Sydney, Sydney, NSW 2145, Australia.
419. Department of Gynaecological Oncology, Westmead Hospital, Sydney, NSW 2006, Australia.
420. Garvan Institute of Medical Research, Darlinghurst, NSW 2010 Australia.
421. Centre for Cancer Research, The Westmead Institute for Medical Research, and Department of Gynaecological Oncology, Westmead Hospital, Sydney, NSW 2145, Australia.
422. The University of Sydney, Sydney, NSW 2006, Australia.
423. The Westmead Institute for Medical Research. The University of Sydney. The Department of Gynaecological Oncology, Westmead Hospital, Westmead, NSW 2145, Australia.
424. Department of Surgery, Pancreas Institute, University and Hospital Trust of Verona,

Verona 37134, Italy.
425. Department of Surgery, Princess Alexandra Hospital, Woolloongabba QLD 4102, Australia.
426. Surgical Oncology Group, Diamantina Institute, The University of Queensland, Woolloongabba, Brisbane, QLD 4102, Australia.
427. Department of Diagnostics and Public Health, University and Hospital Trust of Verona, Verona 37134, Italy.
428. ARC-Net Centre for Applied Research on Cancer, University and Hospital Trust of Verona, Verona 37134, Italy.
429. Department of Anatomical Pathology, St Vincent's Hospital, Sydney NSW 2010, Australia.
430. School of Biological Sciences, The University of Auckland, Auckland 1010, New Zealand.
431. Department of Pathology and Diagnostics, University and Hospital Trust of Verona, Verona 37134, Italy.
432. Department of Medicine, Section of Endocrinology, University and Hospital Trust of Verona, Verona 37134, Italy.
433. Department of Pathology, Queen Elizabeth University Hospital, Glasgow G51 4TF, UK.
434. Wolfson Wohl Cancer Research Centre, Bearsden, Glasgow G61 1QH, UK.
435. University of Sydney, Sydney, NSW 2006, Australia.
436. Department of Medical Oncology, Beatson West of Scotland Cancer Centre, Glasgow G12 0YN, UK.
437. Academic Unit of Surgery, School of Medicine, College of Medical, Veterinary and Life Sciences, University of Glasgow, Glasgow Royal Infirmary, Glasgow G4 OSF, UK.
438. Tissue Pathology and Diagnostic Oncology, Royal Prince Alfred Hospital, Camperdown, NSW 2050, Australia.
439. Discipline of Surgery, Western Sydney University, Penrith NSW 2751, Australia.
440. Institute of Cancer Sciences, College of Medical Veterinary and Life Sciences, University of Glasgow, Glasgow G12 8QQ, UK.
441. School of Environmental & Life Sciences, University of Newcastle, Ourimbah, NSW 2258, Australia.
442. School of Surgery M507, University of Western Australia, Nedlands 6009, Australia.
443. Applied Tumor Genomics Research Program, Research Programs Unit, University of Helsinki, Helsinki 00290, Finland.
444. Olivia Newton-John Cancer Research Institute, La Trobe University, Heidelberg, Victoria 3084, Australia.
445. Melanoma Institute Australia, The University of Sydney, Wollstonecraft NSW 2065, Australia.
446. Children's Hospital at Westmead, The University of Sydney, Westmead, NSW 2145, Australia.
447. Melanoma Institute Australia, The University of Sydney, Sydney 2065, Australia.
448. Australian Institute of Tropical Health and Medicine, James Cook University, Douglas QLD 4814, Australia.
449. Bioplatforms Australia, North Ryde, NSW 2109, Australia.
450. Melanoma Institute Australia, Macquarie University, Wollstonecraft NSW, 2109, Australia.
451. Children's Medical Research Institute, Westmead, NSW 2145 Australia.
452. Melanoma Institute Australia, The University of Sydney, Wollstonecraft 2065, NSW,

Australia.
453. Westmead Institute for Medical Research, University of Sydney, Westmead, NSW 2145 Australia.
454. Melanoma Institute Australia, The University of Sydney, Wollstonecraft, NSW 2065, Australia.
455. Centre for Cancer Research, The Westmead Millennium Institute for Medical Research, University of Sydney, Westmead Hospital, Westmead NSW 2145, Australia.
456. Centre for Cancer Research, Westmead Institute for Medical Research, Westmead, NSW 2145, Australia.
457. Discipline of Pathology, Sydney Medical School, The University of Sydney, Sydney 2065, Australia.
458. Royal Prince Alfred Hospital, Sydney, NSW 2050, Australia.
459. Bioplatforms Australia, North Ryde, NSW 2109 Australia.
460. School of Mathematics and Statistics, The University of Sydney, Sydney, NSW 2006 Australia.
461. Diagnostic Development, Ontario Institute for Cancer Research, Toronto, ON M5G 0A3, Canada.
462. Ontario Tumour Bank, Ontario Institute for Cancer Research, Toronto, ON M5G 0A3, Canada.
463. PanCuRx Translational Research Initiative, Ontario Institute for Cancer Research, Toronto, ON M5G 0A3, Canada.
464. BioSpecimen Sciences Program, University Health Network, Toronto, ON M5G 2C4, Canada, Toronto, ON M5G 2C4, Canada.
465. Hepatobiliary/Pancreatic Surgical Oncology Program, University Health Network, Toronto, ON M5G 2C4, Canada.
466. Hepatobiliary/pancreatic Surgical Oncology Program, University Health Network, Toronto, ON M5G 2C4, Canada.
467. Lunenfeld-Tanenbaum Research Institute, Mount Sinai Hospital, Toronto, ON M5G 1X5, Canada.
468. Genomics, Ontario Institute for Cancer Research, Toronto, ON M5G 0A3, Canada.
469. Division of Medical Oncology, Princess Margaret Cancer Centre, Toronto, ON M5G 2M9, Canada.
470. Lunenfeld-Tanenbaum Research Institute, Toronto, ON M5G 1X5, Canada.
471. University of Nebraska Medical Centre, Omaha, NE 68198, USA.
472. BioSpecimen Sciences Program, University Health Network, Toronto, ON M5G 2C4, Canada.
473. Transformative Pathology, Ontario Institute for Cancer Research, Toronto, ON M5G 0A3, Canada.
474. Department of Biochemistry and Molecular Medicine, University California at Davis, Sacramento, CA 95817 USA.
475. University Health Network, Princess Margaret Cancer Centre, Toronto, ON M5G 1L7.
476. Department of Health Sciences Research, Mayo Clinic, Rochester, MN 55905, USA.
477. Department of Pathology, Human Oncology and Pathogenesis Program, Memorial Sloan Kettering Cancer Center, New York, NY 10053, USA.

478. Department of Laboratory Medicine and Pathobiology, University of Toronto, Toronto, ON M5S 1A8, Canada.
479. BioSpecimen Sciences, Laboratory Medicine (Toronto), Medical Biophysics, PanCuRX, Toronto, ON M5S 1A8, Canada.
480. Department of Medical Biophysics, University of Toronto, Toronto, ON M5G 1L7, Canada.
481. CRUK Manchester Institute and Centre, Manchester M204GJ, UK.
482. Department of Radiation Oncology, University of Toronto, Toronto, ON M5S 1A8, Canada.
483. Manchester Cancer Research Centre, Cancer Division, FBMH, University of Manchester, Manchester M204GJ, UK.
484. Radiation Medicine Program, Princess Margaret Cancer Centre, Toronto, ON M5G 2M9, Canada.
485. Department of Surgical Oncology, Princess Margaret Cancer Centre, Toronto, ON M5G 2M9, Canada.
486. Genome Informatics Program, Ontario Institute for Cancer Research, Toronto, ON M5G 2C4, Canada.
487. STTARR Innovation Facility, Princess Margaret Cancer Centre, Toronto, ON M5G 1L7, Canada.
488. Department of Pathology, Toronto General Hospital, Toronto, ON M5G 2C4, Canada.
489. Hefei University of Technology, Anhui 230009, China.
490. State key Laboratory of Cancer Biology, and Xijing Hospital of Digestive Diseases, Fourth Military Medical University, Shaanxi 710032, China.
491. Fourth Military Medical University, Shaanxi 710032, China.
492. Peking University Cancer Hospital & Institute, Beijing 100142, China.
493. Department of Surgery, Ruijin Hospital, Shanghai Jiaotong University School of Medicine, Shanghai, 200025, China.
494. Leeds Institute of Medical Research @ St James's, University of Leeds, St James's University Hospital, Leeds LS9 7TF, UK.
495. Canadian Center for Computional Genomics, McGill University, Montreal, QC H3A 0G1, Canada.
496. Department of Human Genetics, McGill University, Montreal, QC H3A 1B1, Canada.
497. International Agency for Research on Cancer, Lyon 69008, France.
498. McGill University and Genome Quebec Innovation Centre, Montreal, QC H3A 0G1, Canada.
499. Centre National de Génotypage, CEA - Institute de Génomique, Evry 91000, France.
500. Leeds Institute of Medical Research @ St James's, University of Leeds, St James's University Hospital, Leeds LS9 7TF, UK.
501. Institute of Mathematics and Computer Science, University of Latvia, Riga LV1459, Latvia.
502. Department of Oncology, Gil Medical Center, Gachon University, Incheon, South Korea.
503. Department of Molecular Oncology, BC Cancer Agency, Vancouver, BC V5Z 1L3, Canada.
504. Los Alamos National Laboratory, Los Alamos, NM 87545, USA.
505. Department of Genetics, Institute for Cancer Research, Oslo University Hospital, The Norwegian Radium Hospital, Oslo O310, Norway.
506. Lund University, Lund 223 62, Sweden.
507. Translational Research Lab, Centre Léon Bérard, Lyon 69373, France.
508. Radboud University, Department of Molecular Biology, Faculty of Science, Nijmegen

Centre for Molecular Life Sciences, Nijmegen 6500 HB, The Netherlands.
509. Department of Pathology, Brigham and Women's Hospital, Harvard Medical School, Boston, MA 02115, USA.
510. Department Experimental Therapy, The Netherlands Cancer Institute, Amsterdam 1066 CX, The Netherlands.
511. Cancer Research UK Cambridge Institute, University of Cambridge, Li Ka Shing Centre, Cambridge CB2 0RE, UK.
512. Department of Oncology, University of Cambridge, Cambridge CB2 1TN, UK.
513. Breast Cancer Translational Research Laboratory JC Heuson, Institut Jules Bordet, Brussels 1000, Belgium.
514. Translational Cancer Research Unit, Center for Oncological Research, Faculty of Medicine and Health Sciences, University of Antwerp, Antwerp 2000, Belgium.
515. Department of Gynecology & Obstetrics, Department of Clinical Sciences, Skåne University Hospital, Lund University, Lund SE-221 85, Sweden.
516. Icelandic Cancer Registry, Icelandic Cancer Society, Reykjavik 125, Iceland.
517. Translational Cancer Research Unit, GZA Hospitals St.-Augustinus, Antwerp 2000, Belgium.
518. Department of Pathology, Memorial Sloan Kettering Cancer Center, New York, NY 10065, USA.
519. Department of Medical Oncology, Josephine Nefkens Institute and Cancer Genomics Centre, Erasmus Medical Center, Rotterdam 3015CN, The Netherlands.
520. National Genotyping Center, Institute of Biomedical Sciences, Academia Sinica, Taipei 115, Taiwan.
521. Department of Pathology, Oslo University Hospital Ulleval, Oslo 0450, Norway.
522. Faculty of Medicine and Institute of Clinical Medicine, University of Oslo, Oslo NO-0316, Norway.
523. Department of Pathology, Skåne University Hospital, Lund University, Lund SE-221 85, Sweden.
524. Department of Pathology, Academic Medical Center, Amsterdam 1105 AZ, The Netherlands.
525. Department of Pathology, College of Medicine, Hanyang University, Seoul 133-791, South Korea.
526. Department of Pathology, Asan Medical Center, College of Medicine, Ulsan University, Songpa-gu, Seoul 05505, South Korea.
527. Netherlands Cancer Institute, Lund University, Lund 223 62, Sweden.
528. Department of Surgery, Memorial Sloan-Kettering Cancer Center, New York, NY 10065, USA.
529. Department of Surgery, Brigham and Women’s Hospital/Dana Farber Cancer Insititute, Boston, MA 02115, USA.
530. Morgan Welch Inflammatory Breast Cancer Research Program and Clinic, The University of Texas MD Anderson Cancer Center, Houston, TX 77030, USA.
531. The University of Queensland Centre for Clinical Research, The Royal Brisbane & Women's Hospital, Herston, QLD 4029, Australia.
532. Department of Pathology, Jules Bordet Institute, Brussels 1000, Belgium.
533. Institute for Bioengineering and Biopharmaceutical Research (IBBR), Hanyang University,

Seoul, South Korea.
534. University of Oslo, Oslo 0316, Norway.
535. Institut Bergonié, Bordeaux 33076, France.
536. Department of Pathology, Erasmus Medical Center Rotterdam, Rotterdam 3015 GD, The Netherlands.
537. Department of Research Oncology, Guy's Hospital, King's Health Partners AHSC, King's College London School of Medicine, London SE1 9RT, UK.
538. University Hospital of Minjoz, INSERM UMR 1098, Besançon 25000, France.
539. Cambridge Breast Unit, Addenbrooke's Hospital, Cambridge University Hospital NHS Foundation Trust and NIHR Cambridge Biomedical Research Centre, Cambridge CB2 2QQ, UK.
540. East of Scotland Breast Service, Ninewells Hospital, Aberdeen AB25 2XF, UK.
541. Oncologie Sénologie, ICM Institut Régional du Cancer, Montpellier 34298, France.
542. Los Almos National Laboratory, Los Alamos, NM 87545, USA.
543. Department of Radiation Oncology, Radboud University Medical Centre, Nijmegen 6525 GA, The Netherlands.
544. University of Iceland, Reykjavik 101, Iceland.
545. Dundee Cancer Centre, Ninewells Hospital, Dundee DD2 1SY, UK.
546. Institut Curie, INSERM Unit 830, Paris 75248, France.
547. Department of Laboratory Medicine, Radboud University Nijmegen Medical Centre, Nijmegen GA 6525, The Netherlands.
548. Department of General Surgery, Singapore General Hospital, Outram Rd, Singapore 169608, Singapore.
549. Universite Lyon, INCa-Synergie, Centre Léon Bérard, Lyon 69008, France.
550. Giovanni Paolo II / I.R.C.C.S. Cancer Institute, Bari BA 70124, Italy.
551. Department of Biopathology, Centre Léon Bérard, Lyon 69008, France.
552. Université Claude Bernard Lyon 1, Villeurbanne 69100, France.
553. NCCS-VARI Translational Research Laboratory, National Cancer Centre Singapore, Singapore 169610, Singapore.
554. Division of Molecular Carcinogenesis, The Netherlands Cancer Institute, Amsterdam 1066 CX, The Netherlands.
555. Division of Molecular Carcinogenesis, The Netherlands Cancer Institute, Amsterdam, The Netherlands.
556. Institute of Human Genetics, Christian-Albrechts-University, Kiel 24118, Germany.
557. Institute of Human Genetics, Ulm University and Ulm University Medical Center of Ulm, Ulm 89081, Germany.
558. Institute of Human Genetics, University of Ulm and University Hospital of Ulm, Ulm 89081, Germany.
559. Hematopathology Section, Institute of Pathology, Christian-Albrechts-University, Kiel 24118, Germany.
560. Department of Human Genetics, Hannover Medical School, Hannover 30625, Germany.
561. Department of Pediatric Oncology, Hematology and Clinical Immunology, Heinrich-Heine-University, Düsseldorf 40225, Germany.
562. Department of Internal Medicine/Hematology, Friedrich-Ebert-Hospital, Neumünster 24534, Germany.

563. University Hospital Muenster - Pediatric Hematology and Oncology, Muenster 24534, Germany.
564. Department of Pediatrics, University Hospital Schleswig-Holstein, Kiel 24105, Germany.
565. Department of Medicine II, University of Würzburg, Würzburg, Germany.
566. Senckenberg Institute of Pathology, University of Frankfurt Medical School, Frankfurt 60596, Germany.
567. Institute of Pathology, Charité – University Medicine Berlin, Berlin 10117, Germany.
568. Department for Internal Medicine II, University Hospital Schleswig-Holstein, Kiel 24105, Germany.
569. Institute for Medical Informatics Statistics and Epidemiology, University of Leipzig, Leipzig 04109, Germany.
570. Department of Hematology and Oncology, Georg-Augusts-University of Göttingen, Göttingen 37073, Germany.
571. Institute of Cell Biology (Cancer Research), University of Duisburg-Essen, Essen D-45147, Germany.
572. MVZ Department of Oncology, PraxisClinic am Johannisplatz, Leipzig 04109, Germany.
573. Institute of Pathology, Ulm University and University Hospital of Ulm, Ulm 89081, Germany.
574. Department of Pathology, Robert-Bosch-Hospital, Stuttgart, Germany, Stuttgart 70376, Germany.
575. University Hospital Giessen, Pediatric Hematology and Oncology, Giessen 35392, Germany.
576. Institute of Clinical Molecular Biology, Christian-Albrechts-University, Kiel 24118, Germany.
577. Institute of Pathology, University of Wuerzburg, Wuerzburg 97070, Germany.
578. Department of General Internal Medicine, University Kiel, Kiel 24118, Germany.
579. Clinic for Hematology and Oncology, St.-Antonius-Hospital, Eschweiler D-52249, Germany.
580. Department for Internal Medicine III, University of Ulm and University Hospital of Ulm, Ulm 89081, Germany.
581. Neuroblastoma Genomics, German Cancer Research Center (DKFZ), Heidelberg 69120, Germany.
582. University of Düsseldorf, Düsseldorf 40225, Germany.
583. Department of Vertebrate Genomics/Otto Warburg Laboratory Gene Regulation and Systems Biology of Cancer, Max Planck Institute for Molecular Genetics, Berlin 14195, Germany.
584. St. Jude Children's Research Hospital, Memphis, TN 38105-3678, USA.
585. Heidelberg University Hospital, Heidelberg 69120, Germany.
586. Genomics and Proteomics Core Facility High Throughput Sequencing Unit, German Cancer Research Center (DKFZ), Heidelberg 69120, Germany.
587. Epigenomics and Cancer Risk Factors, German Cancer Research Center (DKFZ), Heidelberg 69120, Germany.
588. University Medical Center Hamburg-Eppendorf, Hamburg 20251, Germany.
589. Institute of Pathology, University Medical Center Hamburg-Eppendorf, Hamburg 20251, Germany.
590. Martini-Clinic, Prostate Cancer Center, University Medical Center Hamburg-Eppendorf, Hamburg 20095, Germany.
591. Division of Cancer Genome Research, German Cancer Research Center (DKFZ), Heidelberg

69120, Germany.
592. National Institute of Biomedical Genomics, Kalyani 741235, West Bengal, India.
593. Advanced Centre for Treatment Research & Education in Cancer, Tata Memorial Centre, Navi Mumbai, Maharashtra 410210, India.
594. Department of Pathology, General Hospital of Treviso, Department of Medicine, University of Padua, Italy, Treviso 31100, Italy.
595. Department of Medicine (DIMED), Surgical Pathology Unit, University of Padua, Padua 35121, Italy.
596. Division of Pathology and Clinical Laboratories, Department of Hepatobiliary and Pancreatic Oncology, Hepatobiliary and Pancreatic Surgery Division, National Cancer Center Hospital, Chuo-ku, Tokyo, 104-0045, Japan.
597. Division of Cancer Genomics, Department of Bioinformatics, National Cancer Center, Tokyo 104-0045, Japan.
598. Department of Pathology, Keio University School of Medicine, Tokyo 160-8582, Japan.
599. Department of Hepatobiliary and Pancreatic Oncology, National Cancer Center Hospital (NCCH), Tokyo, 104-0045 Japan.
600. Department of Pathology, Graduate School of Medicine, The University of Tokyo, Bunkyo-ku, Tokyo 113-0033, Japan.
601. Preventive Medicine, Graduate School of Medicine, The University of Tokyo, Tokyo 113-0033, Japan, Tokyo 113-0033, Japan.
602. Gastric Surgery Division, Division of Pathology and Clinical Laboratories, National Cancer Center Hospital, Tokyo 104-0045, Japan.
603. Department of Preventive Medicine, Graduate School of Medicine, the University of Tokyo, Tokyo 113-0033, Japan.
604. Division of Cancer Genomics, Department of Bioinformatics, National Cancer Center Research Institute, National Cancer Center, Tokyo 104-0045, Japan.
605. Department of Gastroenterology and Hepatology, Yokohama City University Graduate School of Medicine, Kanagawa 236-0004, Japan.
606. Laboratory of Molecular Medicine, Human Genome Center, The Institute of Medical Science, University of Tokyo, Shirokane-dai, Minato-ku, Tokyo 108-8639, Japan.
607. Department of Cancer Genome Informatics, Graduate School of Medicine, Osaka University, Osaka 565-0871, Japan.
608. Hiroshima University, Hiroshima 734-8553, Japan.
609. Tokyo Women’s Medical University, Tokyo 162-8666, Japan.
610. Osaka International Cancer Center, Osaka 541-8567, Japan.
611. Wakayama Medical University, Wakayama 641-8509, Japan.
612. Hokkaido University, Sapporo 060-8648, Japan.
613. Division of Medical Oncology, National Cancer Centre, Singapore 169610, Singapore.
614. Cholangiocarcinoma Screening and Care Program and Liver Fluke and Cholangiocarcinoma Research Centre, Faculty of Medicine, Khon Kaen University, Khon Kaen, Thailand.
615. Lymphoma Genomic Translational Research Laboratory, National Cancer Centre, Singapore 169610, Singapore.
616. Center of Digestive Diseases and Liver Transplantation, Fundeni Clinical Institute, Bucharest 022328, Romania.

617. Division of Hepatobiliary and Pancreatic Surgery, Department of Surgery, School of Medicine, Keimyung University Dongsan Medical Center, Daegu 41931, South Korea.
618. Pathology, Hospital Clinic, Institut d'Investigacions Biomèdiques August Pi i Sunyer (IDIBAPS), Universiy of Barcelona, Barcelona 8034, Spain.
619. Anatomia Patológica, Hospital Clinic, Institut d'Investigacions Biomèdiques August Pi i Sunyer (IDIBAPS), Universiy of Barcelona, Barcelona 8036, Spain.
620. Spanish Ministry of Science and Innovation, Madrid 28046, Spain.
621. Hematology, Hospital Clinic, Institut d'Investigacions Biomèdiques August Pi i Sunyer (IDIBAPS), Universiy of Barcelona, Barcelona 8034, Spain.
622. Departamento de Bioquımicay Biologıa Molecular, Facultad de Medicina, Instituto Universitario de Oncologıa-IUOPA, Oviedo 33006, Spain.
623. Departamento de Bioquímica y Biología Molecular, Instituto Universitario de Oncología (IUOPA), Universidad de Oviedo, Oviedo 33006, Spain.
624. Royal National Orthopaedic Hospital - Bolsover, London W1W 5AQ, UK.
625. Department of Pathology, Oslo University Hospital, Oslo O310, Norway.
626. Department of Pathology, Oslo University Hospital, Norway and University of Oslo, Norway, Oslo O310, Norway.
627. Department of Pathology (Research), University College London Cancer Institute, London WC1E 6BT, UK.
628. Department for Clinical Science, University of Bergen, Bergen 5020, Norway.
629. Research Department of Pathology, University College London Cancer Institute, 72 Huntley Street, London, WC1E 6BT.
630. East Anglian Medical Genetics Service, Cambridge University Hospitals NHS Foundation Trust, Cambridge CB2 0QQ, UK.
631. Royal National Orthopaedic Hospital - Stanmore, Stanmore, Middlesex HA7 4LP, UK.
632. Division of Orthopaedic Surgery, Oslo University Hospital, Oslo 0379, Norway.
633. Radcliffe Department of Medicine, University of Oxford, Oxford OX3 9DU, UK.
634. University of Pavia, Pavia 27100, Italy.
635. Karolinska Institute, Stockholm SE-171 76, Sweden.
636. Wellcome Sanger Institute, Wellcome Genome Campus, Hinxton, Cambridge, CB10 1SD, UK.
637. University of Oxford, Oxford OX3 9DU, UK.
638. Salford Royal NHS Foundation Trust, Salford M6 8HD, UK.
639. Gloucester Royal Hospital, Gloucester, GL1 3NL, UK.
640. Royal Stoke University Hospital, Stoke-on-Trent ST4 6QG, UK.
641. St Thomas's Hospital, London SE1 7EH, UK.
642. Imperial College NHS Trust, Imperial College, London W2 INY, UK.
643. Department of Histopathology, Salford Royal NHS Foundation Trust, Salford M6 8HD, UK.
644. Faculty of Biology, Medicine and Health, University of Manchester, Salford M6 8HD, UK.
645. Edinburgh Royal Infirmary, Edinburgh EH16 4SA, UK.
646. Barking Havering and Redbridge University Hospitals NHS Trust, Romford, RM7 0AG, UK.
647. King's College London and Guy's and St Thomas' NHS Foundation Trust, London SE1 7EH, UK.
648. Cambridge Oesophagogastric Centre, Cambridge University Hospitals NHS Foundation

Trust, Cambridge, CB2 0QQ.
649. Nottingham University Hospitals NHS Trust, Nottingham NG7 2UH, UK.
650. St Luke's Cancer Centre, Royal Surrey County Hospital NHS Foundation Trust, Guildford GU2 7XX, UK.
651. University of North Carolina at Chapel Hill, Chapel Hill, NC 27599, USA.
652. Norfolk and Norwich University Hospital NHS Trust, Norwich NR4 7UY, UK.
653. University Hospitals Coventry and Warwickshire NHS Trust, Coventry CV2 2DX, UK.
654. University Hospitals Birmingham NHS Foundation Trust, Birmingham B15 2GW, UK.
655. Centre for Cancer Research and Cell Biology, Queen's University, Belfast BT9 7AB, UK.
656. University Hospital Southampton NHS Foundation Trust, Southampton, SO16 6YD, UK.
657. Wythenshawe Hospital, Manchester M23 9LT, UK.
658. Royal Marsden NHS Foundation Trust, London and Sutton SW3 6JJ, UK.
659. Barts Cancer Institute, Barts and the London School of Medicine and Dentistry, Queen Mary University of London, London EC1M 6BQ, UK.
660. HCA Laboratories, London W1G 8AQ, UK.
661. University of Liverpool, Liverpool L69 3BX, UK.
662. Academic Urology Group, Department of Surgery, University of Cambridge, Cambridge CB2 0QQ, UK.
663. University of Oxford, Oxford, OX3 9DU, UK.
664. Department of Surgery and Cancer, Imperial College, London W2 INY, UK.
665. The Chinese University of Hong Kong, Shatin, Hong Kong, China.
666. Nuffield Department of Surgical Sciences, John Radcliffe Hospital, University of Oxford, Headington, Oxford OX3 9DU, UK.
667. Department of Histopathology, Cambridge University Hospitals NHS Foundation Trust, Cambridge CB2 0QQ, UK.
668. Department of Bioinformatics and Computational Biology / Department of Systems Biology, The University of Texas MD Anderson Cancer Center, Houston, TX 77030, USA.
669. Department of Pathology, The University of Texas MD Anderson Cancer Center, Houston, TX 77030, USA.
670. Canada's Michael Smith Genome Sciences Center, BC Cancer Agency, Vancouver, BC V5Z 4S6, Canada.
671. Center for Molecular Oncology, Memorial Sloan Kettering Cancer Center, New York, NY 10065, USA.
672. University Health Network, Toronto, ON M5G 2C4, Canada.
673. Department of Pathology and Laboratory Medicine, School of Medicine, University of North Carolina at Chapel Hill, Chapel Hill, NC 27599, USA.
674. Department of Population and Quantitative Health Sciences, Case Western Reserve University School of Medicine, Cleveland, OH 44016, USA.
675. Research Health Analytics and Informatics, University Hospitals Cleveland Medical Center, Cleveland, OH 44106, USA.
676. Arnie Charbonneau Cancer Institute, University of Calgary, Calgary, AB T2N 4N2, Canada.
677. Centre for Cancer Research and Cell Biology, Queens University, Belfast BT9 7AB, UK.
678. Sidney Kimmel Cancer Center, The Johns Hopkins Medical Institutions, Baltimore, MD 21230, USA.

679. Buck Institute for Research on Aging, Novato, CA 94945, USA.
680. Duke University Medical Center, Durham, NC 27710, USA.
681. Norris Comprehensive Cancer Center, University of Southern California, Los Angeles, CA 90033, USA.
682. The Preston Robert Tisch Brain Tumor Center, Duke University Medical Center, Durham, NC 27710, USA.
683. University of Southern California, USC/Norris Comprehensive Cancer Center, Los Angeles, CA 90033, USA.
684. Departments of Dermatology and Pathology, Yale University, New Haven, CT 06510, USA.
685. Fox Chase Cancer Center, Philadelphia, PA 19111, USA.
686. Johns Hopkins University, Baltimore, MD 21287, USA.
687. University of Michigan Comprehensive Cancer Center, Ann Arbor, MI 48109, USA.
688. University of Alabama at Birmingham, Birmingham, AL 35294, USA.
689. Division of Anatomic Pathology, Mayo Clinic, Rochester, MN 55905, USA.
690. Division of Experimental Pathology, Mayo Clinic, Rochester, MN 55905 USA.
691. The Sidney Kimmel Comprehensive Cancer Center at Johns Hopkins University, Baltimore, MD 21287, USA.
692. International Genomics Consortium, Phoenix, AZ 85004, USA.
693. Departments of Pediatrics and Genetics, University of North Carolina at Chapel Hill, Chapel Hill, NC 27599, USA.
694. Department of Pathology, UPMC Shadyside, Pittsburgh, PA 15232, USA.
695. Center for Cancer Genomics, National Cancer Institute, National Institutes of Health, Bethesda, MD 20892, USA.
696. Istituto Neurologico Besta, Department of Neuro-Oncology, Milano 20133, Italy.
697. University of Queensland Thoracic Research Centre, The Prince Charles Hospital, Brisbane, QLD 4032, Australia.
698. Department of Neurosurgery, University of Florida, Gainesville, FL 32610, USA.
699. Center for Biomedical Informatics, Harvard Medical School, Boston, MA 02115, USA.
700. Department of Cancer Biology, The University of Texas MD Anderson Cancer Center, Houston, TX 77030, USA.
701. Department of Surgical Oncology, The University of Texas MD Anderson Cancer Center, Houston, TX 77030, USA.
702. Division of Gastroenterology and Hepatology, Mayo Clinic, Rochester, MN 55905, USA.
703. University of Miami, Sylvester Comprehensive Cancer Center, Miami, FL 33136, USA.
704. Department of Internal Medicine, Division of Medical Oncology, University of North Carolina at Chapel Hill, Chapel Hill, NC 27599, USA.
705. Department of Internal Medicine, Division of Medical Oncology, Lineberger Comprehensive Cancer Center, University of North Carolina at Chapel Hill, Chapel Hill, NC 27599, USA.
706. University of Southern California, Norris Comprehensive Cancer Center, Los Angeles, CA 90033, USA.
707. The Sol Goldman Pancreatic Cancer Research Center, Department of Pathology, Johns Hopkins Hospital, Baltimore, MD 21287, USA.
708. Centre for Translational and Applied Genomics, British Columbia Cancer Agency,

Vancouver, BC V5Z 1L3, Canada.
709. Department of Pathology & Immunology, Baylor College of Medicine, Houston, TX 770230, USA.
710. Michael E. DeBakey Veterans Affairs Medical Center, Houston, TX 770230, USA.
711. Carolina Center for Genome Sciences, University of North Carolina at Chapel Hill, Chapel Hill, NC 27599, USA.
712. Canada's Michael Smith Genome Sciences Centre, BC Cancer, Vancouver, BC V5Z 4S6, Canada.
713. Indivumed GmbH, Hamburg 20251, Germany.
714. Division of Hepatobiliary and Pancreatic Surgery, Department of Surgery, School of Medicine, Keimyung University Dong-san Medical Center, Daegu 41931, South Korea.
715. Women's Cancer Program at the Samuel Oschin Comprehensive Cancer Institute, Cedars-Sinai Medical Center, Los Angeles, CA 90048, USA.
716. Canada's Michael Smith Genome Sciences Centre, BC Cancer Agency, Vancouver, BC V5Z 4S6, Canada.
717. Department of Surgery, The George Washington University, School of Medicine and Health Science, Washington, DC 20052, USA.
718. Endocrine Oncology Branch, Center for Cancer Research, National Cancer Institute, National Institutes of Health, Bethesda, MD 20892, USA.
719. ILSbio, LLC Biobank, Chestertown, MD 21620, USA.
720. Gynecologic Oncology, NYU Laura and Isaac Perlmutter Cancer Center, New York University, New York, NY 10016, USA.
721. Division of Oncology, Stem Cell Biology Section, Washington University School of Medicine, St. Louis, MO 63110, USA.
722. Urologic Oncology Branch, Center for Cancer Research, National Cancer Institute, National Institutes of Health, Bethesda, MD 20892, USA.
723. Department of Systems Biology, University of Texas MD Anderson Cancer Center, Houston, TX 77030, USA.
724. Institute for Systems Biology, Seattle, WA 98109, USA.
725. Institute for Genomic Medicine, Nationwide Children's Hospital, Columbus, OH 43215, USA.
726. Department of Surgery, Duke University, Durham, NC 27710, USA.
727. Department of Obstetrics, Gynecology and Reproductive Services, University of California San Francisco, San Francisco, CA 94143, USA.
728. Departments of Neurology and Neurosurgery, Henry Ford Hospital, Detroit, MI 48202, USA.
729. Department of Pathology, Roswell Park Cancer Institute, Buffalo, NY 14263, USA.
730. Department of Obstetrics and Gynecology, Division of Gynecologic Oncology, Washington University School of Medicine, St. Louis, MO 3110, USA.
731. Penrose St. Francis Health Services, Colorado Springs, CO 80907, USA.
732. The University of Chicago, Chicago, IL 60637, USA.
733. Department of Neurology, Mayo Clinic, Rochester, MN 55905, USA.
734. Department of Genetics and Lineberger Comprehensive Cancer Center, University of North Carolina at Chapel Hill, Chapel Hill, NC 27599, USA.

735. NYU Langone Medical Center, New York, NY 10016, USA.
736. Department of Hematology and Medical Oncology, Cleveland Clinic, Cleveland, OH 44195, USA.
737. Department of Genetics, Department of Pathology and Laboratory Medicine, School of Medicine, University of North Carolina at Chapel Hill, Chapel Hill, NC 27599, USA.
738. Helen F. Graham Cancer Center at Christiana Care Health Systems, Newark, DE 19713, USA.
739. Cureline, Inc, South San Francisco, CA 94080, USA.
740. Department of Obstetrics and Gynecology, Medical College of Wisconsin, Milwaukee, WI 53226, USA.
741. Emory University, Atlanta, GA 30322, USA.
742. Vanderbilt University, Vanderbilt Ingram Cancer Center, Nashville, TN 37232, USA.
743. Ohio State University College of Medicine and Arthur G. James Comprehensive Cancer Center, Columbus, OH 43210, USA.
744. Research Computing Center, University of North Carolina at Chapel Hill, Chapel Hill, NC 27599, USA.
745. Analytical Biological Services, Inc, Wilmington, DE 19801, USA.
746. Department of Dermatology, University Hospital Essen, Westdeutsches Tumorzentrum & German Cancer Consortium, Essen 45122, Germany.
747. University of Pittsburgh, Pittsburgh, PA 15213, USA.
748. Murtha Cancer Center, Walter Reed National Military Medical Center, Bethesda, MD 20889, USA.
749. Brigham and Women's Hospital, Harvard Medical School, Boston, MA 02115, USA.
750. Department of Surgery, Memorial Sloan Kettering Cancer Center, New York, NY 10065, USA.
751. Department of Gynecologic Oncology & Reproductive Medicine, and Center for RNA Interference and Non-Coding RNA, the University of Texas MD Anderson Cancer Center, Houston, TX 77030, USA.
752. Department of Urology, Mayo Clinic, Rochester, MN 55905, USA.
753. Johns Hopkins Medical Institutions, Baltimore, MD 21205, USA.
754. Departments of Neurosurgery and Hematology and Medical Oncology, Winship Cancer Institute and School of Medicine, Emory University, Atlanta, GA 30322, USA.
755. Georgia Regents University Cancer Center, Augusta, GA 30912, USA.
756. Thoracic Oncology Laboratory, Mayo Clinic, Rochester, MN 55905, USA.
757. Institute for Genomic Medicine, Nationwide Children's Hospital, Columbus, OH 43205, USA.
758. Department of Obstetrics & Gynecology, Division of Gynecologic Oncology, Mayo Clinic, Rochester, MN 55905, USA.
759. International Institute for Molecular Oncology, Poznań 60-203, Poland.
760. Poznan University of Medical Sciences, Poznań 61-701, Poland.
