# Supplementary material for: Cancer LncRNA Census reveals evidence for deep functional conservation of long noncoding RNAs in tumorigenesis
Source: Commun Biol. 2020 Feb 5;3:56. doi: 10.1038/s42003-019-0741-7 (PMC7002399; doi:10.1038/s42003-019-0741-7)
Supplement: Supplementary file 2 — Description of additional supplementary items [file 42003_2019_741_MOESM2_ESM.docx]

# **Supplementary Data Legends**

**Supplementary Data 1: full CLC set.**

**Supplementary Data 2: CLC – protein-coding pairs.**

**Supplementary Data 3: GO analysis for protein-coding genes divergent to CLC genes.**

**Supplementary Data 4: GO analysis for protein-coding genes divergent to nonCLC genes.**

**Supplementary Data 5: Counts of mouse CIS per cancer type.**
